# Unveiling a CAAX Protease‐Like Protein Involved in Didemnin Drug Maturation and Secretion

**DOI:** 10.1002/advs.202306044

**Published:** 2023-11-30

**Authors:** Xiaolin Zou, Zhen Hui, Robert A. Shepherd, Shuaiqiang Zhao, Yanfei Wu, Zhuanglin Shen, Cuiping Pang, Shipeng Zhou, Zehai Yu, Jiahai Zhou, Bradly S. Moore, Laura M. Sanchez, Xiaoyu Tang

**Affiliations:** ^1^ Institute of Chemical Biology Shenzhen Bay Laboratory Shenzhen 518132 China; ^2^ Department of Chemistry and Biochemistry University of California Santa Cruz Santa Cruz CA 95064 USA; ^3^ CAS Key Laboratory of Quantitative Engineering Biology Shenzhen Institute of Synthetic Biology Shenzhen Institute of Advanced Technology Chinese Academy of Sciences Shenzhen 518055 China; ^4^ Scripps Institution of Oceanography University of California San Diego La Jolla CA 92093 USA; ^5^ Skaggs School of Pharmacy and Pharmaceutical Sciences University of California San Diego La Jolla CA 92093 USA

**Keywords:** biosynthesis, CAAX proteases, didemnins, nonribosomal peptide synthetase (NRPS), prodrug mechanism

## Abstract

The assembly line biosynthesis of the powerful anticancer‐antiviral didemnin cyclic peptides is proposed to follow a prodrug release mechanism in Tristella bacteria. This strategy commences with the formation of N‐terminal prodrug scaffolds and culminates in their cleavage during the cellular export of the mature products. In this study, a comprehensive exploration of the genetic and biochemical aspects of the enzymes responsible for both the assembly and cleavage of the acylated peptide prodrug scaffolds is provided. This process involves the assembly of N‐acyl‐polyglutamine moieties orchestrated by the nonribosomal peptide synthetase DidA and the cleavage of these components at the post‐assembly stage by DidK, a transmembrane CAAX hydrolase homolog. The findings not only shed light on the complex prodrug mechanism that underlies the synthesis and secretion of didemnin compounds but also offer novel insights into the expanded role of CAAX hydrolases in microbes. Furthermore, this knowledge can be leveraged for the strategic design of genome mining approaches aimed at discovering new bioactive natural products that employ similar prodrug biochemical strategies.

## Graphical abstract

The functions of enzymes involved in both assembling and cleaving the acylated peptide prodrug structures in didemnin biosynthesis are elucidated. This process plays a crucial role in the maturation and secretion of didemnin drugs. The findings not only illuminate the intricate prodrug release mechanism governing the production of didemnin compounds, but also provide new insights into the extended roles of CAAX hydrolases in microbial contexts.

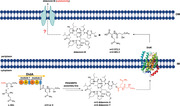

## 1 Introduction

**Figure 1:**
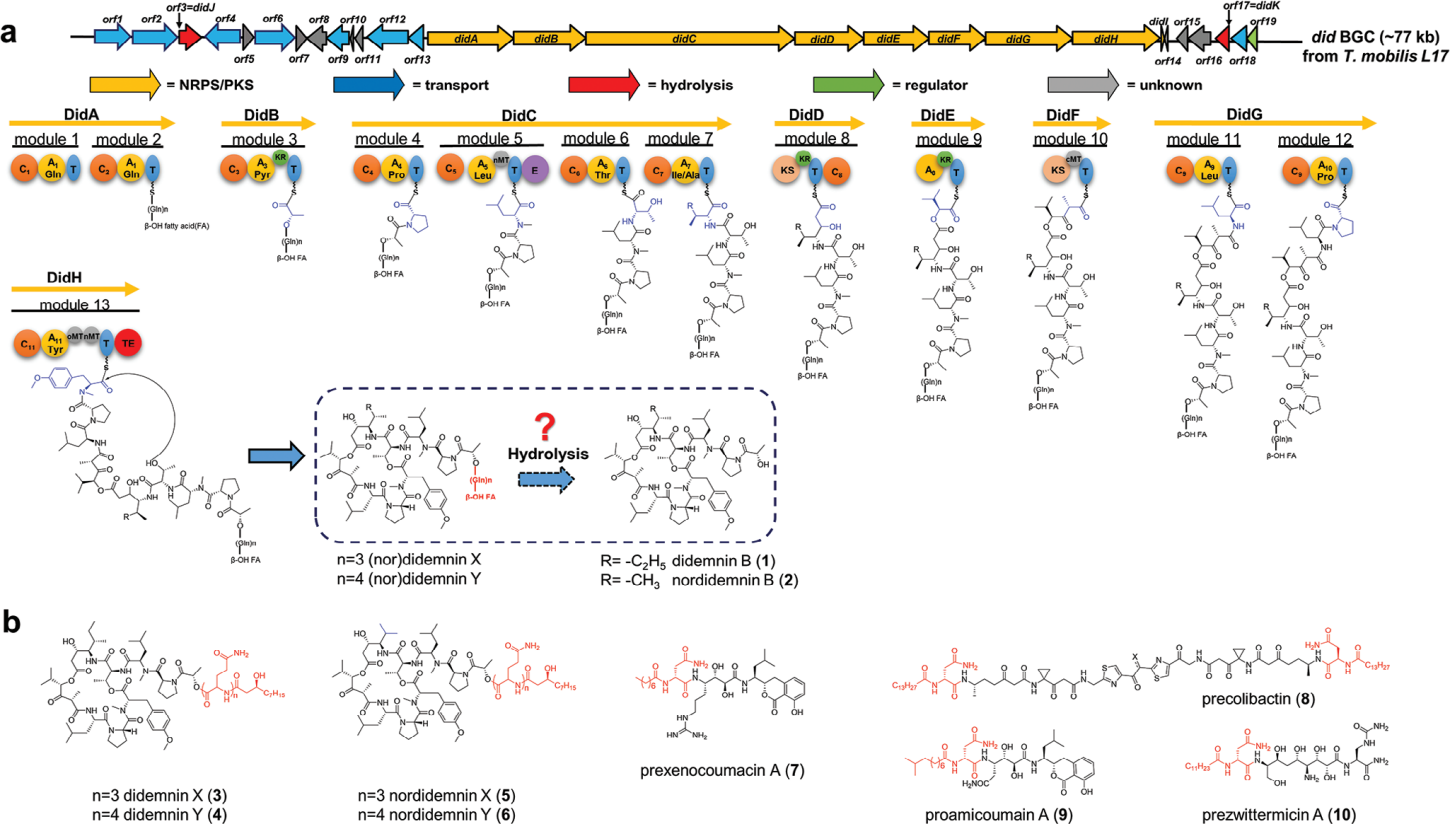
Didemnin biosynthesis is proposed to through a prodrug activation mechanism. a) Gene organization of the didemnin biosynthetic gene cluster in *T. mobilis* L17 and its proposed biosynthetic process, b) representative examples of prodrug molecules biosynthesized by microbial NRPS‐related assembly lines: didemnin X (**3**), didemnin Y (**4**), nordidemnin X (**5**), nordidemnin Y (**6**), prexenocoumacin B (**7**), precolibactin (**8**), lipoamicoumacin A (**9**), and prezwittermicin (**10**). Domain abbreviation: C, condensation; A, adenylation (recognized amino acid included); T, thiolation; MT, methyltransferase; E, epimerase; KS, *β*‐ketoacyl synthase; KR, ketoreductase; TE, thioesterase.

Didemnins are cyclic depsipeptides isolated from numerous marine tunicates, exhibiting exceptional antitumor and antiviral properties.^[^ ^1^ ^]^ Among them, didemnin B (**1**, **Figure** **1**a) has demonstrated the most potent bioactivity against various cancer cell lines, making it the first marine natural product subjected to clinical trials as an anticancer agent. Notably, dehydrodidemnin B (also called plitidepsin), a close analogue of didemnin B featuring a reduced side chain, was successfully approved as a drug for treating multiple myeloma in Australia in 2018. Recently, plitidepsin has gained considerable attention as a potential anti‐COVID‐19 agent in clinical trials.^[^ ^2^ ^]^

While the origin of didemnins in tunicates remains elusive, prior studies have pinpointed two marine *α*‐proteobacteria, namely *Tistrella mobilis* and *T. bauzanensis*,^[^ ^3^ ^]^ are capable of producing primarily didemnin B and other congeners, such as nordidemnin B (**2**, Figure 1a), and the *N*‐acyl‐polyglutamine ester derivatives didemnins X/Y (**3/4**, Figure 1b) and nordidemnins X/Y (**5/6**, Figure 1b). The biosynthesis of didemnins is attributed to a large nonribosomal peptide synthetase and polyketide synthase (NRPS‐PKS) hybrid biosynthetic gene cluster (BGC), which encodes 11 NRPS modules and 2 PKS modules.^[^ ^3b^ ^]^ However, the biosynthetic logic of the proposed assembly line is only consistent with the chemical structures of the *N*‐acyl‐polyglutamine ester derivatives such as didemnins X/Y and nordidemnins X/Y (Figure 1a). Furthermore, imaging mass spectrometry (IMS) of *T*. *mobilis* bacterial colonies facilitated the visualization of a time‐dependent conversion of didemnins X/Y to didemnin B on an agar plate.^[^ ^3b^ ^]^ These observations suggest the immediate product of the NPRS‐PKS assembly line is not didemnin B, but rather the *N*‐acyl‐polyglutamine ester derivatives like didemnins X/Y. This implies the involvement of a post‐cleavage mechanism within the didemnin biosynthesis.

While proteolytic cleavage for peptide maturation is a common feature in the biosynthesis of ribosomally synthesized peptides in bacteria,^[^ ^4^ ^]^ the occurrence of a similar post‐hydrolase activation mechanism for nonribosomally produced peptides is relatively infrequent.^[^ ^5^ ^]^ Bode and colleagues uncovered one of the earliest examples in the biosynthesis of the antibiotic xenocoumacin from the Gram‐negative bacterial species *Xenorhabdus nematophila*, which is in symbiosis with *Steinernema* nematodes.^[^ ^6^ ^]^ They identified that the immediate products from the xenocoumacin assembly line were *N*‐acyl‐d‐asparagine attached prodrug molecules, exemplified by prexenocoumacin B (**7**, Figure 1b). These precursor molecules are then cleaved by a membrane‐bound d‐asparagine–specific peptidase, which is known as XcnG peptidase, to generate xenocoumacin. Another notable example is colibactin, a compound synthesized through a NRPS‐PKS BGC to produce the prodrug molecule precolibactin (**8**, Figure 1b).^[^ ^7^ ^]^ This prodrug is subsequently cleaved by a homolog of XcnG peptidase, named ClbP, releasing the genotoxic molecule colibactin within the human gut.^[^ ^8^ ^]^ Two other antimicrobials, amicoumacin and zwittermicin, also undergo biosynthesis using a similar prodrug mechanism. This mechanism involves the utilization of prodrug molecules known as lipoamicoumacin A (**9**, Figure 1b) and prezwittermicin (**10**, Figure 1b), respectively.^[^ ^5^ ^]^ However, in the process of didemnin biosynthesis, we propose the involvement of a closely related yet distinct mechanism. Firstly, the immediate products synthesized by the didemnin assembly line are presumed to be the *N*‐acyl‐polyglutamine ester derivatives (Figure 1a), suggesting that cleavage might occur at the ester bond rather than the amide bonds observed in other cases. Secondly, the *N*‐terminal modification exhibits more flexibility in didemnin biosynthesis compared to other instances of acyl‐d‐asparagine modified prodrugs, as the *N*‐acyl‐polyglutamine moiety comprises three or four glutamine residues. Collectively, these information point toward the presence of a distinct natural prodrug mechanism in the biosynthesis of didemnin. Here, we describe the elucidation of the natural prodrug mechanism involved in the biosynthesis of didemnins, which is found to be essential for the export of the mature didemnins extracellularly.

## 2 Results and Discussion

### 2.1 DidA Catalyzes the Formation of the N‐Terminal Acyl‐Poly‐Glutamine Moiety in Didemnin Biosynthesis

**Figure 2:**
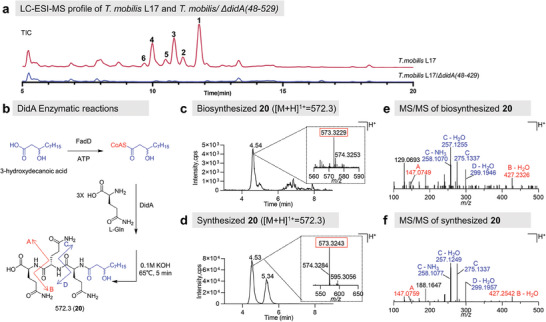
DidA is an NRPS involved in constructing the prodrug scaffold. a) Gene deletion of didA in the didemnin biosynthetic gene cluster in *T. mobilis* L17 lead to the loss of the didemnin production measured by LC‐ESI‐MS, including didemnin B (**1**), nordidemnin X (**2**), didemnin X (**3**), didemnin Y (**4**), nordidemnin X (**5**), nordidemnin Y (**6**); b) reaction scheme for the DidA activity assay; c) extracted ion chromatograms of the biosynthesized product 3‐hydroxydecanoyl‐triglutamine (**20**) obtained from the DidA activity assay; d) high resolution LC‐MS/MS fragmentation of biosynthesized **20**; e) extracted ion chromatograms of the synthesized **20** as a standard; f) high resolution LC‐MS/MS fragmentation of synthesized **20**.

Our exploration into the prodrug mechanism commenced with investigating into the potential of DidA, a bimodular NRPS, as the initiator of didemnin biosynthesis through synthesizing the *N*‐acyl‐polyglutamine moieties. We recently discovered a highly proficient didemnin producer, *T*. *mobilis* L17, capable of yielding over 30 mg L^−1^ didemnin B in an optimized fermentation medium.^[^ ^9^ ^]^ Through whole genome sequencing and bioinformatic analysis, we pinpointed a large NRPS‐PKS BGC on the chromosome of the L17 strain, displaying a striking resemblance (87–100% identity) to the reported *did* BGC. To substantiate the indispensability of DidA in initiating didemnin biosynthesis, we developed a genetic tool‐based on sucrose counterselection (Figure S1 and Table S1, Supporting Information), enabling us to delete a portion of *didA* in *T*. *mobilis* strain (Figure S2, Supporting Information). As anticipated, the *T*. *mobilis* L17/∆*didA(48–429)* mutant strain lost the ability to produce any didemnins (**Figure** **2**a), providing strong evidence that *didA* is essential for didemnin production. To further investigate the function of DidA, we cloned and expressed a codon‐optimized *didA* gene in *E*. *coli* BAP1 strain (Supporting Information). This endeavor yielded a modest quantity of soluble protein, which was subsequently purified in low yields (Figure S3, Supporting Information). Next, we introduced the purified DidA into a reaction mixture containing purified CoA‐ligase from *E*. *coil* (FadD), ATP, racemic 3‐hydroxydecanoic acid, and l‐glutamine (Figure 2b). This approach enabled us to detect the formation of *N*‐acyl‐triglutamine peptide using high resolution LC‐MS/MS (Figure 2c,d), in direct comparison with a synthetic standard (Figure 2e,f; Figures S4 and S28–S32, Supporting Information). Additionally, we were able to observe the production of *N*‐acyl‐tetraglutamine peptide in a scaled‐up reaction via HR‐LCMS/MS (Figure S5, Supporting Information). Collectively, our findings corroborate the role of the DidA bimodular NRPS as the priming synthetase responsible for initiating the didemnin biosynthesis pathway. Since both A domains of DidA prefer to activate glutamine, we therefore posit that the NRPS modules of DidA have the capacity to format fatty acid‐linked polyglutamine intermediates in an iterative behavior.

### 2.2 DidK Is Required for Cleaving Didemnin Prodrugs

**Figure 3:**
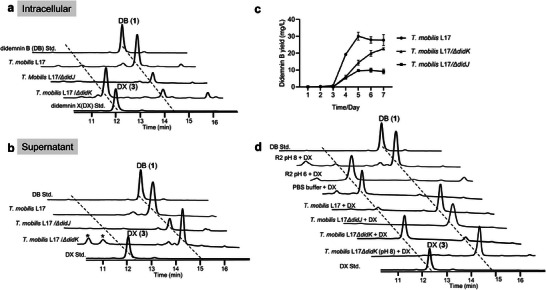
DidK is required for cleaving didemnin prodrugs. a) HPLC analysis of didemnin B (**1**, std.) and didemnin X (**3**, std.) standards and cellular extracts from *Tistrella* cultures, including wild‐type *T. mobilis* L17, *T. mobilis* L17/Δ*didJ*, and *T. mobilis* L17/Δ*didK*; b) HPLC analysis of didemnin B (**1**, std.) and didemnin X (**3**, std.) standards and extracts from supernatant of *Tistrella* cultures, including wild‐type *T. mobilis* L17, *T. mobilis* L17/Δ*didJ*, and *T. mobilis* L17/Δ*didK*; c) the measurement of the production of didemnin B in a 7‐day fermentation process from the cultures of wild‐type *T. mobilis* L17, *T. mobilis* L17/Δ*didJ*, and *T. mobilis* L17/Δ*didK*; d) HPLC analysis the stability of didemnin X (**3**) under different pH and the abilities of the cell lysates of wild‐type *T. mobilis* L17, *T. mobilis* L17/Δ*didJ*, *T. mobilis* L17/Δ*didK*, and *T. mobilis* L17/Δ*didJ* (pH = 8.0) to hydrolyze didemnin X (**3**). * Unresolved peaks in the trace.

Having established the role of DidA in synthesizing the *N*‐acyl‐polyglutamine moieties, we turned toward unraveling the genes responsible for removing these prodrug motifs. We examined 19 open reading frames (ORFs) flanking the core NRPS‐PKS genes. Among them, we identified a regulator protein (in green), two hydrolase analogues (in red), eight transporters (in blue), and eight hypothetical proteins (in gray) (Figure 1a). We selected the two hydrolase candidate genes, *didJ* and *didK*, for gene knockout (Figures S6 and S7, Supporting Information), which encode a putative type II thioesterase (TE) and a CAAX protease homolog, respectively. Our findings unveiled that the *T*. *mobilis* L17/Δ*didJ* mutant retained the ability to produce 1, albeit at a significantly reduced level both intracellularly and extracellularly (**Figure** **3**a,b). In fact, it sustained approximately 30% of the productivity of didemnin B when compared to the wild‐type producer (Figure 3c). Type II TEs, hydrolytic enzymes often cluster with PKS and/or NRPS BGCs and assume crucial roles in maintaining the efficiency of assembly lines. They accomplish this by excluding aberrant residues, selecting accurate substrates, and releasing terminal products.^[^ ^10^ ^]^ Our data strongly supports the designation of DidJ as a type II TE, responsible for preserving the productivity of the didemnin assembly line.

Conversely, the deletion of *didK* resulted in the accumulation of **3** intracellularly (Figure 3a). Surprisingly, we observed no accumulation of **3** in the supernatant of *T*. *mobilis* L17/Δ*didK* (Figure 3b). This prompted us to hypothesize that the *N*‐acyl‐polyglutamine ester moiety of **3** might be undergoing swift hydrolysis, thereby transforming into **1**, particularly under alkaline conditions. While the initial pH of the R2 medium was moderately acidic (pH 6.0), the pH of the culture rose sharply to 8.0 during the 7‐day fermentation (Figure S7, Supporting Information). To investigate the stability of **3** under these pH conditions, we inoculated purified **3** (Figure S8, Supporting Information) in R2 medium at two distinct pH levels (6.0 and 8.0) at 25 °C for 24 h. Remarkably, compound **3** was found to be fully converted to **1** in R2 medium under pH 8.0, while ˂ 5% of **3** converted to **1** in the pH 6.0 R2 medium (Figure 3d). To further substantiate this finding, we introduced purified **3** into buffers covering a pH range from 6.5 to 10.0, providing additional evidence that **3** is not stable in an alkaline environment (Figure S9, Supporting Information). These findings offer a plausible explanation for the lack of accumulation of **3** in the supernatant of *T*. *mobilis* L17/Δ*didK* and lead us to consider the possibility that DidK catalyzes the conversion of **3** to **1**.

To test our hypothesis, we introduced purified **3** into the cell lysates of *T*. *mobilis* L17, *T*. *mobilis* L17/Δ*didJ*, and *T*. *mobilis* L17/Δ*didK*, respectively, while using PBS buffer (pH 7.0) as a control (Figure 3d). After incubation at 25 °C for 24 h, the crude extracts of the reactions were analyzed by HPLC and LC‐MS in comparison with purified standards (Figures S8, S18–S26 and Tables S4–S5, Supporting Information). As expected, both the cell lysates of *T. mobilis* L17 and *T. mobilis* L17/Δ*didJ* showed the ability to fully hydrolyze **3**, yielding **1** (Figure 3d). However, in the case of *T*. *mobilis* L17/Δ*didK*, we only detected a negligible amount of **1** when **3** was introduced into its cell lysate, similar to the control reaction using PBS buffer (Figure 3d). In contrast, upon adjusting the pH of *T*. *mobilis* L17/Δ*didK* lysate to 8.0, compound **3** underwent a rapid conversion to **1** during incubation (Figure 3d). These results strongly support the conclusion that DidK is the sole hydrolase in the *T. mobilis* L17 for removing the *N*‐acyl‐polyglutamine moiety from **3**.

### 2.3 DidK Is a CAAX Protease Homologue with Esterase Activity

Based on a search of the conserved domain in the NCBI database, DidK was annotated as an intramembrane CAAX protease‐like protein (Figure S10, Supporting Information). This protein family is well‐known for its involvement in post‐prenylation modifications of proteins containing a *C*‐terminal CAAX motif (where A is an aliphatic amino acid and X is any amino acid) in eukaryotic cells.^[^ ^11^ ^]^ Upon the attachment of a farnesyl group to the cysteine residue of the CAAX motif, the AAX tripeptide is subsequently removed by CAAX proteases.^[^ ^12^ ^]^ However, the functional roles of this protein family in prokaryotes remain largely unexplored. Interestingly, in certain bacteriocin loci, when genes encoding CAAX hydrolases are found downstream of bacteriocin structural genes, they have been proposed to be associated with self‐immunity.^[^ ^13^ ^]^ In a recent study, Walker and colleagues investigated the SagB‐SpdC hydrolase complex involved in the cell wall biosynthesis of *Staphylococcus aureus*. This complex serves as a peptidoglycan release factor and also shares structural similarities to eukaryotic CAAX proteases.^[^ ^14^ ^]^

Given the established nature of CAAX proteases as membrane proteins, we employed the online tool DeepTMHMM to predict the membrane topology of DidK (Figure S11, Supporting Information).^[^ ^15^ ^]^ The analysis revealed that DidK is a polytopic transmembrane α‐helical protein with eight transmembrane segments (Figure S11, Supporting Information). To further validate this observation, we overexpressed DidK in *E*. *coli* BL21 strain, followed by the isolation of cell membrane‐bound proteins for western blot and activity assays using *p*‐nitrophenyl acetate (**23**) as the substrate (Figure S11, Supporting Information). The use of *p*‐nitrophenyl acetate as a substrate is common in assays for esterase and lipase activity. These results support the notion that DidK is more likely an esterase than a protease.

**Figure 4:**
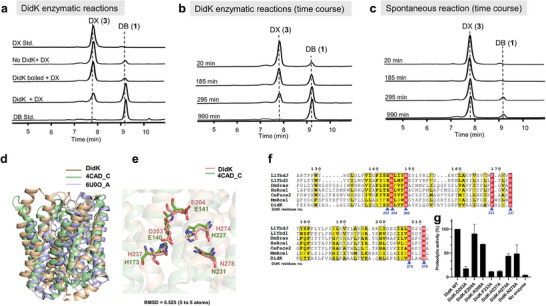
DidK is a bacterial CAAX protease‐like protein with esterase activity. a) HPLC analysis of didemnin B (**1**, std.) and didemnin X (**3**, std.) standards, and the DidK activity assay with no DidK, boiled DidK, and the DidK activity assay for 12 h. b) HPLC analysis of the DidK activity assay in a time course ranging from 20 to 990 min; c) HPLC analysis of the spontaneous hydrolysis of didemnin X in a time course ranging from 20 to 990 min; d) overlap of the predicted structure of DidK with Rce1 (PDB: 4CAD_C, RMSD = 7.552) and SagB‐SpdC hydrolase complex (PDB: 6U0O_A, RMSD = 8.388); e) overlap of the predicted catalytic and conserved residues of DidK with Rce1 (PDB: 4CAD_C). f) multiple sequence alignment of the catalytic domains from DidK homologues representing all three domains of life: LlYbdJ and LlYbdI, from Lactococcus lactis (with UniPort ID Q9CJ66 and Q9CJ67, respectively); DmSras from Drosophila melanogaster (UniPort ID Q9U1H8), HsRce1 from Homo sapiens (UniProt ID Q9Y256), CeFace2 from Caenorhabditis elegans (UniProt ID G5EEP3), and MmRce1 from Methanococcus maripaludis (UniProt ID Q6LZY8). Residues that were mutated in this study are indicated by blue arrows. g) enzymatic activity of wild‐type DidK (DidK WT; set to 100%) and point mutants of DidK toward didemnin X (**3**). The data are presented as the mean +s.d. of *n* = 3 experiments.

We also used AlphaFold^[^ ^16^ ^]^ to predict the 3D structure of DidK. While the overall structure of DidK exhibits great structural difference compared to both the type II CAAX prenyl protease Rce1 (PDB: 4CAD_C, RMSD = 7.552) from the archaea *Methanococcus maripaludis* ^[^ ^11^ ^]^ and the SagB‐SpdC hydrolase complex (PDB: 6U0O_A, RMSD = 8.388) from *Staphylococcus aureus*,^[^ ^14^ ^]^ the predicted catalytic residues of DidK (D203, E204, H237, H274, N278) closely align with the conserved catalytic residues in MmRce1 (E140, E141, H173, H227, N231) (RMSD = 0.525, **Figure** **4**d,e). To confirm DidK's function in vitro, we cloned and expressed the gene for DidK in *E*. *coli*. Previous studies have shown that proteins from this family are challenging to express and purify. After numerous attempts, we found that DidK could only be expressed in a soluble form when tagged with the trigger factor (TF) fusion protein (Figure S12, Supporting Information), which is an chaperone protein that aids in proper folding and prevents misfolding.^[^ ^17^ ^]^ While trying to remove the TF tag from TF‐DidK, we encountered complete precipitation of the untagged protein in the solution. Therefore, we added the purified TF‐DidK protein in a reaction mixture containing **3** as a substrate, resulting in a new peak corresponding to the didemnin B standard (Figure 4a). The formation of the *N*‐acyl‐triglutamine moiety was also observed by LC‐MS/MS analysis in comparison with a synthetic standard (Figure S13, Supporting Information). Given that we observed spontaneous hydrolysis of **3** in both DidK inactivated reaction and PBS buffer, we conducted a time course analysis ranging from 20 to 990 min to compare the formation of **1** in both enzymatic and spontaneous reactions (Figure 4b,c). As expected, in the presence of DidK, compound **3** was completely converted into **1** after overnight inoculation at 37 °C (Figure 4b). Conversely, in the spontaneous reaction, ˂ 5% of **3** was converted into **1** under the same conditions (Figure 4b,c). The Michaelis‐Menten kinetic analysis of TF‐DidK revealed a *K*_m_ value of **3**.443 mm and *V*_max_ of 0.02 684 µmol s^−1^ for **3** (Figure S14, Supporting Information). However, it should be noted that these kinetic parameters may not accurately reflect the actual capability of DidK in the cell membrane. Furthermore, upon introducing purified didemnin Y (**4**) into the DidK assay, we observed a similar conversion as with didemnin X (Figure S15, Supporting Information). Collectively, the genetic and biochemical data provide strong evidence supporting the role of DidK as the sole hydrolase in the didemnin producer, responsible for removing the *N*‐acyl‐polyglutamine moiety from **3** to generate **1**.

Next, we aimed to gain more insights into DidK's catalytic capability by introducing single amino acid variation. These residues were selected based on their significance for catalysis in the analogous MmRec1 enzyme,^[^ ^11^ ^]^ including D203 (E140 in MmRec1), E204 (E141 in MmRec1), and H237 (H173 in MmRec1) (Figure 4f). We also examined several conserved residues, including R208, F233, H274, and N278, which are shared among all CAAX proteases (Figure 4f; Figure S10, Supporting Information). In MmRec1, mutations of E140, E141, and H173 were found to impair its enzymatic activity substantially. Notably, both E141 and H173 are believed to play a role in coordinating a bridging water molecule, which is crucial for nucleophilic attack on the substrate's amide bond.^[^ ^11^ ^]^ In the case of DidK, we identified D203 (corresponding to E140 in MmRec1) and H237 (corresponding to H173 in MmRec1) as essential for maintaining activity. Furthermore, replacing either H274 or H278 with an alanine residue can reduce over half of DidK's catalytic ability (Figure 4g). Since these residues are structurally predicted to closely align with the corresponding catalytic residues in MmRce1, we propose that DidK may employ a similar catalytic mechanism as MmRce1. Specifically, both D203 and H237 are likely involved in priming a water molecule to facilitate its nucleophilic attack on the scissile bond of the substrate (Figure S16, Supporting Information). Simultaneously, the side chains of H274 and N278 may play a role in stabilizing the oxyanion transition state of the substrate. However, unlike other reported CAAX proteins that prefer to hydrolyze amide bonds, DidK appears to prefer hydrolyzing ester bonds. To provide a comprehensive explanation for these outcomes, we are still actively pursuing the crystal structure of DidK.

While we were in the process of revising this paper, the Wenjun Zhang lab published a study in which they elucidated the function of DidK by conducting gene knockout experiments and analyzing DidK's activity in cellular crude extracts. In the study, they also identified that substituting either E201 (corresponding to E203 in our paper) or H235 (corresponding to H237 in our paper) with an alanine residue substantially reduced DidK's catalytic ability.^[^ ^18^ ^]^ Our findings, in conjunction with theirs, underscore the pivotal importance of these two residues in maintaining DidK's activity.

### 2.4 DidK Is Essential for the Secretion of Didemnin B on Agar Plate

**Figure 5:**
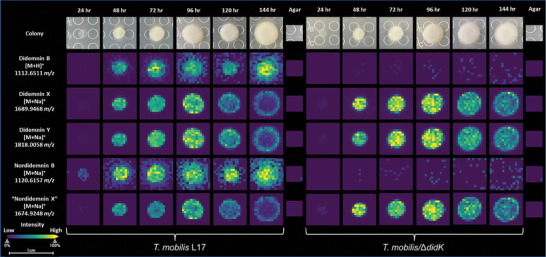
Time‐course production of didemnins by *T. mobilis* L17 and *T. mobilis* L17/ΔdidK visualized by MALDI‐IMS.

The natural prodrug activation of NRPS and NRPS‐PKS products serves as a mechanism to prevent self‐destruction in the producing strain and/or facilitate the export of biosynthesized natural products.^[^ ^5^ ^]^ In the context of didemins, these compounds have no demonstrated toxicity toward the producers. Therefore, we investigated whether DidK is involved in the process of didemnin exportation by monitoring the temporal and spatial distribution of the prodrug molecules (didemnin X/Y and nordidemnin X) and mature didemnins (didemnin B and nordiemnin B) using MALDI‐IMS on agar plates. We refrained from measuring the production of nordidemnin Y due to its exceedingly low yield. We studied both the *T*. *mobilis* L17 wild‐type strain and the *T*. *mobilis* L17/Δ*didK* mutant colonies over a time course ranging from 24 to 144 h (**Figure** **5**). Remarkably, our findings revealed that the prodrug molecules, like didemnins X/Y and nordidemnin X, are exclusively produced intracellularly in both strains on the agar plates. Conversely, didemnin B and nordidemnin B were exported to the agar plate from the *T*. *mobilis* wild‐type strain (Figure 5). However, a stark contrast emerged in the case of the *T*. *mobilis* L17/Δ*didK* mutant colonies, where only trace amount of didemnin B and nordidemnin B were detectable throughout the entire time course (Figure 5). These results support the notion that DidK is critical in hydrolyzing the prodrug scaffolds of didemnin. Such enzymatic activity is instrumental in liberating the mature molecules, permitting their release into the extracellular milieu.

## 3 Conclusion

In summary, our genetic and biochemical investigations have established the involvement of the first two NRPS modules in the didemnin assembly line, driving the initiation of didemnin biosynthesis through the formation of *N*‐acyl‐polyglutamine ester moieties. Additionally, we have successfully identified and characterized the membrane‐bound CAAX hydrolase‐like protein, designated as DidK. This enzyme is responsible for catalyzing the hydrolysis of the ester bond, a process crucial for the maturation of didemnins. Employing IMS on *T. mobilis* bacterial colonies, we have directly visualized that this hydrolysis event is indispensable for the extracellular export of the mature didemnins. This discovery not only enriches the current understanding of post‐assembly activation mechanisms in NRPS‐PKS‐derived natural products but also unveils novel insights into the functions of CAAX hydrolases in microbes. Given that the combination of didK homologs and NRPS/PKS BGCs exists is present in other bacterial genomes (Figure S17, Supporting Information), it is plausible that this knowledge has the potential to guide the strategic design of genome mining strategies aimed at the exploration of novel bioactive natural products utilizing analogous prodrug mechanisms.

## 4 Experimental Section

### Bacterial Strains and Growth Conditions

*T*. *mobilis* strains were retrieved from −80 °C refrigerator, and 10 µL of the suspension was spread onto Luria‐Bertani plates (LB, containing 10.0 g of NaCl, 10.0 g of tryptone, 5.0 g of yeast extract, and 12.0 g of agar, in 1.0 L dd water) containing 50 µg mL^−1^ ampicillin. After a 2‐day incubation at 25 °C, 3 or 4 distinct large colonies were selected and cultured in a seed medium (1.0 g of galactose, 5.0 g of glycerol, 3.0 g of yeast extract, 5.0 g of peptone, in 1.0 L dd water). The OD600 of the seed culture reached ≈0.6–0.8 after a 2‐day inoculation. The seed culture was then inoculated into 500 mL R2 meduium (4.0 g of galactose, 20.0 g of glycerol, 12.0 g of yeast extract, 20.0 g of peptone, in 1.0 L ddH_2_O) at a 1:100 ratio. The fermentation was incubated for 7 days at 25 °C and 220 rpm. Samples of the fermentation were collected every 24 h for pH, OD_600_, and didemnin B (1) and didemnin X (**3**) yield measurements. The collected samples were separated into bacterial cells and supernatant. The bacterial cells were disrupted with an equal volume of 0.2 mm glass beads, followed by centrifugation (10 min, 14 000 rpm) to obtain the supernatant. The supernatant was then extracted with an equal volume of ethyl acetate. The organic phase was concentrated under vacuum, and the resulting residue was dissolved in 100 µl of CH_3_CN. After centrifugation (30 min, 14 800 rpm), the supernatant was subjected to LC‐MS analysis.

For *E*. *coli* strains, growth was carried out on LB plates or in LB liquid medium at 37 °C with appropriate antibiotics for selection (ampicillin 50 µg mL^−1^, kanamycin 50 µg mL^−1^). For protein production, *E*. *coli* strains were grown in TB medium (12.0 g of tryptone, 24.0 g of yeast extract, 4.0 g of glycerol, 23.1 g of KH_2_PO_4_ and 12.54 g of K_2_HPO_4_, in 1.0 L ddH_2_O) at 25 °C with appropriate antibiotics.

### Construction of the Sacb‐Based Vector Plasmid pTHZ001

A 1697 bp fragment containing a heat sensitive origin of replication (*Rep101(Ts)‐pSC101*) was amplified from the plasmid pJZ002^[^ ^19^ ^]^ with the primer pairs JZ002_F/R (Table S1, Supporting Information). The gentamycin resistance gene (*Gm* ^R^) was amplified from pJZ001^[^ ^19^ ^]^ with the primer pairs JZ001_F/R (Table S1, Supporting Information). A 2323 bp fragment containing the genes responsible for conjugation and antibiotic resistance (*oriT‐traJ‐KanR*) was amplified from the plasmid pCAP01^[^ ^20^ ^]^ with the primer pairs pCAP01_F/R (Table S1, Supporting Information). The *sacB* gene^[^ ^21^ ^]^ (Supporting Information), which encodes protein conferring sucrose sensitivity, was synthesized by Sangon Biotech (Shanghai, China). These four DNA products were assembled by Gibson assembly. After transforming into *E*. *coli* DH5α, the positive clones were harvested from LB agar plates (containing 50 µg L^−1^ gentamycin) and the isolated plasmid pTHZ000 (Table S2, Supporting Information) was verified by restriction enzyme digestion. To facility later cloning, an annealed oligonucleotide fragment was added, which contains a multiple cloning site (MCS), in between ApaLI and ScaI cut sites to give pTHZ001 (Table S2, Supporting Information). The plasmid pTHZ001 was verified by restriction enzyme digestion and sequencing.

### Construction of the *T. mobilis* L17 Knock out Mutants

To partial delete *didA* in *T. mobilis* L17, the first amplified ≈1500 bp homology arms from the upstream and downstream of the targeted region of *did A* (from base pair number 48 to 429) using the primer pairs tmdidA_LF/tmdidA_LR and tmdidA_RF/tmdidA_RR (Table S1, Supporting Information), respectively. The amplified upstream and downstream homologous fragments were then digested by the restriction enzyme combinations ApalI/HindIII and HindIII/XbaI, respectively. In the meantime, the plasmid pTHZ001 was treated with the restriction enzymes ApalI and XbaI. All three DNA fragments were ligated by T4 ligase to give pTZ001::*didA(48–429)* (Table S2, Supporting Information). The pTZ001::*didA(48–429)* plasmid was transferred into the conjugation donor strain *E. coli* S17‐1. The conjugation procedure was processed by biparental mating, the donor and recipient strains (equivalently with adjusted OD_600_ of 5.0) were mixed together after overnight culture, and then 400 µL of the mixture was plated on a LB agar plate (containing extra 20 mm MgCl_2_). After culturing 18–20 h at 30 °C, the plates were overlaid with 1 mL of water containing 500 µg ampicillin (to selectively kill *E. coli*) and 500 µg gentamycin (for selection of mutants). The plate was then incubated for a further 5–7 days at 30 °C, and exconjugants (single‐crossover strains) were further verified by PCR screening and transferred to LB liquid with both antibiotics. The diluted over‐night culture was plated on a LB agar plate containing 5% sucrose w/v and 50 µg mL^−1^ ampicillin. Double‐crossover strains were selected by colony PCR and further confirmed by sequencing. The success rates were observed from 5% to 15% depending on the length of the homologous arms and the sizes of the targeted region. The *T. mobilis* L17/∆*didJ(69–300)* and *T. mobilis* L17/∆*didK(1–1023)* mutant were generated as the same method described in *T. mobilis* L17/∆*didA(48–429)*. Primers and plasmids used for generating these mutants are listed in Tables S1 and S3 (Supporting Information), respectively.

### Purification of Didemnin B (**1**), Nordidemnin B (**2**), Didemnin X (**3**), and Didemnin Y (**4**)

Wild‐type *T. mobilis* L17 and *T. mobilis* L17/∆*didK(1‐1023)* strains were used for the purification of didemnin B (**1**)/nordidemnin B (**2**) and didemnin X (**3**)/didemnin Y (**4**), respectively. Each strain was cultivated in R2 medium for 5 days at 25°C and 220 rpm. After fermentation, the cell pellets and supernatant were separated by centrifugation (20 min, 4500 rpm). The cell pellets were resuspended in a mixture of 50% acetonitrile (CH_3_CN or ACN) and 50% ddH_2_O. The suspension underwent disruption through ultrasonication (80% amplitude, 40 min, pulse 3 s, pause 6 sec), followed by centrifugation (10 min, 14 000 rpm) to collect the resulting supernatant. This supernatant was combined with the previously reserved supernatant, and the mixture was subsequently subjected to ethyl acetate extraction. Subsequently, the organic phase underwent evaporation to concentrate the solution. The resulting residue was dissolved in CH_3_CN, subjected to centrifugation, and then filtered through a 0.22‐µm filter before undergoing further isolation on a semi‐preparative HPLC system (Agilent 1260 Infinity II semi‐preparative HPLC) equipped with a Phenomenex Kinetex XB‐C18 HPLC column (250 × 10 mm, 5 µm) operating at a flow rate of 2.8 mL min^−1^. The elution solvent system consisted of water (A) and acetonitrile (B), with a linear gradient elution starting from 40% v/v solvent B and reaching 95% v/v solvent B over 12 min. This concentration was maintained for 3 min, followed by equilibration at 40% v/v solvent B for an additional 3 min. Subsequently, the samples were collected in round‐bottom flasks and placed in a −80 °C refrigerator for ≈1 h. After freeze‐drying, the samples were transferred to glass bottles for storage.

### Testing the Abilities of the Cell Lysates of *T. mobilis* L17 and its Mutants in Hydrolyzing Didemnin X (**3**)

*T. mobilis* L17, *T. mobilis* L17/ΔdidJ, and *T. mobilis* L17/ΔdidK strains were cultured in LB liquid medium at 25 °C and 220 rpm for a duration of 4 days. The cells were subsequently harvested via centrifugation (20 min, 4500 rpm). Equally weighted samples of these cells were then taken and resuspended in PBS buffer (pH 7.5). The resulting suspension underwent ultrasonication (40% amplitude, 15 min, pulse 3 s, pause 6 s) after which centrifugation was performed (30 min, 14 000 rpm) to collect the lysate. Purified compound **3** (3 mm) was introduced into equivalent amounts of cell lysates from *T. mobilis* L17, *T. mobilis* L17/ΔdidJ, *T. mobilis* L17/ΔdidK, and *T. mobilis* L17/ΔdidK (with adjusted pH 8.0), respectively. PBS buffer (pH 7.5) was used as the negative control. Following an incubation period of 24 h at 25 °C, the crude extracts generated from these reactions were analyzed using HPLC, and the obtained results were subsequently compared to purified standards.

### Construction of the Plasmids for Site‐Directed Mutation of DidK

To construct plasmids for introducing site‐directed mutation into DidK, a fragment with mutant sequence (the mutation was introduced by a pair of primers D203A‐FWD and D203A‐REV) was amplified from *Tistrella mobilis* L17 genome DNA. The PCR products were inserted into pCold‐TF vector by Gibson assembly, resulting in pXZ_D203A. Plasmids pXZ_E204A, pXZ_R208A, pXZ_H233A, pXZ_H237A, pXZ_N278A, each designed for site‐directed mutation of DidK, were constructed using the following primer pairs: E204A‐FWD/E204A‐REV, R208A‐FWD/R208A‐REV, H233A‐FWD/H233A‐REV, H237A‐FWD/H237A‐REV, and N278A‐FWD/N278A‐REV, respectively. These constructed plasmids were then transformed into the *E. coli* BL21(DE3), and the positive clones were selected using the antibiotic ampicillin. Plasmid DNA was isolated using an OMEGA Spin Miniprep kit (OMEGA, USA). The constructed plasmids were confirmed by restriction enzyme digestion and verified via Sanger sequencing (Shanghai, China).

### Protein Overexpression and Purification

The code optimized *didA* gene was custom‐synthesized and inserted into the vector pET‐28a(+) by Sangon Biotech (Shanghai, China). This insertion places the cloned gene under the control of the *T*_7_ promoter and an added *N*‐terminal His_6_‐tag, both of which can be removed via rhinovirus 3C protease cleavage. To facilitate protein expression, the plasmids containing the inserted sequence were introduced into *E. coli* BAP1. Recombinant *E. coli* BAP1 strains were cultivated on LB medium supplemented with 50 µg mL^−1^ kanamycin. For protein expression and purification, a 10 mL LB medium culture with the same supplement was initiated from a single colony and grown at 37 °C and 220 rpm for 6 h. Subsequently, 5 mL of the pre‐culture was inoculated into 500 mL of LB medium with kanamycin (50 µg mL^−1^) and incubated at 37 °C and 220 rpm until the OD_600_ reached a range of 0.6–0.8. Protein expression was induced by adding 0.25 mm IPTG, followed by incubating the culture at 16°C and 160 rpm for 16 h. The cells were harvested by centrifugation (20 min, 4500 rpm) and resuspended in 50 mm Tris‐HCl buffer (pH 7.5) containing 500 mm NaCl, 20 mm imidazole, and 10% v/v glycerol. The cell suspension was disrupted by ultrasonication (40% amplitude, 40 min, pulse 3 s, pause 6 s). After centrifugation (30 min, 18 000 rpm), the supernatant was subjected to column chromatography.

The DidK‐encoding gene from *T. mobilis L17* genomic DNA was PCR‐amplified and introduced into the pCold‐TF vector using a Gibson assembly kit (NEB, USA). In this vector, the cloned gene was placed under the control of the *cspA* promoter, with *C*‐terminal trigger factor and an attached His_6_‐tag. These tags can be cleaved using rhinovirus 3C protease. For the purpose of protein expression, the plasmid carrying the inserted sequence was introduced into *E. coli* BL21(DE3). Recombinant *E. coli* BL21(DE3) strains were cultured on LB medium supplemented with 50 µg mL^−1^ ampicillin. A single colony was inoculated into 10 mL LB medium with the same supplement and grown at 37 °C and 220 rpm for 6 h. Subsequently, 5 mL of the pre‐culture was inoculated into 500 mL of TB medium with ampicillin (50 µg mL^−1^) and incubated at 37 °C and 220 rpm until the OD600 reached a range of 0.6–0.8. Protein expression was induced using 0.5 mm IPTG, and the culture was then incubated at 16 °C and 160 rpm for 16 h. The cells were harvested by centrifugation (20 min, 4500 rpm) and resuspended in 50 mm Tris‐HCl buffer (pH 7.5) containing 500 mm NaCl, 20 mm imidazole, and 10% v/v glycerol. The suspension was disrupted by ultrasonication (40% amplitude, 40 min, pulse 3 s, pause 6 s). After centrifugation (30 min, 18 000 rpm), the supernatant was subjected to column chromatography.

Protein purification was carried out using an ÄKTA purifier instrument (Cytiva, USA) equipped with the Box‐900, UPC‐900, R‐900, and Frac‐900 modules. Prior to use, all buffers were filtered through a 0.22‐µm nylon membrane (Merck, Germany). Fast protein LC data were analyzed with UNICORN 5.31 (Built 743) software. Both DidA and DidK were initially purified by Ni^2+^ affinity chromatography using 5‐mL HisTrap FF (Cytiva, USA) columns pre‐equilibrated with buffer A (500 m NaCl, 50 mm Tris‐HCl, 20 mm imidazole and 0.8 mm DTT,10% glycerol, pH 7.5). Supernatant was loaded onto columns at 1.0 mL min^−1^, followed by washing the column with six column volumes (30 mL) of buffer A. Elution of the protein was achieved by applying a linear gradient from 5% to 100% buffer B (500 m NaCl, 50 mm Tris‐HCl, 500 mm imidazole and 0.8 mm DTT, 10% glycerol, pH 7.5) over 100 min at a flow rate of 1.25 mL min^−1^. Fractions containing the protein of interest were identified by SDS–PAGE (10% acrylamide) in the presence of reducing agents . After combining the activity fractions, they were concentrated to a volume of ∼3 mL using Amicon Ultra centrifugal filters with a 100‐kDa (30‐kDa for DidK) molecular weight cutoff (EMD Millipore, USA). The concentrated protein was subsequently loaded onto a HiLoad Superdex 200 prep‐grade size‐exclusion column (16 × 60 cm, GE Healthcare, USA) equilibrated in a buffer containing 50 mm Tris, 150 mm NaCl, 0.8 mm TCEP, and 10% glycerol (pH 7.5). Elution was performed at a constant flow rate of 1.0 mL min^−1^. Fractions containing the target protein were pooled, concentrated, aliquoted, and then rapidly frozen for storage at −80 °C. Protein concentration was determined using the Bradford assay.

### In Vitro Enzyme Assays

For the DidA enzyme assays, a total volume of 100 µL was utilized, comprising 5 mm FadD (CoA ligase), 5 mm ATP, 5 mm DTT, 10 mm MgCl_2_, 3 mm CoA, 9 mm l‐glutamine, and 3 mm 3‐hydroxydecanoic acid, in addition to 10 µm DidA. These components were suspended in potassium phosphate buffer (pH 7.5). After adding DidA, the solution was maintained at 30 °C for a duration of 15 h. The reaction mixtures were quenched by the adding 10% trichloroacetic acid (TCA). Following centrifugation, the precipitated proteins were pelleted and separated from the supernatant. To dissolve the protein pellet, 100 µL of KOH (0.1 m) was added, and the mixture was heated to 65 °C for 5 min. Additionally, 5 µL of 50% trifluoroacetic acid (TFA) was introduced to the reaction mixture to reprecipitate the proteins. The supernatant was separated by centrifugation and further subjected to LC‐MS analysis.

For Q‐TOF‐HRMS analysis, a 5.0‐µL sample was injected into a Poroshell HPH‐C18 column (2.1 × 100 mm, 2.7 µm) equipped with an Agilent HPH‐C18 safeguard (4.6 × 5 mm, 2.7 µm). The analysis was conducted using a SCIEX TripleTOF 6600 Quadrupole Time‐of‐Flight Mass Spectrometer coupled to a UPLC system. The analysis employed a gradient elution method with a mobile phase consisting of A (H_2_O with 0.1% formic acid (FA)) and B (ACN with 0.1% FA). The gradient profile started with 20% B and increased to 100% B over 13 min, followed by holding at 100% B from 13 to 16 min, then returning to 20% B for 4 min, all at a flow rate of 0.4 mL min^−1^. The Q‐TOF MS settings during the LC gradient were as follows: acquisition in the mass range of m/z 100–2000, with an MS scan rate of 1.25 s^−1^ and an MS/MS scan rate of 1.25 s^−1^, using fixed collision energy of 30 eV. The source settings were as follows: gas temperature of 500 °C, Curtain Gas set to 35, Ion Spray Voltage Floating set to 5500, Ion Source Gas 1 set to 50, Ion Source Gas 2 set to 50, and ion polarity set to positive. Prior to each measurement, the MS was auto‐tuned using SCIEX tuning solution in positive mode. Data acquisition and analysis were performed using the Analyst software (SCIEX, USA).

The enzyme assays for DidK (50 µL) were composed of a mixture of PBS buffer (50 mm, pH 7.5), compound **3** (6 mm), DidK or DidK mutants (100 µm), DMSO (0.2% to 1%). These enzyme assays were incubated at 37 °C for durations ranging from 0 to 1000 min. After incubation, the reactions were quenched by adding an equal volume of cold CH_3_CN and then extracted twice with an equal volume of EtOAc. The organic phase was evaporated, and the resulting extracts were dissolved in CH_3_CN (100 µL). Afterward, they were centrifuged at 13 000 rpm for 30 min before being submitted for UPLC analysis. These enzyme assays were independently repeated to ensure accuracy. For analysis, 5 µL samples were subjected to an Agilent UPLC system (Agilent 1290 Infinity II, USA) equipped with a Phenomenex Kinetex XB‐C18 HPLC column (100 Å, 100 × 4.6 mm, 2.6 µm) at a flow rate of 0.3 mL min^−1^. The chromatographic solvents used were composed of water (A) and acetonitrile (B), both containing 0.1% v/v FA. The chromatographic process initiated with a linear gradient elution starting from 40% v/v solvent B and increasing to 90% v/v B over 13 min. This concentration was maintained at 90% v/v solvent B for 3 min, followed by an equilibration phase at 20% v/v solvent B for 4 min.

### Sample Preparation for Didemnin Production in *T. mobilis* by MALDI Imaging MS

Each *T. mobilis* strain was streaked from frozen stock onto LB agar (1.5% Bacto agar) and incubated for 72 h at 25 °C. A single colony of each strain was inoculated into 5 mL of liquid LB media. The liquid cultures were allowed to shake at 220 pm at 25 °C for 48 h. After incubation, the cultures were normalized to an OD_600_ of 0.1. This normalized culture was spotted (1 µL) in duplicate onto 2 mm thick LB agar plates (1.5% Bacto agar) and allowed to grow for 24 h to 6 days. Separate successive inoculations were performed to generate the colonies for each time point. Colonies at different time points were transferred to Bruker MSP 96 target ground steel plates prior to matrix application.^[^ ^22^ ^]^ The first plate contained the 24‐h to 3‐day timepoints for strains *T. mobilis* L17/*ΔdidK* and *T. mobilis* L17 while the second plate contained the 4 to 6‐day timepoints for *T. mobilis* L17/Δ*didK* and *T. mobilis* L17. A picture was taken before matrix application for direct comparison with the MALDI‐imaged colonies. A thin layer of matrix [1:1 ɑ‐cyano‐4‐hydroxycinnamic acid (CHCA): 2,5‐dihydrobenzoic acid (DHB)] was applied using a 53 µm sieve (Hogentogler & Co, USA). The plates were then dried for 1 h in an incubator at 35 °C using a homemade spinning apparatus (citation below). The plates were removed from the incubator to apply a second layer of matrix before being allowed to dry for an additional hour at 35 °C. Another picture of each MALDI target plate was taken post‐desiccation to establish each point for MALDI imaging. The samples were then analyzed on a Bruker timsTOF fleX mass spectrometer.

Observation of didemnin production in *T. mobilis* L17 by MALDI Imaging MS Imaging mass spectrometry data was acquired using timsControl v4.1.12_8bfb6bd_1 and flexImaging 7.2 software at 500 µm spatial resolution on a timsTOF fleX instrument (Bruker Daltonics, Billerica, MA, USA). The data were collected using the mass range 50–2000 Da in positive mode with laser power set to 45%, and laser width to 500 µm imaging. For each raster point 300 laser shots at 1000 Hz were shot. Data was subsequently analyzed in SCiLS Lab version 2023b core (Bruker Daltonics, Billerica, MA, USA). The instrument was calibrated manually using phosphorus red.^[^ ^22^ ^]^

## Conflict of Interest

The authors declare no conflict of interest.

## Supporting information

Supporting Information

## Data Availability

The data that support the findings of this study are available in the supplementary material of this article.
