# Supplementary material for: Unveiling a CAAX Protease‐Like Protein Involved in Didemnin Drug Maturation and Secretion
Source: Adv Sci (Weinh). 2023 Nov 30;11(4):2306044. doi: 10.1002/advs.202306044 (PMC10811503; doi:10.1002/advs.202306044)
Supplement: Supplementary file 1 — Supporting Information [file ADVS-11-2306044-s001.pdf]

ADVANCED SCIENCE
Open Access

Supporting Information

for *Adv. Sci.*, DOI 10.1002/advs.202306044

Unveiling a CAAX Protease-Like Protein Involved in Didemnin Drug Maturation and Secretion

*Xiaolin Zou, Zhen Hui, Robert A. Shepherd, Shuaiqiang Zhao, Yanfei Wu, Zhuanglin Shen, Cuiping Pang, Shipeng Zhou, Zehai Yu, Jiahai Zhou, Bradly S. Moore, Laura M. Sanchez and Xiaoyu Tang**

Supporting Information

Unveiling a CAAX protease-like protein involved in didemnin drug maturation and secretion

Xiaolin Zou+[a], Zhen Hui+[a], Robert A. Shepherd[b], Shuaiqiang Zhao[a], Yanfei Wu[c], Zhuanglin Shen[c], Cuiping Pang[c], Shipeng Zhou[a], Zehai Yu[a], Jiahai Zhou[c], Bradly S. Moore[d,e], Laura M. Sanchez[b], and Xiaoyu Tang*[a]

[a] X. Zou, Z. Hui, S. Zhao, S. Zhou, Z. Yu, Prof. Dr. X. Tang

Institute of Chemical Biology, Shenzhen Bay Laboratory, 518132, Shenzhen, China

*Email: xtang@szbl.ac.cn or xtang@microbechembio.org

[b] R. A. Shepherd, Prof. Dr. L. M. Sanchez

Department of Chemistry and Biochemistry, University of California Santa Cruz, 1156 High Street, Santa Cruz, CA 95064, USA

[c] Y. Wu, Dr. Z. Shen, Dr. C. Pang, Prof. Dr. J. Zhou

CAS Key Laboratory of Quantitative Engineering Biology, Shenzhen Institute of Synthetic Biology, Shenzhen Institute of Advanced Technology, Chinese Academy of Sciences, Shenzhen, 518055, China

[d] Prof. Dr. B. S. Moore

Scripps Institution of Oceanography, University of California, San Diego, La Jolla, CA 92093, USA

[e] Prof. Dr. B. S. Moore

Skaggs School of Pharmacy and Pharmaceutical Sciences, University of California, San Diego, La Jolla, CA 92093, USA

[+] These authors contributed equally to this work.

Contents

Materials and Methods 4
1. DNA isolation and manipulation 4
2. DNA and protein sequences used in this study 4
3. Chemical synthesis of compounds 12-22 8
4. Bioinformatic Analysis. 12
5. Western bolt analyses 12
6. General methods 13
Supplementary tables 14
Supplementary table S1. List of primers used in this study 14
Supplementary table S2. Plasmids used in this study 15
Supplementary table S3. Strains used in this study. 16
Supplementary table S4. ^{1}H NMR (400 MHz, $CDCl_3$) and ^{13}C NMR data for didemnin B (1, $CDCl_3$) and nordidemnin B (2, $CDCl_3$). 17
Supplementary table S5. ^{1}H NMR and ^{13}C NMR data for didemnin X (3, $CDCl_3$). 19
Supplementary figures 21
Figure S1. Plasmid map of pTHZ001. 21
Figure S2. Verification of *T. mobilis/ΔdidA*(48-429) mutant by Sanger sequencing. 22
Figure S3. SDS-PAGE analysis of the expression and purification of DidA 23
Figure S4. Chemical synthesis of compounds 20. 25
Figure S5. Biochemical reconstitution of the biosynthesis of *N*-acyl- tetraglutamine peptide (23) by purified Did A. 27
Figure S6. Verification of *T. mobilis/ΔdidJ* (69-300) and *T. mobilis/ΔdidK* (1-1023) mutants by Sanger sequencing. 28
Figure S7. Verification of the knockout strains *T. mobilis* L17/*ΔdidJ* and *T. mobilis* L17/*ΔdidK*. 29
Figure S8. Isolation of didemnin B (1) and didemnin X (3) 31
Figure S9. The stability of didemnin X in reaction buffer across a pH range from pH 6.5 to 10. 34
Figure S10. Alignment of DidK (top) and some representative homologs 35
Figure S11. DidK is an intramembrane protein with predicted eight transmembrane *α*-helices. 36
Figure S12. SDS-PAGE analysis of the purification of DidK. 37
Figure S13. LC-HRMS/MS analysis of the fragments of the hydrolysate (20) from the reaction of DidK when using didemnin X as the substrate. 38
Figure S14. Determination of the optimum reaction temperature, pH value, the maximum substrate concentration, and Michaelis–Menten kinetics for DidK. 39
Figure S15. DidK is required for cleaving didemnin Y. 40
Figure S16. A plausible catalytic mechanism of DidK. 41
Figure S17. Distribution of DidK homologues in various bacterial genomes 42
Figure S18. ^{1}H NMR spectrum of purified didemnin B (1, 400 MHz). Solvent: $CDCl_3$ 43
Figure S19. ^{13}C NMR spectrum of purified didemnin B (1, 100 MHz). Solvent: $CDCl_3$ 44
Figure S20. ^{1}H-^{13}C-HSQC spectrum of purified didemnin B (1). Solvent: $CDCl_3$. 45
Figure S21. ^{1}H-^{13}C-HMBC spectrum of purified didemnin B (1). Solvent: $CDCl_3$. 46
Figure S22. ^{1}H NMR spectrum of purified nordidemnin B (2, 400 MHz). Solvent: $CDCl_3$ 47
Figure S23. ^{1}H NMR spectrum of purified didemnin X (3, 400 MHz). Solvent: $CDCl_3$ 48
Figure S24. ^{13}C NMR spectrum of purified didemnin X (3), 101 MHz. Solvent: $CDCl_3$ 49
Figure S25. ^{1}H-^{13}C-HSQC spectrum of purified didemnin X (3). Solvent: $CDCl_3$. 50

Figure S26. ^{1}H-^{13}C-HMBC spectrum of purified didemnin X (3). Solvent: $CDCl_3$. 51
Figure S27. ^{1}H NMR spectrum of purified didemnin Y (4, 600 MHz). Solvent: DMSO-*d6* 52
Figure S28. ^{1}H NMR spectrum of compound 19 (600 MHz). Solvent: DMSO-*d6* 53
Figure S29. ^{13}C NMR spectrum of compound 19 (600 MHz). Solvent: DMSO-*d6* 54
Figure S30. ^{1}H NMR spectrum of compound 20 (600 MHz). Solvent: DMSO-*d6* 55
Figure S31. ^{13}C NMR spectrum of compound 20 (600 MHz). Solvent: DMSO-*d6* 56
Figure S32. ^{1}H NMR spectrum of compound 22 (600 MHz). Solvent: DMSO-*d6* 57
References 58

Materials and Methods

1. DNA isolation and manipulation

The isolation of genomic DNA (*g*DNA) from *T. mobilis* was carried out using a established protocol from the TAKARA 9765 Universal Genomic Extraction kit (Takara Bio, Japan). The amplification of genes from the *did* cluster was accomplished using the isolated *g*DNA as a template and a Phusion High-Fidelity DNA Polymerase kit with the provided GC buffer (Takara Bio, Japan). PCR reactions were conducted in a Bio-Rad MyCycler (Bio-Rad, USA). To purify PCR products, both Qiagen QIAquick kit and Gel Cleanup kit were employed in accordance with the manufacturer's instructions (Qiagen, Germany). In preparation for cloning, vector pET-28a(+) was linearized using NcoI and XhoI restriction enzymes, while vector pCold-TF was digested using the restriction enzymes NdeI and XbaI. Gibson assembly, facilitated by NEB HiFi DNA Assembly Master Mix (NEB, USA), was then used to combine the linearized vector with purified PCR products. This composite was subsequently transformed into chemically competent *E. coli* DH5α cells for cloning purposes. Plasmid DNA extraction was performed utilizing an OMEGA Spin Miniprep kit (Omega Bio-Tek, USA), and the accuracy of the cloning was confirmed through Sanger sequencing carried out by Sangon Biotech (Shanghai, China).

2. DNA and protein sequences used in this study

The sequence of the synthesized *sacB* gene

tctttaggcccgtagtctgcaaatccttttatgattttctatcaaacaaaagaggaaaatagaccagttgcaatccaaacgagagtctaatagaatgaggtc
gaaaagtaaatcgcgcgggtttgttactgataaagcaggcaagacctaaaatgtgtaaagggcaaagtgtatactttggcgtcaccccttacatattttag
gtcttttttattgtgcgtaactaacttgccatcttcaaacaggagggctggaagaagcagaccgctaacacagtacataaaaaaggagacatgaacgat
gaacatcaaaaagtttgcaaaacaagcaacagtattaacctttactaccgcactgctggcaggaggcgcaactcaagcgtttgcgaaagaaacgaacc
aaaagccatataaggaaacatacggcatttcccatattacacgccatgatatgctgcaaatccctgaacagcaaaaaaatgaaaaatatcaagttcctga
attcgattcgtccacaattaaaaatatctcttctgcaaaaggcctggacgtttgggacagctggccattacaaaacgctgacggcactgtcgcaaactatc
acggctaccacatcgtctttgcattagccggagatcctaaaaatgcggatgacacatcgatttacatgttctatcaaaaagtcggcgaaacttctattgac
agctggaaaaacgctggccgcgtctttaaagacagcgacaaattcgatgcaaatgattctatcctaaaagaccaaacacaagaatggtcaggttcagc
cacatttacatctgacggaaaaatccgtttattctacactgatttctccggtaaacattacggcaaacaaacactgacaactgcacaagttaacgtatcagc
atcagacagctctttgaacatcaacggtgtagaggattataaatcaatctttgacggtgacggaaaaacgtatcaaaatgtacagcagttcatcgatgaa
ggcaactacagctcaggcgacaaccatacgctgagagatcctcactacgtagaagataaaggccacaaatacttagtatttgaagcaaacactggaac
tgaagatggctaccaaggcgaagaatctttatttaacaaagcatactatggcaaaagcacatcattcttccgtcaagaaagtcaaaaacttctgcaaagc
gataaaaaacgcacggctgagttagcaaacggcgctctcggtatgattgagctaaacgatgattacacactgaaaaaagtgatgaaaccgctgattgc
atctaacacagtaacagatgaaattgaacgcgcgaacgtctttaaaatgaacggcaaatggtacctgttcactgactcccgcggatcaaaaatgacgat
tgacggcattacgtctaacgatatttacatgcttggttatgtttctaattctttaactggcccatacaagccgctgaacaaaactggccttgtgttaaaaatgg
atcttgatcctaacgatgtaacctttacttactcacacttcgctgtacctcaagcgaaaggaaacaatgtcgtgattacaagctatatgacaaacagaggat
tctacgcagacaaacaatcaacgtttgcgccaagcttcctgctgaacatcaaaggcaagaaaacatctgttgtcaaagacagcatccttgaacaaggac
aattaacagttaacaaataa

The sequences highlighted in cyan represent the promoter used for controling the expression of *sacB*, while the underlined DNA sequences encode SacB protein.

Codon optimized DNA sequence for *didA*

ATGGGCACCGCGCCGCATTTTATTGCACATCAGAATATTTGGCTGGATGAAAGATTAAATA
CCGGCCCGGGTAGCTATCATATTGGCGGTTATGTCGAACTGCGCGGTCCGCTAGATATTGA
TATTTTTGGAGCTGCACTGAAAGCAGTCGCTGTTGCGCACGCAGGCCTCCACATGCAGCCG
GTACCGGAAGGTGACGGGTTTCGTCTCCTGACCTATCAGGATACCTTTAGCAGCCCGGTTA
GCGTTGATCTGAGCCAGGAGGAAAATCCGGTTGCAGCCGCAAATGCATGGATGGCAGCGG
ATTTTCGTCGTATATTTCCGGCAGGTGCACCACTCTTTCGTTGGGGTCTGATTCGTCTTGCC
GCAGATCGACATTTTTGGACCAAGACCTATCATCATCTGATAGTTGATGGTCATGCTATTA
GCCGTATTGTTCAGGATGCGGCACATGCATATACCAGTCTGCTGGCGGGACGTGATCCCGG
TCTGGCAGGTAGCGATCCGCTGGCGATCGGTGATCCGCTCGCCGGAGATGGTGTTAGCGCA
CGTCGTCATTGGAGCACCGTGCTGGCCGCAGCACCTCCCGTTCTGAATCCGGTTCCGCCCTT
GCCTCCGCCTCATTTTGGTCCACCGGACTTTGGTCCTCCGGATCTGGCACCGCCCGAGCCG
GACGTTCCAGGTCGTCGTACCCGCATCGGTCTGGAACGTCGTGTTTATGATGGATTGGCGC
GTCTAGCAGCAGAGGCCGGTGCCGGCCTCCCGCAGCTGCTTCTCGTTCTTGCCAGCGCAGC
GCTCTTAAGGCGTGCAGGTCGTGATGCTCTAGTTGCCGGCTTGCCGGTTGCAAATCGTCCT
ACCGCAGCACATAAAGCAGCCGTTGGTCTCTTTGCAACCATGCAACCAGCAATTGTCGCAA
TCGATGCAACCGATCGGCCGCTGGATGTTGCTGCAGCACTGGCTAGACGTATGCGTAGCGC
ATATCGTCATCGTAGCATTGCCTTTGCAGAGCAGGCTGCCATGCTCGCTGCTGTTCGTGGG
AGAGGTACACCGGCTTTTGACCTGACACTGTCGTTTGAGCCGCATGATTATGATGCCCGTT
TCGGACCGGCAACCGCAACTGCACATACCCTGAGTACCGGCCGTGAACCGCATCCACTGA
GCATTTTTGTTAGAGATTATCATGATGGTGCAGCGGTTACCGTTGATTTAGATTGGCGTAGC
GATGTCTTTGCAGCAGATGATATTCCAGCGCTGATTGCAAGGTTTGATCGTCTGTGTCATCG
TCTGCTGGATCAGCCTGAAGCAAGCCTGGGTAGACTGGATCGACCTGATGCAGAGACCCT
GGCCCGTCTCGGTCGGCTGGGACGTGGTCGTCCGCCGCGTCCGGGTGATCTCGCACCCCTG
GCTGATGCAATTGCAGCCCAAGCCGCAGCAACACCCGCAGCTCTGGCAGTTGTTGATGCAG
CTACCGGTGATCGTGTCGATTACCAGGGTCTGGTCGCCCGTGCAGGACGCCGTGCAGCAGC
GCTACAGCGCGCAGGGGCAGGTCCGGAAAGCGTTATTGGCCTGGCACACCCGAGCGGTAT
CGAAACCGTTGTAAGCCGTCTGGCGGTTAGCATGGCAGGTGCCGCATGGCTGCCACTGGAT
CCGGAAGATCCTCCAGCCCGTCAGGCGGCAATGCGTGATCAGGCACGTCCGCTGTTGGTAT
TAGATGATCAGAGCGCAAGAGCACTGGACCTGACCGTGGCAGCCGATGCACGCCCAGCCG
CACGCCCAGTTGATCCGGAACAGCTGGCATATCTGCTGTTTACCAGCGGTAGCACCGGCAC
GCCAAAACCGGTGGCTGTTCCGCATCGTGCCCTGGCGATGCACATGGCATGGATGGGTCGT
AGGTATCCGCTGGGTCCGGATGATGTAGTTCTGCAGAAAACCCCGGCAGGTTTTGATGCGA
GCATCTGGGAATTTCTGGCACCGCTGATGGCAGGTGCTAGACTGGTTCTGGCCCCGGCAGG
AAGCCATCGTGATCCTGAAATGATCGGTAGACTGTGTGCAGATTATGGTGCGACCATCCTG
CAGGCAACACCGACCCTGATTGATGCATTAGCAGCAAGCGGTGCGCTAGCTAGGGCAAGC
CGGCTGCGTCGTCTGTTTGCAGGTGGCGAAATTCTGGGTCCGGCGACCATTGCAGCAGCAC
GTGCAGCCCTGCCTGCCGATGCAGCCTTGATTAATCTGTATGGTCCGACCGAATGCTGCAT
TGATGCAAGCGCGGCGGACATCCCGGCTGACCTACAGGGTGCTGCACCACTGGGTGATCC
GGTGGATGGTGCAAGTTTGCAGGTTATCGATGCCGCGGGCGATCCGGTTGGTCCGGGTGTT
GCAGGTGAACTGGCTATTGGTGGCTTGGCAGTTGGTAGAGGTTATCATGGTGATCCAGTGA
GAACAGCCCTGGCATTTCGGCCGGATCCGGAAGCAGAAATGCCGGGAGCCCGTCGTTATC
TGACCGGTGATCGTGTTCGGCGTACGGAACGTGATGCGCTCCTGGCCCTGGGTCGTATTGA
TGGTCAGATCAAACTGGGTGGTCGTAGGATCGAACCGGGAGAAATTGAAGCGGCACTGAT
GGCACATCCTGCAATTGCACAGGCCGGTGCAGCATTATTGCCAGCGACGGATGGTGCAGC
ACCGCGCCTGGGTGCATGTATTGTTCTGCGTCCGGGTCACCCGGCACCCGCACCGGATGAA

CTGCGTCGTCATCTGGCTGCGCGACTACCGGCAGTTCTGCATCCGGCAGTGGTTACGACGG
CTGATCGTCTCCCTCTGAGCAGAAGCGGTAAACTGGATCGTCGTGCACTGGGTCAGGCTAT
GGCAGGCGAAGCACCAGCAACGGCGGCACGGGCCCGGCTGGATGGTCCGCTGCAGGCAG
AAATTGCCGCGATTTGGGCAGAAGTTCTCGATCTGCCGATGCCTGGTAGCGGTGCAGATTT
CTTTGCACTGGGTGGTCATAGCCTGAAAGCAATGCAGGTTGCAACCCGTCTAAGAGCGGTT
TTCGATGTTGAAATTGGTCTGGAAGAACTGTTCGACCATCCAAGGCTGGATGACCTCGCAG
CATTGGTTGCAGCCCGTAGGCGAGATGCACCAGCGGCACCGGGGCCAGTTCTGGACATTCT
TCCGGTTCCGGCAGGTAGTCTCCGTCCGCTGAGCTTTGCACAAGAACGCATTTGGTTTCTG
GCACAGTGGCCTGATGGTGCTGTCGCTTATAATATGGCAATGGCAGTTCGTATCGATGGTC
CTCTGGATGTGGCTGCCTTTGCCGAAGCTGCTGCCGTTCTCCCGGATCGTCATCCGATGCTG
CGTACCTGTTTCGCCGCACCCGATGGCGTCCCGCTGCAGCGTGTTGATCCTGAGGCACGAT
TGGTGCTGGCCACCCGTGATCTTCGAGATCTGGATCCGGCCACACGTGATGCCGCAGTTAC
CGAAGCAGCTGCGAGAGATGCCGCAACACCGTTTGATCTGACACGTGCACCGCTACTGCG
GCTGACCCTGCTGCGATTAGCAGATGACGCTCATGTGCTGCTCGTTAATCTGCATCATATTG
CGGGTGACGGTTGGTCAGGTCAGATTGTTCTGGAAGAACTGACCGCGCTGTATGCAGCACG
TATTGGTGCAGGTCCGGATCTGCCACCCGCGCCTGCGCTGACCTATGCAGACGTTGCAGAT
TGGCAGCGACGGCGTCTGAACGAGGCAGAAGCAAGGCGTCAGCTGGATCATTGGCGTGGT
GTCCTCACTGACCCGCCTGTCTTGGATCTGCCTACCGATCGTCCTCGTCCGCCTGTCGTGAG
CAGCGACGGGGGCTGCCTGGTAAGGGATCTGCCTGCAGATCTGCCGGACGATCTGCGTCGT
CTGGCAGCCGCTGCGGGTGGTACACCGTTTATGGTTATGACCGCGCTGTTTGGTTTGCTCCT
GGGTCGTATGGCGGGTCAGGACGAAGTTGTGATCGGAACCCCGGTTACCAATCGGCCGGA
TAGAAGACTGGAAGATCTGGTTGGTTTTTTTACCAATACGCTGCCGTTACGTCTCGATCTGC
GTGGTGCTGATCTGCCTGCATTACTCAGCCGTACCCGCGCAACCTGTCTGGCAGCCTTTGAT
CATCCTGATCTCCCTTTTGAACGTCTGGTTGATGCGTTCGCACCGGAACGGTCTTTGGCACA
TACCCCACTGTTCCAGGTTATGCTGGCATGGCAGCTGGCAGGTGCATCACGGCTGGAATTA
CCTGGTCTGGCCGTTAGCCGTCTGGCGCCTGCATTTACTGCAGCAAAATTTGATCTGATGCT
GAGCGTTGAAGATAGCGGCCGTAGCATGAGCTGGCGTTTTGATTATCGTGCAGAACTGTTT
GAACCGCGAACAATTGCCCTTATTGCTGATCGCCTGACCGCACTGATCCGCGCGGCGGCCC
GTCATCCGGCAGCACGTCTGCCTGAACTGTTTGAAGCACCTGTTCCGACTGTTCCTGCAAG
TCCGGCACCTGCAAGCGATGTTGTTGACGTTATTCTCGCACGTCTGGATGCAGACCCGGGT
CGGATTGCGCTGGTAGATGCGGCAACCGGTATTAGCCTGACCGCTGCAGAACTGGCTGCGC
GCGCCCGGCGTCGAGCAGCCGGTTTGGTTGCAGCTGGTATTGGCCCGGATCGTATTGTGGC
GCTGGCCCATCCGCGTGGTGCTGAACTGGTCGTTAGTATGCTGGCAGTAGCCATTGCAGGC
GGTGCATGGCTGGCACTGGATGAAGATGAGCCCGAAGCAAGGCGTGCACTGATTCTGGCA
ACCGCCGCACCGGATCTCGTCCTGGACGCGGATGGTGCAGCACGTATCGATGCGGCAGCG
GGTGATCATCCCCTGCCGGCAGGTGCGCGTCATCCCGATCGCCTGGCTTATCTGCTGTTTAC
CTCCGGTAGCACCGGCACGCCGAAACCGGTTGCAGTTCCGCATCGTGCACTGGCTCGTCAC
ATGGCATGGATGAACCGTAGCTTTCCGCTGGATGCAGCAGATGCAGTGCTGCAGAAAACC
CCGACCGGTTTTGATGCGTCTGTTTGGGAATTTCAGGCACCGCTGATGACAGGTGCCCGGC
TGGTTCTGGCACCGAGCGGAAGCCATCGAGCACCTGAAACCCTGGGCCGGTTGCTGGACG
CGCACGGTATTACCATCCTGCAAGCAACCCCGACACTGGTTGATGCCTTGGCCGCTACGGA
CGCCCTAGCACGTGGTCGTCGTCTGCGTCGTCTGTTTATTGGTGGTGAAGCACTAGGTACA
CCGACCATTCGTGCCGCACGTGCGACCCTGCCCGCAGGTTGTGCAGTGATTAACCTGTATG
GCCCGACCGAATGTTGTATTGATGCAACAGCATGGGTTGCAGATGATCTGCCGCAGGATCT
GCCGCAGACCGGGACCGCGCCTCTGGGCGCAGCTATTGATGGCGTTCATCTGATGGTTCTG
GATAGGACCGGTCGTCCGGTTCCGCCGGGTCTGCCGGGCGAACTGGCTATTGGCGGTGAA
ACCGTTGGTCGTGGTTATCATGGTGATCCGGTTCGTACCGCACTAGCATTTAGGCCGGATC
CTGCAGCACCCCGTCCTGGTGCCCGTTGTTATCTGACCGGTGATCGTGTGCTAGTTGATGCA
CGTGGTCGGCTCCGGTATCAGGGTCGTATTGATGCCCAGATGAAACTGCGAGGTCAGCGTA
TTGAACCGGGTGAAATTGAAGCAGTGCTGAGCGCACATCCGGCAATAGCACGGGCGGGTG

TGACCGTTGTTAGCGCTGAAAGTGGTGAAACCCTGGTGGCATGGTTGACATGTCATCCTGG
TCAGGCCCTTCCTGCAGCTGACGTCCTGCGTCGTCATGTTGCCCGTCATCTGCCGATTCACA
TGATTCCGGCACGTTTTGTCGCTACGGATAGCCTACCCCTAACCGCATCCGGTAAATTAGA
TCGTAGAGCATTAACAGCACGCCCGCTGCCGGCCGCGGATCGTCCCGCAGGTGGAGATGC
CCCGGCGAGTCCGGTTGAAATTCTGATTGCAGATATTTGGGCAGAGGTTCTGGATCGGCCG
GCAATCGCCCGCAGCGATGATTTTTTTGCTTTAGGTGGTCATTCTCTGAAAGCAGTTCGTAT
GCTGGCTAGGCTGAATGAAGCACTGCCGGTAGAAGTGCCTCTCCGTACCGTTTTTGAAAAC
CCTACCGTCGCCACATTGGCGGCTGCGCTGGATGCCCTGACAGATGCTGCGCTGGCCGGTG
ATGATGACCTGGCGGCCCTGCTGGCAGAAATTGAAGCACTGCCGGAAACCGATGGTCCGG
AAACCGATGGACCCGGTACCGATGGTCCGGAAGCGGAAACCTTTCCGGATACCGCAGCAC
AAC

DNA sequence for *didK*

ATGGCCTATCTCGACATGGGCCGCCTCGGCCGCAGCAGCGAGGTGATCTATGGCATCGGGC
TGATCATCATCTTCCTGGTGGTGCCGCTGGGCGCCGCCCAGGTGATCGGCCCGATCCAGGC
CTGGGCGATCAACACCTTCGGTGCGCCCGATGCCATGGTCAATCTGTTCGCGACCACGGCC
AATATCGTGGGCTTCCTGATCTGGATGGCGATGTTCCCGATCTGGATCCTGGGCGTCTTCCT
GGTGGTCCGGCTGCTCAACCAGCGGCCGTTCATGACCCTGATCTCGCCCACGCGCAGCTAT
AATCTGAAGCTGGTCGGCCTCGCTTTCGTGATCTATCTGCCGCTGGTGACGCTGTCCTCGCT
CGCCGGCATCTGGCTTGCCGGCCAGCCGGTGACCTTCAACTTCGTGTTCGAACCGGCAGGC
TGGATCGCGGGCCTGGCCCTCGGCCTGGTGCTCTACCTGATCCAGGCGACCTCGGACGAAC
TGGTCTTCCGCGCCTATCCCCTCCAGGGCGTCTACGCCGTCATCGGCCGCCCGGTGGTCGC
CGCCATCGGCGCCGCGGTGATCTTCTCCCTCTACCATTACAGCCCCGCCAACACGCCCGAA
ACCTATGCCAGCCTGTTCGTCGCCGGCCTGTTCGCGGCGGCACTGACGGTCAAGGCCCAGC
GCCTGGAACCGGCGATCGGCGTCCACCTCGCCAACAACCTGTCGTTGTTCTCCATCGTCCA
GAACCCCGAAGTCACCCCGGCCGAACCGATCTGGCGCCTGGCCGAACCGGTGGGCATGAG
CTGGGCGCTGGTGGCGACGGCGATTGTCGCGGCGGTGGTGTTTTGGGTGGTGTTTTTCCGG
ATCACGGGCACGGCTCTCGCCCGGACCACCCGCCACGCTGGTCTCTCCTGA

3. Chemical synthesis of compounds 12-22

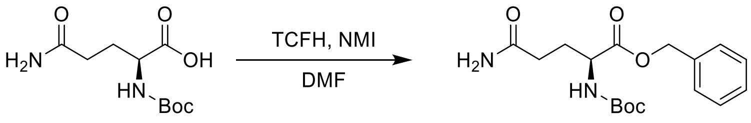

11 12

Benzyl-(*tert*-butoxycarbonyl)-L-glutaminate (12). To a solution of *N*-(*tert*-Butoxycarbonyl)-L-glutamine (3.0 g, 12.2 mmol), phenylmethanol (3.95 g, 36.6 mmol) and *N*-methylimidazole (NMI) (5.0 g, 60.9 mmol) in DMF (30 mL), chloro-*N*,*N*,*N*′,*N*′-tetramethylformamidinium hexafluorophosphate (TCFH) (10.2 g, 36.6 mmol) was added at 0 °C. Then mixture was then stirred overnight at room temperature. Afterward, the mixture was poured into saturated aqueous $NaHCO_3$ and was extracted with EtOAc (50 mL × 3). The combined organic layers were washed with brine, dried over Na_2SO_4, and concentrated under vacuum. The resulting residue was subjected to chromatography (15:1 DCM/MeOH), affording compound **12** (3.5 g, 85%) as a light yellow oil.

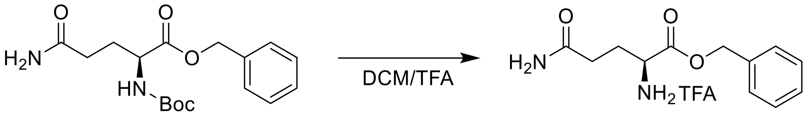

12 13

Benzyl-L-glutaminate TFA salt (13). A solution of compound **12** (3.5 g, 10.4 mmol) in DCM/TFA (10 mL/5.0 mL) was stirred at 10 °C for 2 hours. The solution was concentrated. The residue was purified by prep-HPLC (TFA) to give compound **13** (1.6 g, 46%) as a white solid.

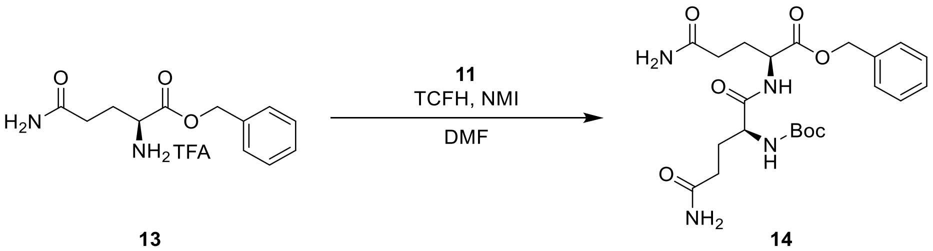

13 14

Benzyl-(tert-butoxycarbonyl)-L-glutaminyl-L-glutaminate (14). To a solution of compound **13** (1.6 g, 4.8 mmol), *N*-(tert-Butoxycarbonyl)-L-glutamine (2.36 g, 9.6 mmol) and NMI (2.36 g, 28.8 mmol) in DMF (10 mL) was added TCFH (2.69 g, 9.6 mmol). And then the mixture was stirred at 0 °C for 2 hours. The mixture was purified by prep-HPLC (TFA) to give compound **14** (800 mg, 36%) as a white solid.

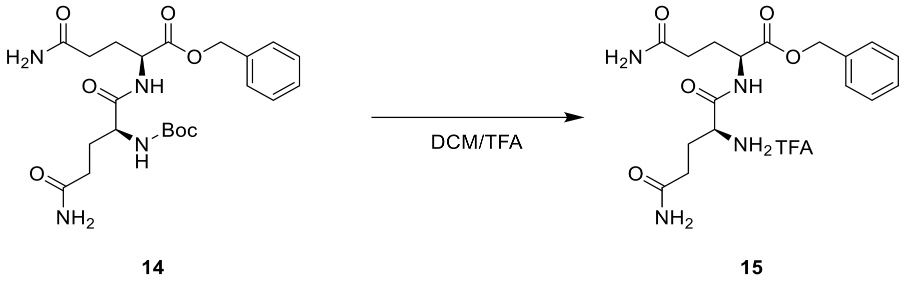

Benzyl-L-glutaminyl-L-glutaminate TFA salt (15). A solution of compound **14** (500 mg, 1.08 mmol) in DCM/TFA (2.0 mL/1.0 mL) was stirred at 10 °C for 2 hours. the solution was concentrated. The residue was purified by prep-HPLC (TFA) to give compound **15** (300 mg, 60%) as a white solid.

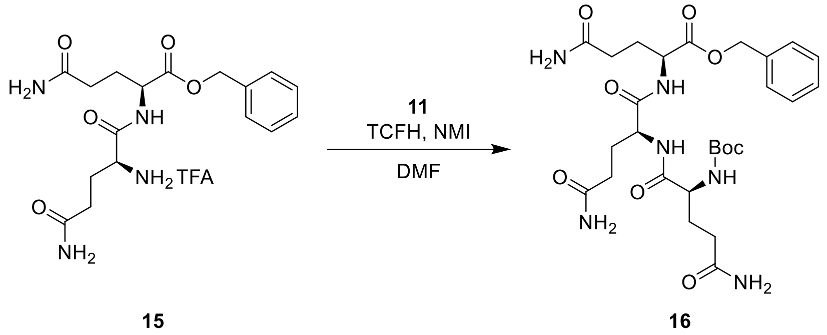

Benzyl-(tert-butoxycarbonyl)-L-glutaminyl-L-glutaminyl-L-glutaminate (16). To a solution of compound **15** (300 mg, 0.651 mmol), *N*-(tert-Butoxycarbonyl)-L-glutamine (320 mg, 1.30 mmol) and NMI (320 mg, 3.91 mmol) in DMF (3.0 mL) was added TCFH (365 mg, 1.30 mmol) at 0 °C. Then the mixture was stirred at room temperature for 2 hours. The mixture was purified by prep-HPLC (TFA) to give compound **16** (162 mg, 42%) as a white solid.

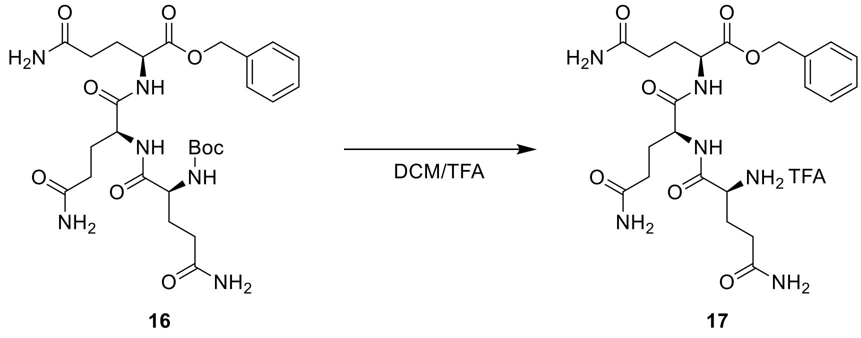

Benzyl-L-glutaminyl-L-glutaminyl-L-glutaminate TFA salt (17). A solution of compound **16** (162 mg, 0.271 mmol) in DCM/TFA (2 mL/1 mL) was stirred at 10-20 °C for 2 hours. The solution was concentrated directly. The residue was purified by prep-HPLC (TFA) to give compound **17** (118 mg, 74%) as a white solid.

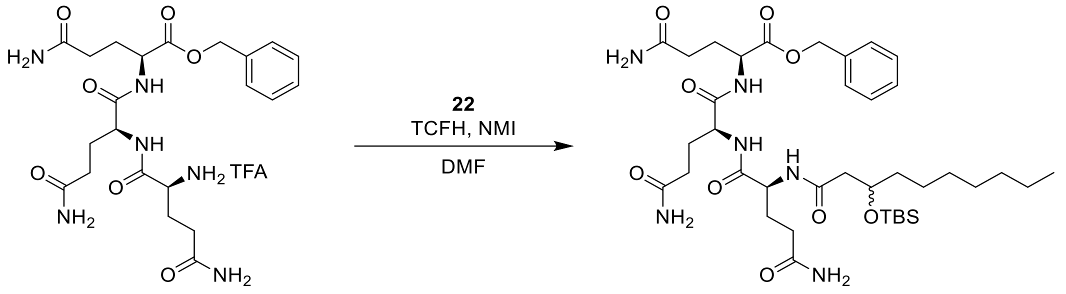

17 18

Benzyl-(3-((tert-butyldimethylsilyl)oxy)decanoyl)-L-glutaminyl-L-glutaminyl-L-glutaminate (18). To a solution of compound **17** (118 mg, 0.20 mmol), 3-((tert-butyldimethylsilyl)oxy)decanoic acid (12, 123 mg, 0.41 mmol) and NMI (100 mg, 1.22 mmol) in DMF (2.0 mL) was added TCFH (114 mg, 0.41 mmol) at 0-10 °C. Then the mixture was stirred at room temperature for 2 hours. The mixture was purified by prep-HPLC (TFA) to give compound **18** (53 mg, 33%) as a white solid.

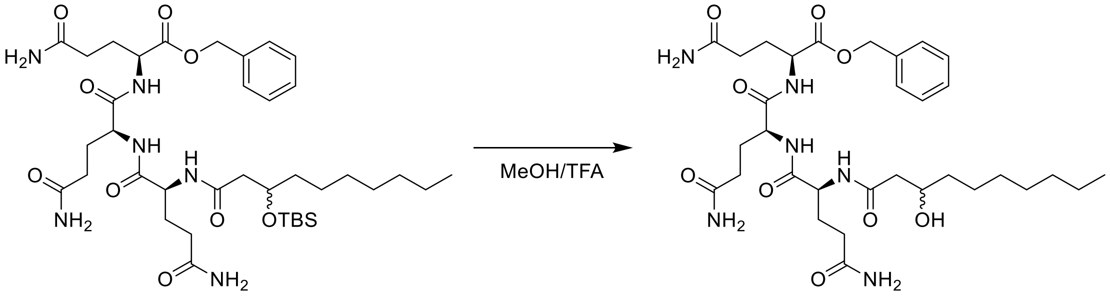

18 19

Benzyl-(3-hydroxydecanoyl)-L-glutaminyl-L-glutaminyl-L-glutaminate (19). A solution of compound **18** (30 mg, 0.04 mmol) in MeOH/TFA (2.0 mL/0.5 mL) was stirred at 10-20 °C for 2 hrs. The solution was concentrated. The residue was purified by prep-HPLC (TFA) to give compound **19** (10 mg, 39%) as a white solid.

^{1}H NMR (600 MHz, DMSO-d_6) δ 8.43 – 8.28 (m, 1H), 8.01 (d, J = 7.7 Hz, 2H), 7.40 – 7.29 (m, 5H), 7.22 (d, J = 9.7 Hz, 3H), 6.77 (s, 3H), 5.11 (s, 2H), 4.65 (d, J = 46.9 Hz, 1H), 4.25 (m, 3H), 3.79 (s, 1H), 2.21 (t, J = 6.7 Hz, 2H), 2.17 – 2.03 (m, 6H), 2.03 – 1.67 (m, 6H), 1.29 (s, 12H), 0.85 (t, J = 6.8 Hz, 3H) ppm.

^{13}C NMR (151 MHz, DMSO-d_6) δ 174.41, 174.35, 174.24, 173.71, 172.05, 172.03, 172.00, 171.84, 171.82, 171.79, 171.68, 136.36, 128.91, 128.49, 128.28, 79.65, 79.44, 79.22, 68.16, 67.92, 66.43, 52.73, 52.66, 52.38, 52.17, 44.03, 37.48, 37.40, 32.00, 31.86, 31.74, 31.54, 29.55, 29.23, 29.22, 28.48, 28.44, 28.39, 28.25, 26.93, 25.59, 25.50, 22.58, 14.45 ppm.

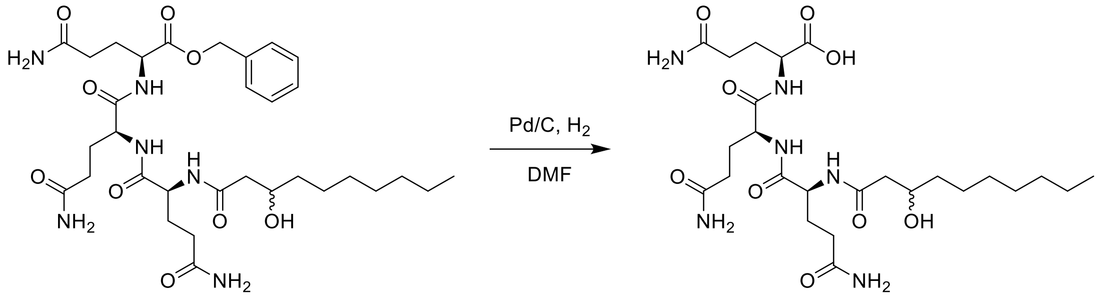

19 20

(3-hydroxydecanoyl)-L-glutaminyl-L-glutaminyl-L-glutamine (20). To a solution of compound **19** (6 mg, 0.01 mmol) in DMF (1.0 mL) was added Pd/C (5 mg, 10%, wet.) and stirred at room temperature under H_2 atmosphere (15 psi) for 2 hrs. The solution was filtered and the filtrate was purified by prep-HPLC (TFA) to give compound **20** (4 mg, 77%).

^{1}H NMR (600 MHz, DMSO-*d*$_6$) δ 12.58 (s, 1H), 8.15 (d, *J* = 7.6 Hz, 1H), 8.06 – 7.91 (m, 2H), 7.23 (d, *J* = 19.4 Hz, 3H), 6.76 (s, 3H), 4.65 (d, *J* = 5.0 Hz, 1H), 4.23 (t, 2H), 4.14 (q, *J* = 7.3, 6.8 Hz, 1H), 3.78 (s, 1H), 2.21 (t, *J* = 6.3 Hz, 2H), 2.16 – 2.05 (m, 6H), 2.00 – 1.64 (m, 6H), 1.29 (s, 12H), 0.86 (t, *J* = 6.8 Hz, 3H) ppm.

^{13}C NMR (151 MHz, DMSO-*d*$_6$) δ 174.42, 174.35, 174.31, 173.92, 173.70, 173.68, 171.83, 171.80, 171.77, 171.74, 171.65, 79.64, 79.44, 79.21, 68.15, 67.92, 52.71, 52.65, 52.44, 52.02, 44.02, 37.48, 37.40, 31.98, 31.86, 31.76, 31.74, 29.54, 29.23, 29.22, 28.49, 28.45, 28.39, 27.25, 25.59, 25.50, 22.58, 14.45 ppm.

HRMS: (ESI-TOF) m/z calcd for $C_{25}H_{44}N_6O_9$ ($[M + H]^+$): 573.3243, found: 573.3229

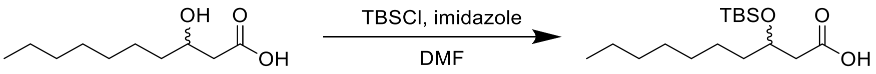

21 22

3-((*tert*-butyldimethylsilyl)oxy)decanoic acid (22). Imidazole (904 mg, 13.3 mmol) was added into a solution of TBSCl (698 mg, 4.65 mmol) in DMF under Ar and the solution was stirred at 20 °C for 30 mins. Then (+/-)-3-hydroxydecanoic acid (250 mg, 1.33 mmol) was added to the solution and the mixture was stirred overnight at room temperature. Post-treatment was performed according to reference[1]. Compound **22** (273 mg, yield 68%) as a light yellow oil.

^{1}H NMR (600 MHz, $CDCl_3$) δ 4.09 (p, *J* = 6.1 Hz, 1H), 2.61 – 2.42 (m, 2H), 1.59 – 1.48 (m, 2H), 1.38 – 1.20 (m, 10H), 0.96 – 0.78 (m, 12H), 0.10 (s, 3H), 0.08 (s, 3H) ppm.

4. Bioinformatic Analysis.

The sequence similarity network (SSN), genome neighborhood network (GNN), and genome neighborhood diagram (GND) were generated using the web-based Enzyme Function Initiative (EFI) tool [2]. The SSN was constructed by conducting a BLAST search for DidK sequences in the NCBI database, limited to the bacterial taxonomy category. An e-value of 5 and an alignment score of 45 were used for NCBI BLAST retrieval and SSN edge calculation, respectively. The GNN was generated by configuring the neighborhood size to 20 and an input co-occurrence threshold of zero. To identify DidK homologues in proximity to NRPS/PKS, we performed a search for NRPS in the GNN using Cytoscape software. Bacterial BGCs and their associated products were analyzed and predicted using antiSMASH[3]. The identified NRPS/PKS clusters containing DidK homologues were visualized on the EFI web site. Sequence identity and similarity values were calculated through pairwise sequence alignments using the NCBI BLAST web tool. Protein structure models were built using AlphaFold 2, and structural comparisons were carried out using the “align” command in PyMOL.[4]

5. Western bolt analyses

Whole-cell extracts were diluted with Biosharp 5× LDS Sample Buffer and loaded onto 10% PAGE® Bis-Tris gels (Sangon Biotech, China). The proteins were then separated through gel electrophoresis using a Bio-Rad Gel system and subsequently transferred to polyvinylidene difluoride (PVDF) membranes using an Invitrogen semi-dry apparatus at 20 V for 1 hour. A secondary antibody (ProteinFind Goat Anti-Mouse IgG (H+L), HRP Conjugate, HS201-01) was applied at a dilution of 1:10000–1:15000 in TBST with 5% BSA for 1 hour at room temperature. The membranes were subsequently subjected to four 5-minute washes with TBST. Finally, the detection of immuno-reactive bands was performed by scanning the images using a LI-COR Odyssey Imaging System (LI-COR Biosciences, USA).

6. General methods

Nuclear magnetic resonance (NMR) spectra were acquired using either a Bruker BioSpin GmbH 400 MHz spectrometer equipped with an avance Neo 400 iprobe or a Bruker BioSpin GmbH 600 MHz spectrometer with a cryoprobe prodigy. Chemical shifts are reported in parts per million (ppm) relative to tetramethylsilane. To standardize the spectra, all measurements were referenced to the (residual proton) signal of the deuterated solvent employed ($CDCl_3$ at 7.26 ppm for ^1H and 77.06 ppm for ^{13}C). The NMR experiments were conducted at a temperature of 25 °C. Reagents and chemicals were purchased from the Bide Pharmatech Ltd (Shsnaghai, China), unless stated otherwise, and were used as received without additional purification.

The metabolomics of *T. mobilis* L17 wild type strain and *T. mobilis* L17/Δ*didA(48-429)* mutant strain were performed on a Q-Exactive Orbitrap mass spectrometer (Thermo Fisher Scientific, USA) coupled to a Vanquish HPLC system. A 1.0-µL sample was injected into C18 porous core column (ACQUITY UPLC BEH C18, 2.1 mm × 100 mm, particle size of 1.7 µm, pore size of 130 Å, waters) for analysis. For gradient elution, a high-pressure binary gradient system was used. The mobile phase consisted of solvent A (H_2O + 0.1% formic acid (FA)) and solvent B (ACN + 0.1% FA) unless otherwise specified. The flow rate was set to 0.2 ml/min, unless otherwise specified. After injection, the samples were eluted with the following linear gradients: 0–20 min, 40–90% B, followed by a 5 min washout phase at 90% B and a 5 min re-equilibration phase at 40% B. Data-independent acquisition (DIA) of MS/MS spectra was performed in positive mode. ESI parameters were set to a sheath gas flow of 50 L min^{-1}, auxiliary gas flow of 12 L min^{-1}, sweep gas flow of 2 L min^{-1} and auxiliary gas temperature of 424 °C, while the spray voltage was set to 3.5 kV, the capillary to 263 °C, and a 58.5-V S-lens level was applied. The MS scan range was set to 133–2,000 m/z. The maximum ion injection time was set to 100 ms with an automated gain control (AGC) target of 1.0 × 106. The maximum ion injection time for MS/MS scans was set to 100 ms with an AGC target of 1.0 × 106 ions. The MS/MS precursor isolation window was set to m/z 1. The normalized collision energy was set to a stepwise increase from 20 to 30 to 40% with z = 1 as default charge state. MS/MS scans were triggered at the apex of chromatographic peaks within 2 to 15 s from their first occurrence. Dynamic precursor exclusion was set to 5 s.

Supplementary tables

Supplementary table S1. List of primers used in this study.

Primer Name	Sequence
JZ002_F	ttttcttttattgtttgttagtcttgatgcttcactgatagatacaagagccataag
JZ002_R	cccacgacccctacgtacaggcaggtctgtttgccaggaactgctgaacagcaaaaag
JZ001_F	acgtaggggtcgtgggccacgaaggcgtgcacgagtactccgcggcgttgtgacaat
JZ001_R	aacctgccataggccggccgaattgacataagcctgttcggttcg
CAP01_F	ttcggccggcctatggcaggttgggcgtcgcttggtcggtc
CAP01_R	ttcttcgtcttgcatgcctgcaggtcgacggatcttttccgctg
tmdidA_LF	atcgGTGCACaccggatcgatccgcatgccgtcctcgaccttgaactg
tmdidA_LR	atcgAAGCTTtcatccagccagatgttttgatgtgcgatgaaatgtg
tmdidA_RF	atcgAAGCTTatcgtgcaggatgccgcacatgcatacacctcgc
tmdidA_RR	atcgTCTAGAcggatagcgccggcccatccaggccatgtgcatgg
tmdidJ _LF	atcgGTGCACctggtctgcggcttcatggtggtgctctatc
tmdidJ _LR	atcgAAGCTTcggccggcgggaccggtgagccagcgatcctg
tmdidJ _RF	atcgAAGCTTagcatgggtgcgatgatcggctatgaactggc
tmdidJ_RR	atcgTCTAGAtacctgcacaagatcatcattcccgaggtc
tmdidK_LF	atcgGTGCACctttaaaatggagattgataaattctcataccggaag
tmdidK_LR	atcgGGTACCtcctgacactgcgccccacggcgccggcatgtcgcac
tmdidK_RF	atcgGGTACCtgtcgggtgcccactctatcaccaggttgtggcgatctg
tmdidK_RR	atcgTCTAGAtggttcaccgaccagctgcgccggtatctgcgcatgtc
gdidJ_F	tgttcatcgccgcccccatcctgctgttcctgggag
gdidJ_R	tcagaccatgccgcgcgcggccagcacgcgcgcaa
gdidK_F	tcactaagagcccgatccatctccgcaacaatgtagtattttttgc
gdidK_R	ctcgcctattcggtgttcggctggctgcccagcattctgcaggatc
pTF FWD	aagcttgtcgacctgcagtctag
pTF REV	gggcccctggaacagaacttccagac
didK FWD	gggtctggaagttctgttccaggggccctccgcctatctcgacatgggccgcctcggcc
didK REV	agagattacctatctagactgcaggtcgacaagctttcaggagagaccagcgtggcg
E204A FWD	atccaggcgacctcggacgctctggtcttccgcgcctatc
E204A REV	gataggcgcggaagaccagagcgtccgaggtcgcctggatc
R208A FWD	tcggacgaactggtcttcgccgcctatcccctccagggcgtctac
R208A REV	tagacgccctggaggggataggcggcgaagaccagttcgtccgaggtc
F233A FWD	atcggcgccgcggtgatcgcatccctctaccattacag
F233A REV	tgtaatggtagagggatgcgatcaccgcggcgccgatg
H237A FWD	tgatcttctccctctacgcatacagccccgccaacacgc
H237A REV	tcgggcgtgttggcggggctgtatgcgtagagggagaagatcacc
H274A WD	tggaaccggcgatcggcgtcgcactcgccaacaacctgtcg
H274A REV	aacgacaggttgttggcgagtgcgacgccgatcgccggttccagg
N278A FWD	atcggcgtccacctcgccaacgcactgtcgttgttctccatc
N278A REV	acgatggagaacaacgacagtgcgttggcgaggtggacgccgatc
DidA-Gln1 FWD	taactttaagaaggagatataccatggatggcaccgcgccgcattttattgcacatcag
DidA-Gln1 REV	tcagtggtggtggtggtggtgctcgagacggagactacctgccggaaccggaagaatg
DidA-Gln2 FWD	ttaactttaagaaggagatataccatggaggcgagatgcaccagcggcaccggggccag
DidA-Gln2 REV	tcagtggtggtggtggtggtgctcgagttgtgctgcggtatccggaaaggtttccgctt

Supplementary table S2. Plasmids used in this study.

Plasmid	Description	Source/ Reference
pJZ001	Plasmid construction, gentamycin resistance, *oriT*	[5]
pJZ002	Plasmid construction, temperature-sensitive *oriC*	[5]
pTHZ000	A plasmid for making gene deletion in *T. mobilis*, gentamycin resistance, *oriT*, phiC31, *attP*, *sacB*	This study
pTHZ001	A plasmid for making gene deletion in *T. mobilis* with a MCS, gentamycin resistance, *oriT*, phiC31, *attP*, *sacB*	This study
pTHZ001::*didA(48-429)*	pTZ002 derivative for deleting the gene *didA*(48-429) in *T. mobilis* L17	This study
pTHZ001::*didJ(69-300)*	pTZ002 derivative for deleting the gene *didJ*(69-300) in *T. mobilis* L17	This study
pTHZ001::*didK(1-1023)*	pTZ002 derivative for deleting the gene *didK*(1-1023) in *T. mobilis* L17	This study
pET-28a(+)	Plasmid for *N*- and *C*-terminal his-tagged fusion protein expression.	Novagen
pET-28a(+)-*didA*	Plasmid for DidA expression with *N*-terminal his-tag based on pET-28a(+)	This study
pET-28a(+)-*didA_Gln1*	Plasmid for DidA domain 1 expression with *N*-terminal His-tag based on pET-28a(+)	This study
pET-28a(+)-*didA_Gln2*	Plasmid for DidA domain 2 expression with *N*-terminal His-tag based on pET-28a(+)	This study
pCold-TF	Plasmid for cold shock-induced expression in *E. coli* of a protein fused to the Trigger Factor (TF) chaperone and a *N*-His-tag	Invitrogen
pCold-TF-*didK*	Plasmid for DidK expression based on pCold-TF	This study
pXZ_D203A	Plasmid for DidK_ D203A expression based on pCold-TF-DidK	This study
pXZ_E204A	Plasmid for DidK_ E204A expression based on pCold-TF-DidK	This study
pXZ_R208A	Plasmid for DidK_ R208A expression based on pCold-TF-DidK	This study
pXZ_H233A	Plasmid for DidK_ H233A expression based on pCold-TF-DidK	This study
pXZ_H237A	Plasmid for DidK_ H237A expression based on pCold-TF-DidK	This study
pXZ_N278A	Plasmid for DidK_ N278A expression based on pCold-TF-DidK	This study

Supplementary table S3. Strains used in this study.

Strains	Description	Source/ Reference
Escherichia coli		
DH5α	Host for general cloning	Invitrogen
BL21(DE3)	Host for protein expression	Invitrogen
BAP1	Host for protein expression	Invitrogen
E. coli S17-1	Donor strain for conjugation	ATCC
Tistrella mobilis L17	Didemnin wild-type producer	Marine Culture Collection of China (MCCC), MCCC1A11766
T. mobilis L17/*ΔdidA*(48-429)	A *didA* deletion mutant of *T. mobilis* L17	This work
T. mobilis L17/*ΔdidJ*	A *didJ* deletion mutant of *T. mobilis* L17	This work
T. mobilis L17/*ΔdidK*	A *didK* deletion mutant of *T. mobilis* L17	This work

Supplementary table S4. ^{1}H NMR (400 MHz, $CDCl_3$) and ^{13}C NMR data for didemnin B (**1**, $CDCl_3$) and nordidemnin B (**2**, $CDCl_3$).

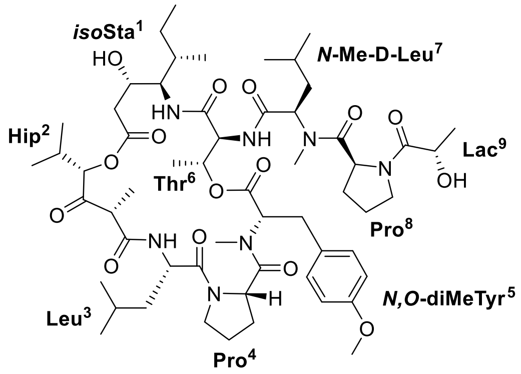

didemnin B (**1**)

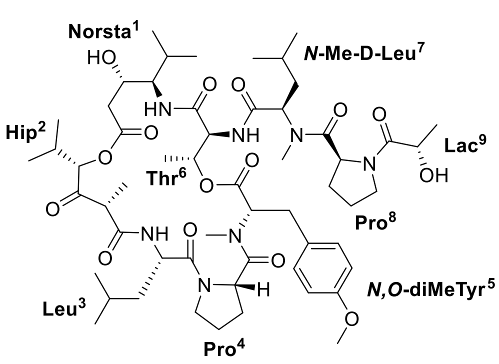

nordidemnin B (**2**)

	Position	δ_C	δ_H, multi., *J* in Hz		Position	δ_H, multi., *J* in Hz
*iso*Sta[1]	C=O	172.6	-	Norsta[1]	C=O	-
	2a	38.9	2.64, m, 1H		2a	2.68, dd, 15.3, 11.6, 1H
	2b		3.25, m, 1H		2b	3.19, m, 1H
	3	68.1	4.05, m, 1H		3	3.99, m, 1H
	4	55.6	4.10, m, 1H		4	4.07, m, 1H
	5	34.1	1.68−1.83, m, 1H		5	1.75−1.84, m, 1H
	5-CH_3	14.8	0.85−0.94, m, 3H		6	0.84−0.97, m, 3H
	6a	27.3	1.16−1.21, m, 1H		7	0.84−0.97, m, 3H
	6b		1.40−1.44, m, 1H		NH	7.31, d, 9.9, 1H
	7	11.8	0.85−0.94, m, 3H			
	NH	-	7.22, d, 9.3, 1H			
Hip[2]	C=O	169.8	-	Hip[2]	C=O	-
	2	49.7	4.22, q, 6.8, 1H		2	4.24, q, 6.5, 1H
	2-CH_3	15.4	1.31, d, 6.8, 3H		2-CH_3	1.32, d, 6.5, 3H
	3	205.1	-		3	-
	4	81.6	5.16, d, 3.3, 1H		4	5.16, br s, 1H
	5	31.4	2.34, m, 1H		5	2.34, m, 1H
	6	17.0	0.85−0.94, m, 3H		6	0.84−0.97, m, 3H
	7	18.7	0.85−0.94, m, 3H		7	0.84−0.97, m, 3H
Leu[3]	C=O	171.4	-	Leu[3]	C=O	-
	2	49.6	4.80, m, 1H		2	4.81, m, 1H
	3a	41.4	1.18−1.22, m, 1H		3a	1.19−1.22, m, 1H
	3b		1.59, m, 1H		3b	1.58−1.70, m, 1H
	4	25.0	1.51, m, 1H		4	1.50−1.54, m, 1H
	5	21.0	0.85−0.94, m, 3H		5	0.84−0.97, m, 3H
	6	23.8	0.85−0.94, m, 3H		6	0.84−0.97, m, 3H
	NH		7.82, d, 9.2, 1H		NH	7.78, d, 9.1, 1H
Pro[4]	C=O	170.7	-	Pro[4]	C=O	-
	2	57.3	4.63, m, 1H		2	4.63, m, 1H
	3a	28.0	1.68−1.83, m, 1H		3a	1.70−1.82, m, 1H
	3b		2.11−2.14, m, 1H		3b	2.12−2.16, m, 1H
	4a	25.1	2.00−2.03, m, 1H		4a	1.97−2.02, m, 1H
	4b		2.18−2.22, m, 1H		4b	2.21−2.23, m, 1H
	5a	47.1	3.54−3.60, m, 1H		5a	3.56−3.61, m, 1H
	5b		3.65−3.68, m, 1H		5b	3.67−3.70, m, 1H
N,*O*-diMeTyr[5]	C=O	168.6	-	*N*,*O*-diMeTyr[5]	C=O	-
	2	66.6	3.54−3.60, m, 1H		2	3.56−3.61, m, 1H
	3a	34.0	3.18, d, 14.2, 1H		3a	3.13, m, 1H
	3b		3.37, dd, 14.2, 3.8, 1H		3b	3.38, d, 14.3, 1H
	4	130.1	-		4	-
	5/5’	130.4	7.06, d, 8.3, 1H		5/5’	7.07, d, 8.0, 1H
	6/6’	114.2	6.83, d, 8.3, 1H		6/6’	6.84, d, 8.0, 1H
	7	158.7	-		7	-
	OCH_3	55.4	3.78, s, 3H		OCH_3	3.79, s, 3H
	NCH_3	38.8	2.55, s, 3H		NCH_3	2.58, s, 3H

Thr[6]	C=O	169.6	-	Thr[6]	C=O	-
	2	57.8	4.54, m, 1H		2	4.58, m, 1H
	3	70.6	5.33−5.39, m, 1H		3	5.35−5.41, m, 1H
	4	16.4	1.36, d, 5.0, 3H		4	1.38, d, 6.3, 3H
	NH		7.65, d, 5.2, 1H		NH	7.68, d, 4.5, 1H
N-Me-D-Leu[7]	C=O	171.9	-	*N*-Me-D-Leu[7]	C=O	-
	2	55.0	5.33−5.39, m, 1H		2	5.35−5.41, m, 1H
	3a	36.3	1.68−1.83, m, 1H		3a	1.70−1.82, m, 1H
	3b		1.68−1.83, m, 1H		3b	1.70−1.82, m, 1H
	4	25.0	1.38−1.41, m, 1H		4	1.41−1.44, m, 1H
	5	23.5	0.85−0.94, m, 3H		5	0.84−0.97, m, 3H
	6	21.5	0.85−0.94, m, 3H		6	0.84−0.97, m, 3H
	NCH_3	31.4	3.15, s, 3H		NCH_3	3.14, s, 3H
Pro[8]	C=O	173.0	-	Pro[8]	C=O	-
	2	56.9	4.77, m, 1H		2	4.75, m, 1H
	3a	28.5	1.94−1.98, m, 1H		3a	1.97−2.02, m, 1H
	3b		2.18−2.22, m, 1H		3b	2.12−2.16, m, 1H
	4a	26.0	1.94−1.98, m, 1H		4a	1.97−2.02, m, 1H
	4b		2.18−2.22, m, 1H		4b	2.21−2.23, m, 1H
	5a	47.2	3.54−3.60, m, 1H		5a	3.56−3.61, m, 1H
	5b		3.65−3.68, m, 1H		5b	3.67−3.70, m, 1H
Lac[9]	C=O	174.0	-	Lac[9]	C=O	-
	2	66.2	4.38, q, 6.5, 1H		2	4.39, q, 6.2, 1H
	3	20.3	1.38, d, 6.5, 3H		3	1.41, d, 6.2, 3H

Supplementary table S5. ^{1}H NMR and ^{13}C NMR data for didemnin X (**3**, $CDCl_3$).

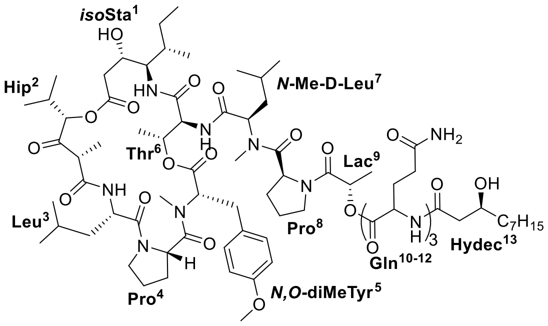

didemnin X (**3**)

	Position	δ_C	δ_H, multi., *J* in Hz
*iso*Sta[1]	C=O	172.6	-
	2a	38.7	2.60, m, 1H
	2b		3.11, m, 1H
	3	67.8	4.01−4.03, m, 1H
	4	56.0	4.01−4.03, m, 1H
	5	34.3	1.74−1.80, m, 1H
	5-CH_3	15.0	0.84−0.91, m, 3H
	6a	27.0	1.15, m, 1H
	6b		1.37−1.41, m, 1H
	7	11.8	0.84−0.91, m, 3H
	NH	-	7.45, d, 7.5, 1H
Hip[2]	C=O	169.8	-
	2	49.7	4.12, q, 6.5, 1H
	2-CH_3	15.5	1.33, d, 7.0, 3H
	3	204.8	-
	4	81.8	5.13, d, 3.6, 1H
	5	31.2	2.26−2.35, m, 1H
	6	17.1	0.84−0.91, m, 3H
	7	18.8	0.84−0.91, m, 3H
Leu[3]	C=O	171.3	-
	2	49.7	4.81, m, 1H
	3a	41.5	1.20−1.23, m, 1H
	3b		1.54−1.65, m, 1H
	4	24.7	1.50−1.54, m, 1H
	5	21.1	0.84−0.91, m, 3H
	6	24.0	0.84−0.91, m, 3H
	NH		7.89, d, 8.7, 1H
Pro[4]	C=O	170.6	-
	2	57.1	4.59, m, 1H
	3a	28.1	1.74−1.80, m, 1H
	3b		2.09−2.17, m, 1H
	4a	25.2	2.04−2.17, m, 1H
	4b		2.04−2.17, m, 1H
	5a	47.2	3.56−3.64, m, 1H
	5b		3.67−3.72, m, 1H
N,*O*-diMeTyr[5]	C=O	168.6	-
	2	66.3	3.58−3.69, m, 1H
	3a	34.0	3.18, d, 14.0, 1H
	3b		3.33, dd, 14.0, 3.5, 1H
	4	129.9	-
	5/5’	130.5	7.08, d, 8.5, 1H
	6/6’	114.3	6.83, d, 8.5, 1H
	7	158.8	-
	OCH_3	55.4	3.78, s, 3H
	NCH_3	38.8	2.53, s, 3H
Thr[6]	C=O	169.3	-
	2	57.0	4.67, m, 1H
	3	70.9	5.06, m, 1H
	4	16.0	1.34, d, 7.0, 3H
	NH	-	7.30, m, 1H

N-Me-D-Leu[7]	C=O	171.6	-
	2	54.4	5.30, dd, 9.2, 5.3, 1H
	3a	36.5	1.54−1.65, m, 1H
	3b		1.54−1.65, m, 1H
	4	25.0	1.37−1.41, m, 1H
	5	23.6	0.84−0.91, m, 3H
	6	21.6	0.84−0.91, m, 3H
	NCH_3	31.2	3.05, s, 3H
Pro[8]	C=O	173.3	-
	2	57.4	4.87, m, 1H
	3a	28.8	1.85, m, 1H
	3b		2.22−2.25, m, 1H
	4a	27.7	2.04−2.17, m, 1H
	4b		2.04−2.17, m, 1H
	5a	47.3	3.56−3.64, m, 1H
	5b		3.80−3.82, m, 1H
Lac[9]	C=O	173.5	-
	2	69.5	5.23, q, 6.5, 1H
	3	16.1	1.47, d, 6.5, 1H
Gln[10]	C=O	172.1	-
	2	52.0	4.50, m, 1H
	3	25.5	2.26−2.35, m, 2H
	4	29.8	1.93−2.17, m, 2H
	5	176.2	-
	NH	-	7.97, br s, 1H
	NH_2	-	6.59, br s, 2H
Gln[11]	C=O	172.3	-
	2	53.3	4.40, m, 1H
	3	25.9	2.26−2.35, m, 2H
	4	29.8	1.93−2.17, m, 2H
	5	176.6	-
	NH	-	8.09, br s, 1H
	NH_2	-	6.83, d, 8.5, 2H
Gln[12]	C=O	172.2	-
	2	53.1	4.40, m, 1H
	3	26.7	2.26−2.35, m, 2H
	4	29.8	1.93−2.17, m, 2H
	5	176.4	-
	NH	-	8.23, br s, 1H
	NH_2	-	7.08, d, 8.5, 2H
Hydec[13]	C=O	170.2	-
	2a	43.4	2.44, m, 1H
	2b		2.26−2.35, m, 1H
	3	69.1	3.97, m, 1H
	4	37.6	1.37−1.41, m, 2H
	5	24.0	1.21−1.29, m, 2H
	6	29.5	1.21−1.29, m, 2H
	7	29.5	1.21−1.29, m, 2H
	8	32.0	1.21−1.29, m, 2H
	9	22.8	1.21−1.29, m, 2H
	10	14.3	0.84−0.91, m, 3H

Supplementary figures

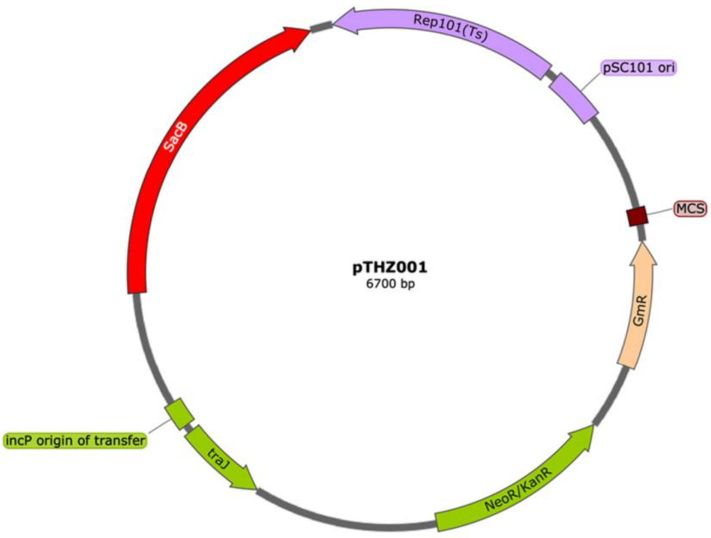

Figure S1. Plasmid map of pTHZ001.

The vector constructed by assembling four different functional elements from pJZ001 (Tan), pJZ002 (Lavender), pCAP01 (Lime), and synthesized *sacB* component (Red). [6]

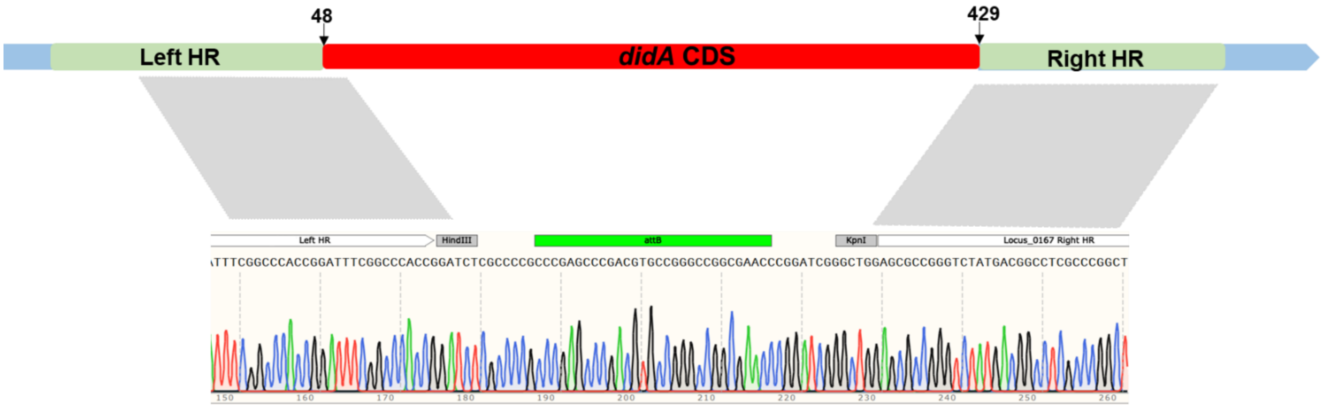

Figure S2. Verification of *T. mobilis/ΔdidA*(48-429) mutant by Sanger sequencing.

The scheme shows the deletion of *didA* from the base pair number 48 to 429 bp. The knock out method is discribed in Materials and Methods 3 section.

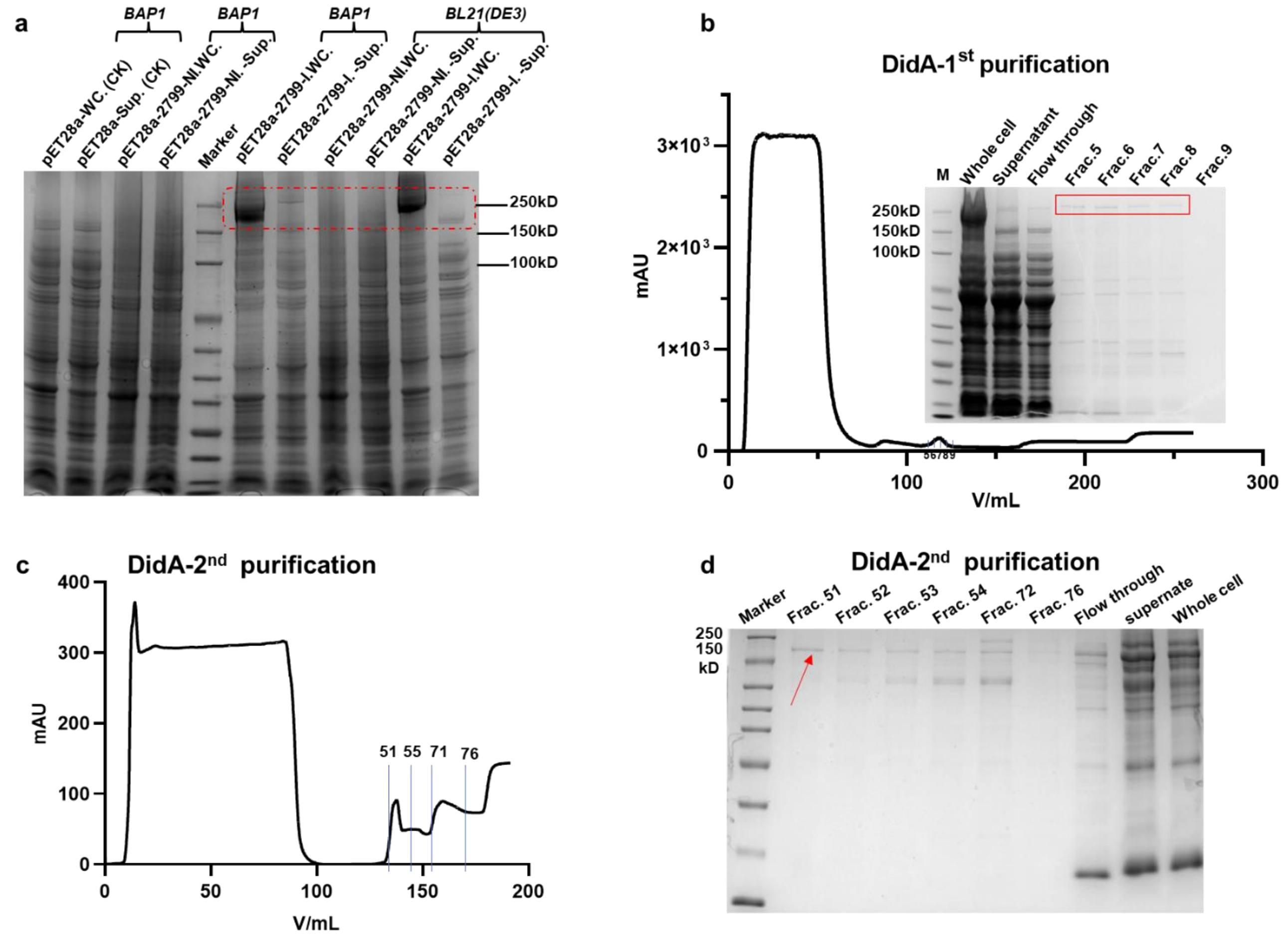

Figure S3. SDS-PAGE analysis of the expression and purification of DidA

a, The expression of DidA. Lane 1 (counting from left to right) represents a induced cell pellet of *E. coli* BAP1/pET28a, lane 2 represnets a supernant of the induced *E. coli* BAP1/pET28a, lane 3 represents a non-induced cell pellet of *E. coli* BAP1/pET28a::*didA*, lane 4 represnets the supernant of the non-induced cells of *E. coli* BAP1/ pET28a::*didA*, lane 5 represents a protein marker, lane 6 represents a induced cell pellet of *BAP1*/pET28a::*didA*, lane 7 represnets the supernant of the induced cells of *E. coli* BAP1/pET28a::*didA*, lane 8 represents a non-induced cell pellet of *E. coli* BL21(DE3)/pET28a::*didA*, lane 9 represnets the supernant of a non-induced cells of *E. coli* BL21(DE3)/pET28a::*didA*, lane 10 represents a induced cell pellet of *E. coli* BL21(DE3)/pET28a::*didA*, lane 11 represnets the supernant of the non-induced cells of *E. coli* BL21(DE3)/pET28a::*didA*.

b, The chromatogram for the first round of DidA protein purification and SDS-PAGE analysis. Lane 1 (counting from left to right) represents a protein marker, lane 2 represents a cell pellet of the induced *E. coli* BAP1/pET28a::*didA*, lane 3 represents a supernant of the induced *E. coli* BAP1/ pET28a::*didA*, lane 4 represents the flow through of the induced *E. coli* BAP1/ pET28a::*didA*, lanes 5, 6, 7, 8, and 9 represent the fractions 5, 6, 7, 8, and 9, respectively. Protein purification was performed on an ÄKTA purifier

instrument (Cytiva, US) with the modules Box-900, UPC-900, R-900 and Frac-900 with all buffers filtered through a nylon membrane 0.22-µm (Merck) before use.

c, The chromatogram for the second round of DidA protein purification; **d** SDS-PAGE analysis**:** Lane 1 (counting from left to right) represents the protein marker, lanes 2 to10 represent the fractions 51, 52, 53, 54, 72, 76, flow through, supernatant, and whole cell extract, respectively.

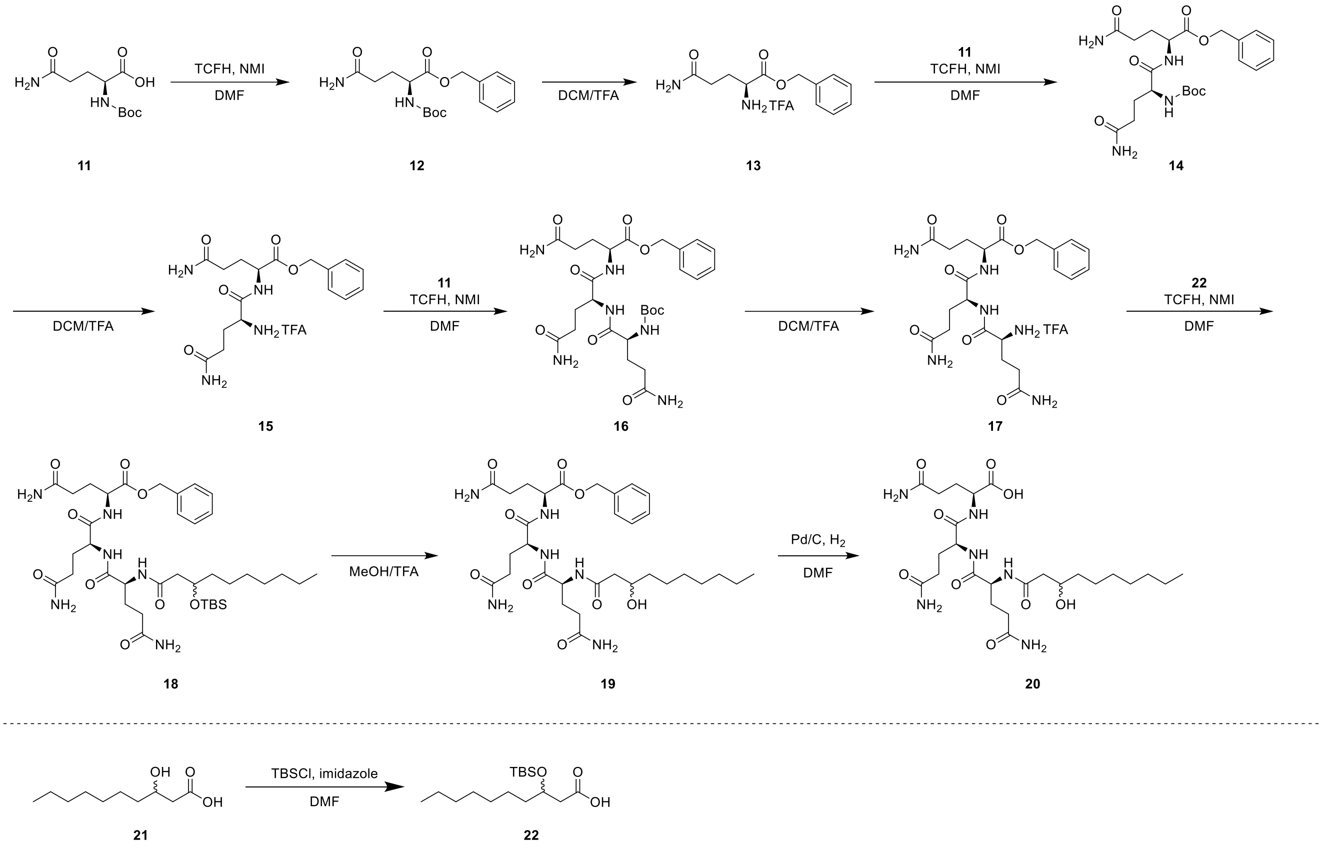

Figure S4. Chemical synthesis of compounds **20**.

a DidA Enzymatic reactions

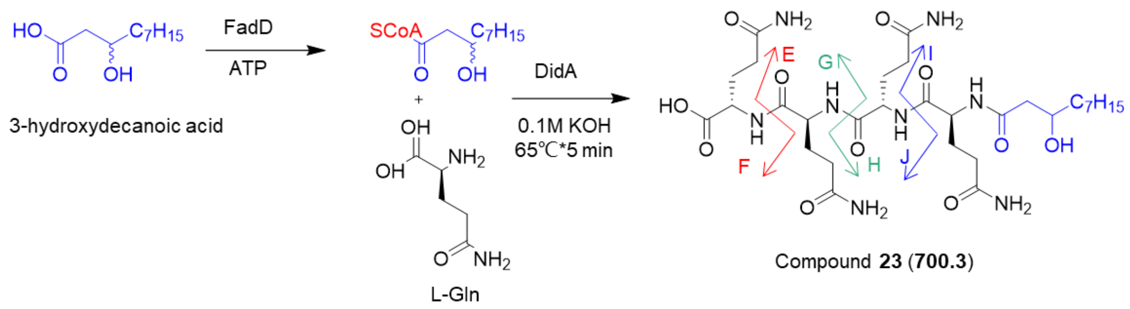

b Biosynthesized **700.3-H$_2$O** (MS/MS)

Biosynthesized **700.3-H$_2$O** (MS/MS)

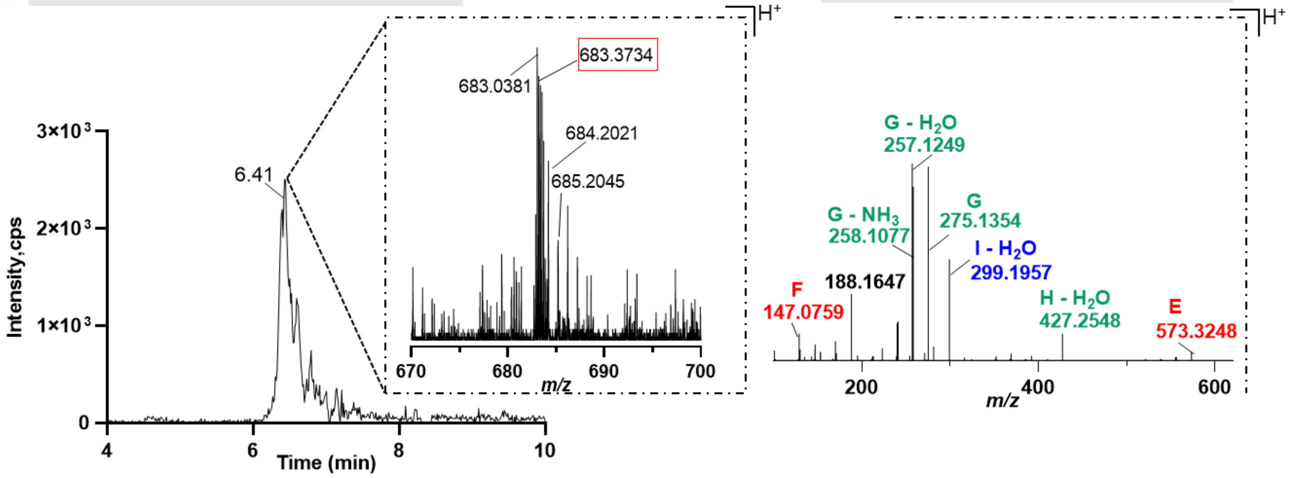

c 682.3 Biosynthesized compound **25**

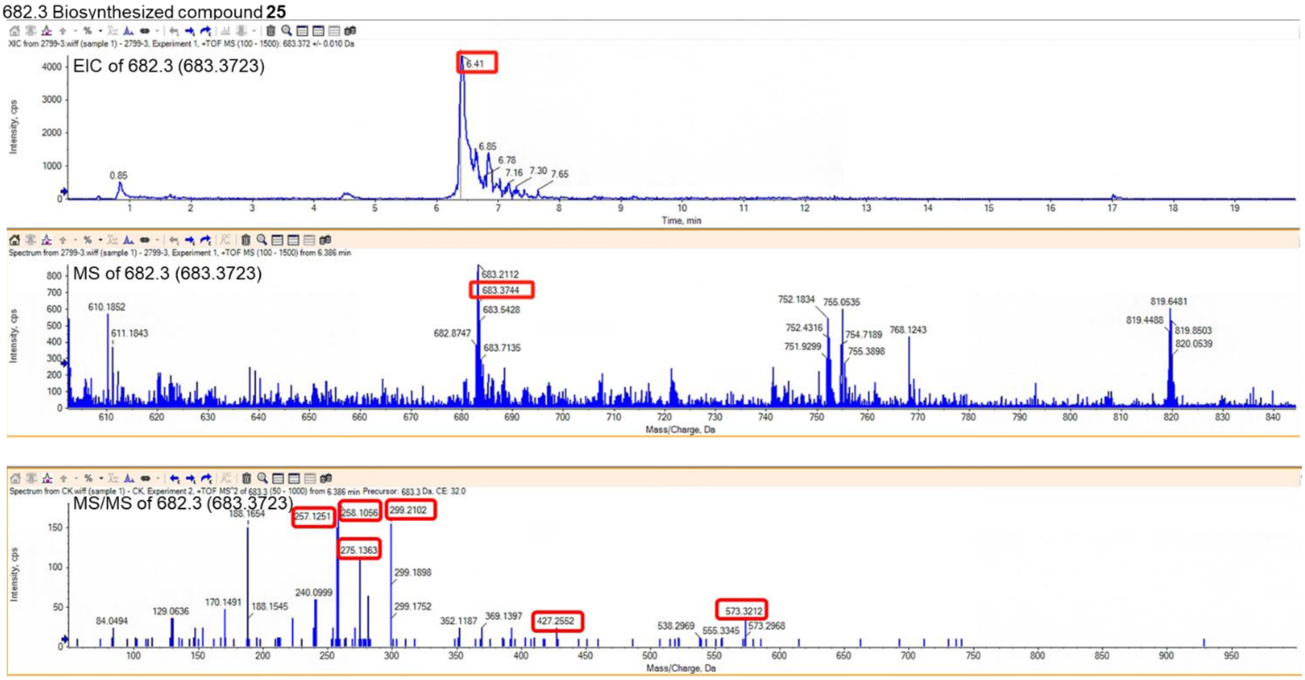

Figure S5. Biochemical reconstitution of the biosynthesis of *N*-acyl- tetraglutamine peptide (**23**) by purified Did A.

a, Reaction scheme for the DidA activity assay; **b**, Extracted ion chromatograms of the biosynthesized product the *N*-acyl-tetraglutamine peptide (**23**) obtained from the DidA activity assay; **c**, High resolution LC-MS/MS fragmentation of biosynthesized **23**.

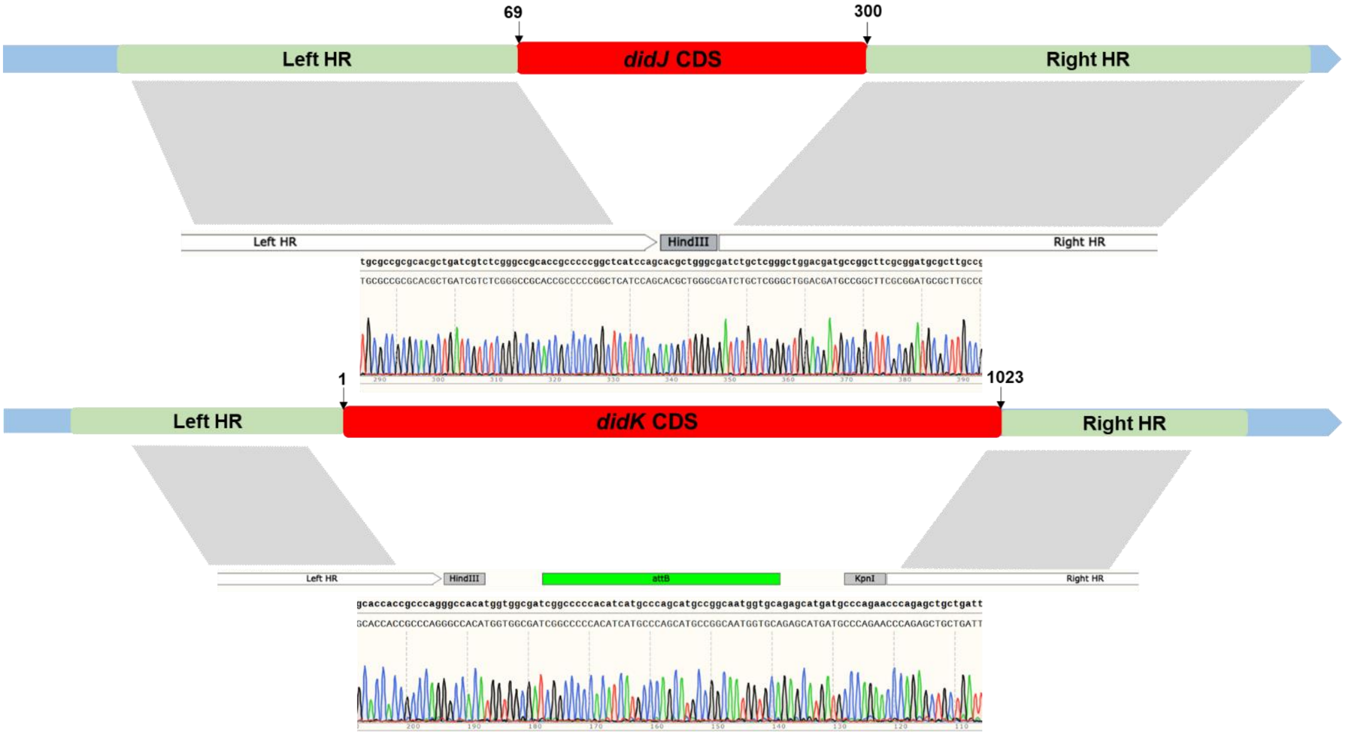

Figure S6. Verification of *T. mobilis/ΔdidJ* (69-300) and *T. mobilis/ΔdidK* (1-1023) mutants by Sanger sequencing.

The upper scheme shows the deletion of *didJ* from the base pair number 72 to 300. The bottom scheme reveals the deletion of *didK* from the base pair number 1 to 1023.

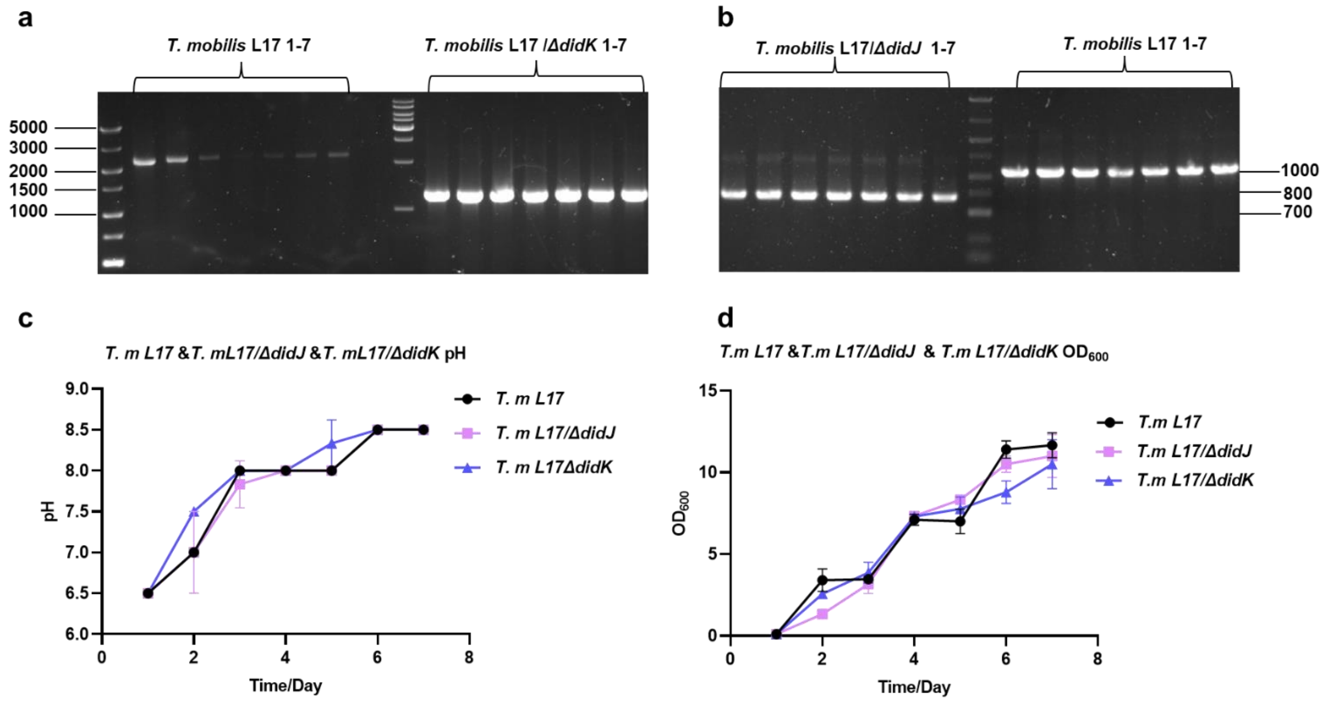

Figure S7. Verification of the knockout strains *T. mobilis* L17/*ΔdidJ* and *T. mobilis* L17/*ΔdidK.*

a, Seven monoclones were randonly selected for verification. PCR was performed using 60 °C annelaing step on a 30 cycle program using the primers g_didK-F and g_didK-R (Supporting table 1). 5 µL of reactions were loaded in a 1 % agarose gel and electrophoresed for 30 minutes at 120 V. 5000 represents the size of the ladder is 5000 bp, the same as other ladders. Image shown is a negative of the original gel image. The expected size of the PCR frangement in *T. mobilis* L17 is 2100 bp. Lanes 2-8 match the expected PCR profile. The expected size of the PCR frangement in *T. mobilis* L17/*ΔdidK* is 1100 bp. Lanes 11-18 match the expected PCR profile. **b,** Seven monoclones were randonly selected for verification. PCR was performed using 60 °C annelaing step on a 30 cycles program using the primers g_didJ-F and g_didJ-R (Supporting table 1). 5 µL of reactions were loaded in a 1 % agarose gel and electrophoresed for 30 minutes at 120 V. 1000 represents the size of the ladder is 1000 bp, the same as other ladders. The expected size of the PCR frangement in *T. mobilis* L17/*ΔdidJ* was 750 bp. Lanes 1-7 (counting from left to right) match the expected PCR profile. In the case of the wild-type *T. mobilis* L17 strain, the expected size of the PCR frangement is 1100 bp. Lanes 9-15 (counting from left to right) match the expected PCR profile. **c,** The figure shows the pH values of the wild type *T. mobilis* L17 strain, *T. mobilis* L17/*ΔdidJ* and *T. mobilis* L17/*ΔdidK* mutants during a 7-day fermetation. **d**, The figure shows the OD_{600} of the wild type *T. mobilis* L17 strain, *T. mobilis* L17/*ΔdidJ* and *T. mobilis* L17/*ΔdidK* mutants during a 7-day fermetation.

a The semi-preparative HPLC profile of the extract of *T. mobilis* L17/*ΔdidK* mutant

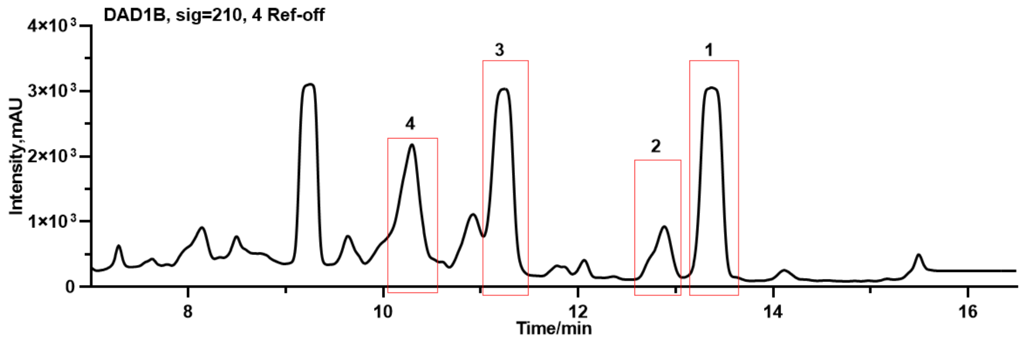

b The HPLC profile of DB and DX

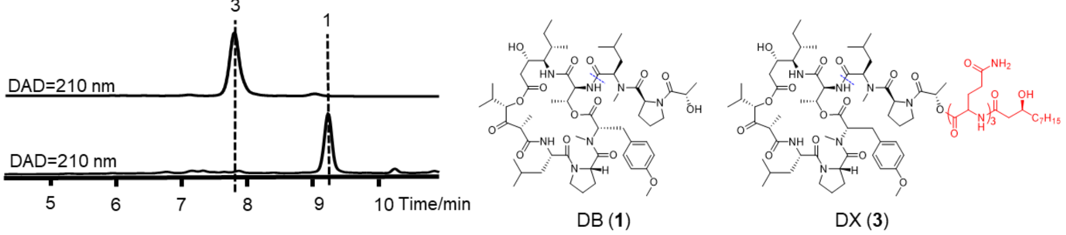

c The LC-ESI-HRMS and MS/MS analysis

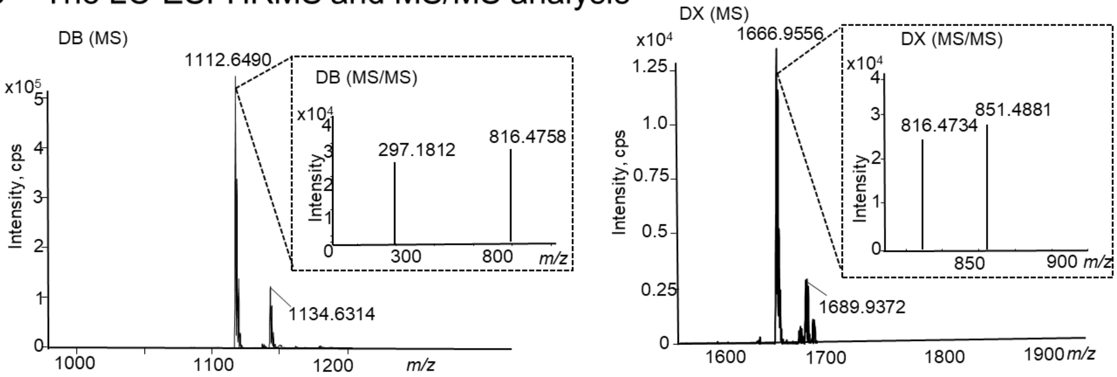

DB

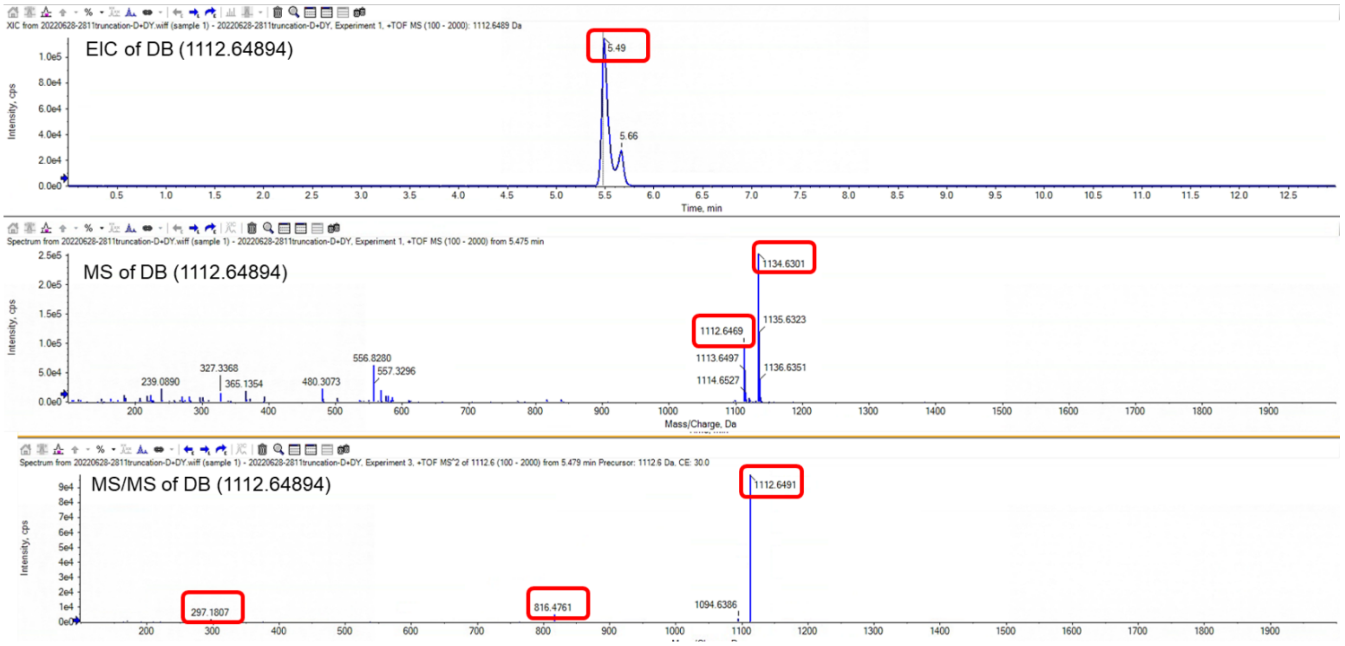

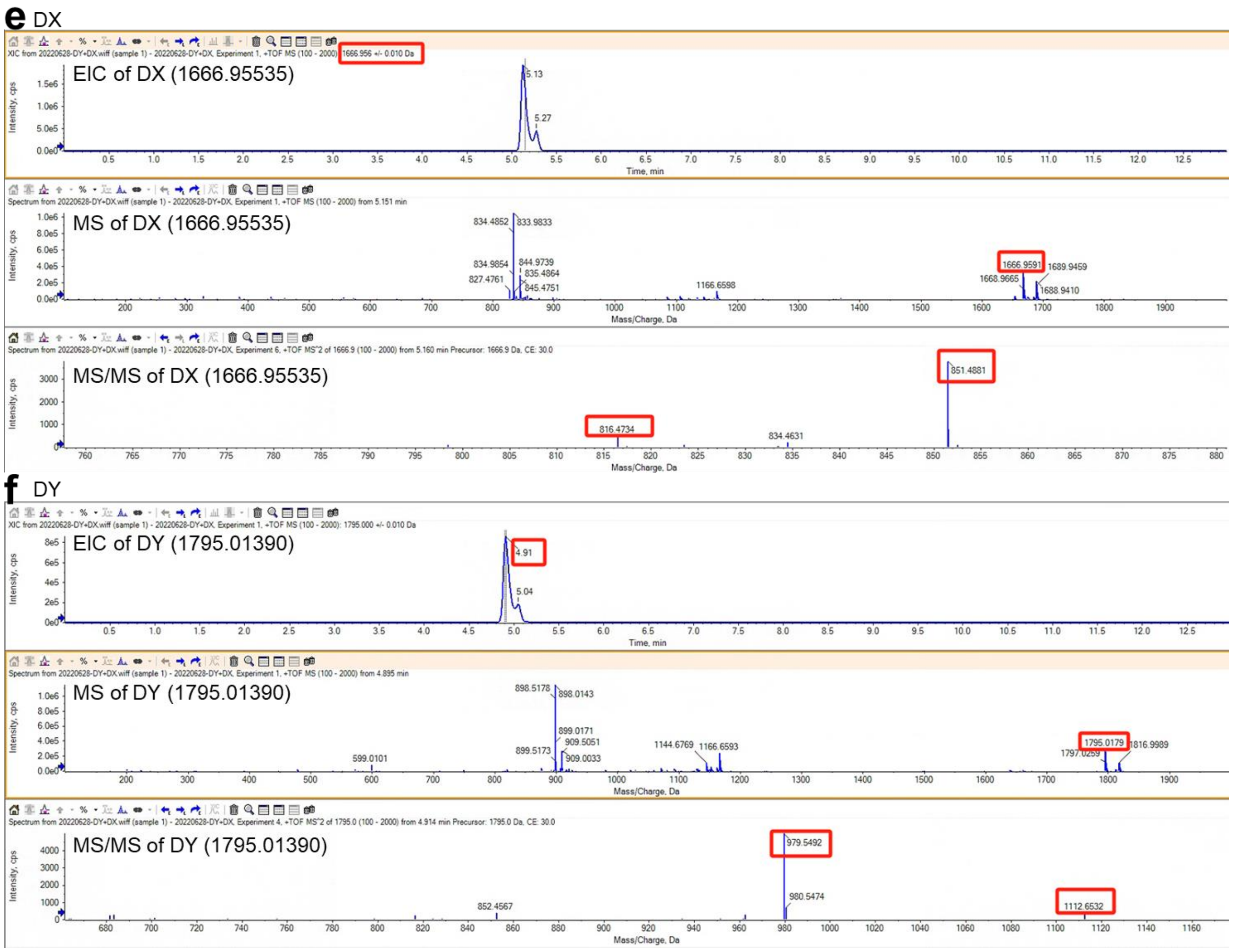

Figure S8. Isolation of didemnin B (**1**) and didemnin X (**3**)

a, The semi-preparative HPLC profile of the extract of *T. mobilis* L17/*ΔdidK* mutant using a Phenomenex Kinetex XB-C18 HPLC column (250 × 10 mm, 5 μm) at a flow rate of 2.8 mL/min. **b,** HPLC analysis of purified didemnin B (**1**) and didemnin X (**3**). **c, d, e and f,** LC-ESI-HRMS and MS/MS analysis of purified didemnin B (**1**), didemnin X (**3**) and didemnin Y (**4**).

NMR data

DB (1) ^{1}**H NMR** (400 MHz, $CDCl_3$) δ 7.81 (d, *J* = 9.2 Hz, 1H), 7.65 (d, *J* = 5.0 Hz, 1H), 7.22 (d, *J* = 9.3 Hz, 1H), 7.06 (d, *J* = 8.3 Hz, 2H), 6.83 (d, *J* = 8.3 Hz, 2H), 5.42 – 5.31 (m, 2H), 5.16 (d, *J* = 3.3 Hz, 1H), 4.80 (d, *J* = 10.1 Hz, 1H), 4.74 (t, *J* = 7.6 Hz, 1H), 4.65 – 4.60 (m, 1H), 4.54 (d, *J* = 3.1 Hz, 1H), 4.38 (q, *J* = 6.5 Hz, 1H), 4.22 (q, *J* = 6.8 Hz, 1H), 4.10 (dd, *J* = 8.4, 3.6 Hz, 1H), 4.08 – 4.01 (m, 2H), 3.78 (s, 3H), 3.72 – 3.64 (m, 2H), 3.62 – 3.53 (m, 3H), 3.37 (dd, *J* = 14.2, 3.8 Hz, 1H), 3.25 (d, *J* = 16.8 Hz, 1H), 3.18 (d, *J* = 11.3 Hz, 1H), 3.14 (s, 3H), 2.64 – 2.63 (m, 1H), 2.55 (s, 3H), 2.37 – 2.28 (m, 1H), 2.21 (s, 2H), 2.13 (t, *J* = 9.4 Hz, 3H), 2.05 – 2.00 (m, 1H), 1.97 (d, *J* = 6.5 Hz, 2H), 1.85 – 1.78 (m, 2H), 1.81 – 1.73

(m, 3H), 1.72 (dd, J = 14.3, 10.6 Hz, 2H), 1.69 – 1.62 (m, 1H), 1.59 (d, J = 12.6 Hz, 2H), 1.55 – 1.47 (m, 2H), 1.44 – 1.41 (m, 1H), 1.41 – 1.39 (m, 1H), 1.38 (d, J = 6.2 Hz, 3H), 1.36 (d, J = 5.0 Hz, 3H), 1.31 (d, J = 6.8 Hz, 3H), 1.22 – 1.16 (m, 2H), 0.91 – 0.84 (m, 24H).

DB (1) ^{13}**C NMR** (101 MHz, $CDCl_3$) δ 205.10, 174.04, 173.02, 172.55, 171.86, 171.36, 170.74, 169.82, 169.55, 168.57, 158.73, 130.43, 130.07, 114.24, 81.63, 70.56, 68.05, 66.56, 66.18, 57.75, 57.33, 56.86, 55.60, 55.40, 54.98, 49.69, 49.56, 47.19, 47.09, 41.42, 38.93, 38.81, 36.31, 34.15, 34.01, 31.39, 31.37, 28.51, 28.03, 27.25, 26.08, 25.15, 24.99, 24.96, 23.85, 23.49, 21.48, 21.05, 20.31, 18.73, 17.04, 16.39, 15.39, 14.81, 11.78.

NorDB (2) ^{1}**H NMR** ^{1}H NMR (600 MHz, $CDCl_3$) δ 7.78 (d, J = 9.1 Hz, 1H), 7.68 (d, J = 4.5 Hz, 1H), 7.31 (d, J = 9.9 Hz, 1H), 7.07 (d, J = 8.0 Hz, 2H), 6.84 (d, J = 7.7 Hz, 2H), 5.41 – 5.35 (m, 2H), 5.16 (s, 1H), 4.84 – 4.78 (m, 1H), 4.77 – 4.72 (m, 1H), 4.66 – 4.60 (m, 1H), 4.58 (s, 1H), 4.39 (dd, J = 12.8, 6.2 Hz, 2H), 4.24 (dd, J = 13.0, 6.4 Hz, 1H), 4.11 – 4.04 (m, 2H), 4.02 – 3.96 (m, 1H), 3.79 (s, 3H), 3.73 – 3.65 (m, 2H), 3.59 (dd, J = 17.6, 10.1 Hz, 3H), 3.38 (d, J = 14.3 Hz, 1H), 3.19 (d, J = 12.5 Hz, 1H), 3.14 (s, 3H), 3.14 – 3.05 (m, 1H), 2.68 (dd, J = 15.3, 11.6 Hz, 1H), 2.58 (s, 3H), 2.38 – 2.31 (m, 1H), 2.24 – 2.21 (m, 2H), 2.19 – 2.12 (m, 2H), 2.05 – 1.95 (m, 3H), 1.84 – 1.63 (m, 5H), 1.54 (d, J = 9.2 Hz, 1H), 1.41 (d, J = 6.3 Hz, 1H), 1.38 (d, J = 6.2 Hz, 4H), 1.32 (d, J = 6.4 Hz, 3H), 1.21 – 1.19 (m, 1H), 0.97 – 0.84 (m, 24H).

DX (3) ^{1}**H NMR** (400 MHz, $CDCl_3$) δ 8.23 (s, 1H), 8.09 (s, 1H), 7.97 (s, 1H), 7.89 (d, J = 8.7 Hz, 1H), 7.45 (d, J = 7.4 Hz, 1H), 7.08 (d, J = 8.4 Hz, 2H), 6.83 (d, J = 8.5 Hz, 2H), 6.59 (s, 2H), 5.30 (dd, J = 9.2, 5.4 Hz, 1H), 5.23 (d, J = 6.5 Hz, 1H), 5.13 (d, J = 3.6 Hz, 1H), 5.06 (d, J = 7.3 Hz, 1H), 4.87 (t, J = 6.2 Hz, 1H), 4.84 – 4.77 (m, 1H), 4.67 (d, J = 4.8 Hz, 1H), 4.62 – 4.56 (m, 1H), 4.50 (d, J = 5.3 Hz, 1H), 4.40 (s, 2H), 4.12 (q, J = 6.5 Hz, 1H), 4.02 (s, 2H), 3.97 (s, 1H), 3.78 (s, 3H), 3.70 (s, 1H), 3.66 – 3.54 (m, 3H), 3.33 (d, J = 10.8 Hz, 1H), 3.20 (s, 1H), 3.09 (s, 1H), 3.05 (s, 3H), 2.65 – 2.57 (m, 1H), 2.53 (s, 3H), 2.44 (d, J = 13.6 Hz, 1H), 2.37 – 2.28 (m, 8H), 2.17 – 2.08 (m, 11H), 1.85 (dd, J = 12.2, 6.0 Hz, 1H), 1.79 – 1.73 (m, 1H), 1.68 – 1.53 (m, 3H), 1.47 (d, J = 6.4 Hz, 1H), 1.34 (d, J = 7.0 Hz, 3H), 1.33 (d, J = 7.0 Hz, 3H), 1.29 – 1.21 (m, 11H), 1.19 – 1.11 (m, 1H), 0.91 – 0.84 (m, 27H).

DX (3) ^{13}**C NMR** (101 MHz, $CDCl_3$) δ 204.77, 176.64, 176.43, 176.18, 173.45, 173.28, 172.57, 172.26, 172.17, 172.08, 171.63, 171.28, 170.56, 170.21, 169.78, 169.31, 168.59, 158.79, 130.52, 129.86, 114.27, 81.75, 70.90, 69.50, 69.06, 67.82, 66.26, 57.39, 57.12, 56.95, 56.04, 55.41, 54.38, 53.33, 53.12, 52.03, 49.71, 49.65, 47.33, 47.21, 43.67, 41.53, 38.83, 38.70, 37.62, 36.47, 34.34, 34.04, 32.01, 31.17, 31.03, 29.78, 29.47, 28.77, 28.05, 27.70, 27.02, 25.90, 25.50, 25.17, 24.99, 24.70, 23.99, 23.59, 22.80, 21.55, 21.06, 18.77, 17.14, 16.11, 16.00, 15.46, 14.97, 14.27, 11.79.

DY (4) ^{1}**H NMR** (600 MHz, DMSO-d6) δ 8.67 (d, J = 7.3 Hz, 1H), 8.37 (d, J = 7.2 Hz, 1H), 8.31 (s, 2H), 8.06 – 7.92 (m, 4H), 7.89 (d, J = 9.2 Hz, 1H), 7.72 (dd, J = 17.7, 7.7 Hz, 2H), 7.22 (d, J = 18.8 Hz, 5H),

7.15 (dd, J = 8.6, 3.4 Hz, 2H), 6.95 (d, J = 7.9 Hz, 1H), 6.89 – 6.85 (m, 2H), 6.77 (s, 3H), 5.46 – 5.12 (m, 6H), 5.05 (d, J = 5.7 Hz, 1H), 4.98 – 4.95 (m, 1H), 4.91 (dd, J = 5.8, 3.1 Hz, 2H), 4.88 – 4.76 (m, 3H), 4.67 – 4.53 (m, 4H), 4.48 (dd, J = 7.4, 2.7 Hz, 1H), 4.40 (dd, J = 6.6, 2.5 Hz, 1H), 4.27 – 4.17 (m, 5H), 4.10 – 4.00 (m, 2H), 4.00 – 3.88 (m, 3H), 3.78 (q, J = 9.9, 6.9 Hz, 4H), 3.73 (s, 3H), 3.69 – 3.59 (m, 5H), 3.55 – 3.45 (m, 3H), 3.20 – 3.15 (m, 2H), 3.04 (s, 2H), 3.01 – 2.94 (m, 2H), 2.83 (s, 1H), 2.23 – 2.05 (m, 14H), 2.04 – 1.84 (m, 14H), 1.83 – 1.66 (m, 7H), 1.62 – 1.43 (m, 9H), 1.40 – 1.07 (m, 51H), 1.00 – 0.67 (m, 27H).

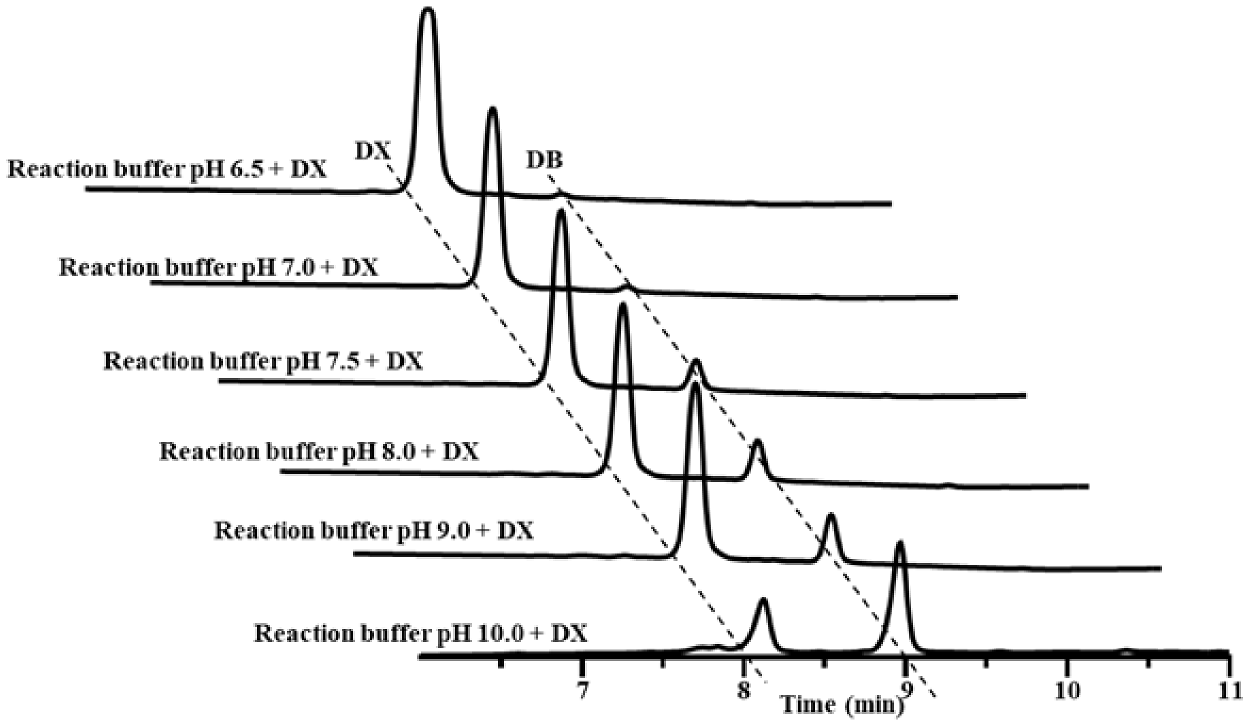

Figure S9. The stability of didemnin X in reaction buffer across a pH range from pH 6.5 to 10.

A 2.5 µl aliquot of didemnin X stock in DMSO was incubated with 300 µL of reaction buffer (PBS buffer, pH 7.0 to pH 10.0) at 37 ºC overnight. Didemnin X was extracted with equal ethyl acetate twice and redissolved in acetonitrile for HPLC analysis. As determined by HPLC, the retention times for didemnin X and didemnin B were approximately 8.1 minutes and 9.0 minutes, respectively.

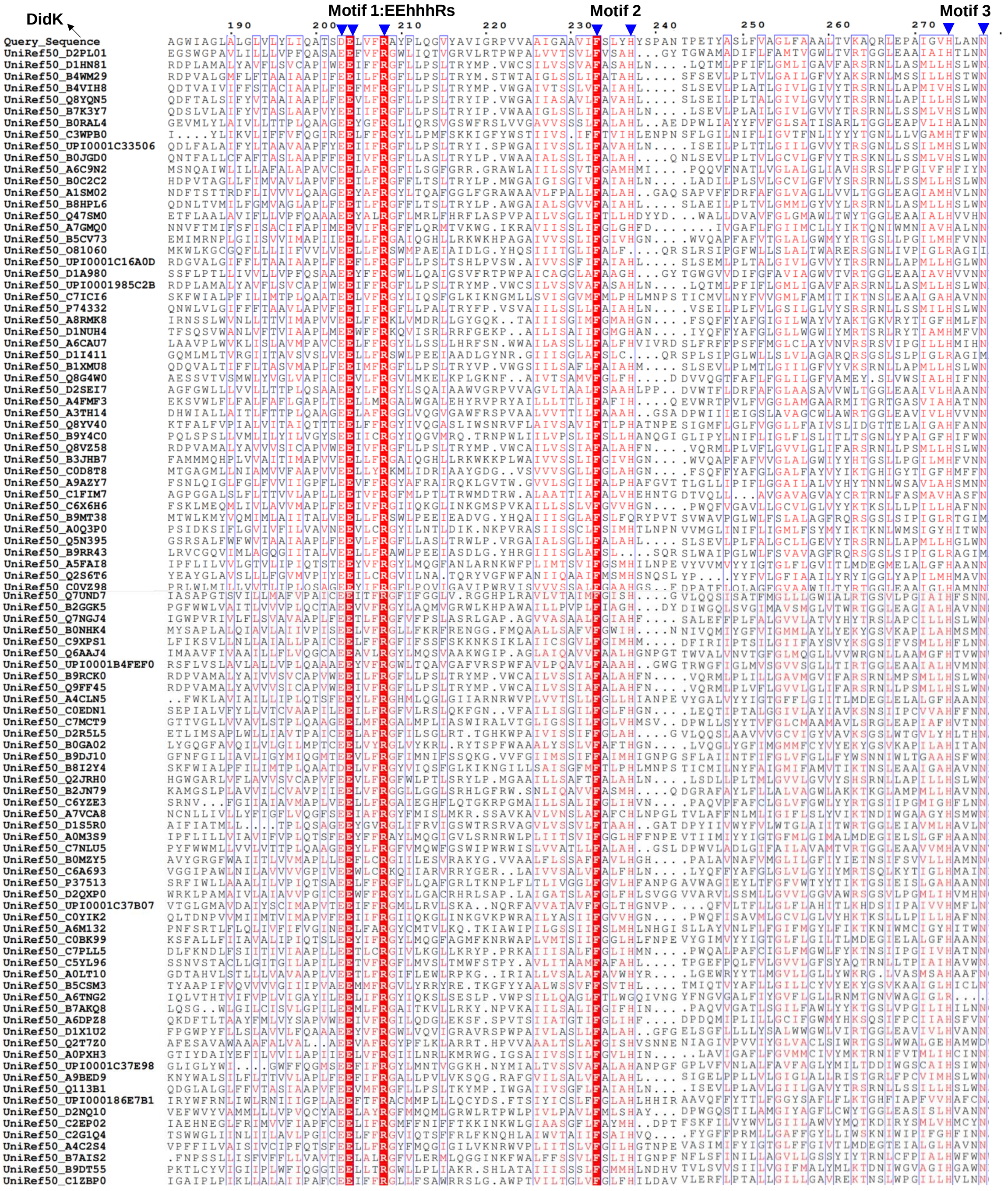

Figure S10. Alignment of DidK (top) and some representative homologs

These sequences can be found by their uniprot ID in uniprot website (https://www.uniprot.org/). The letters are highlighted with the red background are conserved amino acids. The alignment shows these sequences have three motifs. Motif 1 is EEhhhRs, where h and s are hydrophobic and small residues. Putative zinc-binding residues. Motifs 2 and 3 both contain one semi-invariant His residue. Motif 2 begins with a conserved aromatic residue and motif 3 ends with a sequence enriched in Asn or Asp residues. Triangles represent the predicted catalytic residues.

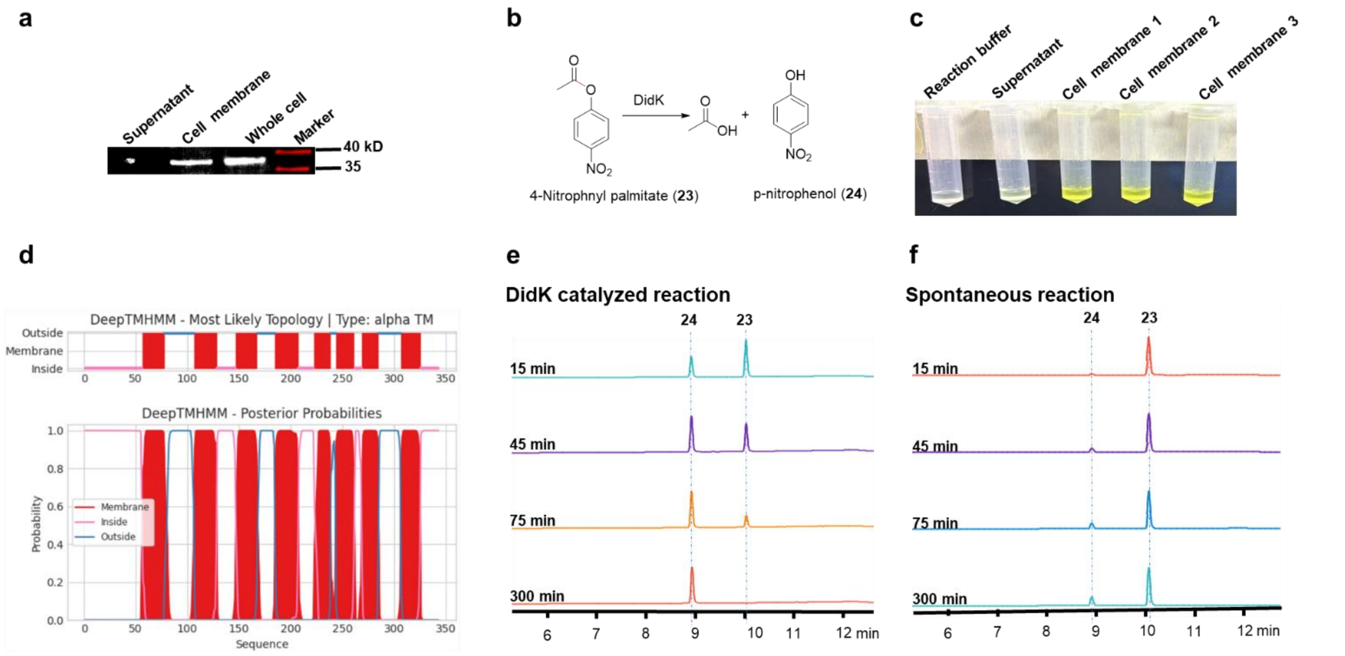

Figure S11. DidK is an intramembrane protein with predicted eight transmembrane α-helices.

a, Confirmtion of DidK is located in cell membrane by Western Blot. The lane 1 (counting from left to right) represents the supernatant of cell lysate of *E.coli* BL21(DE)$_3$/pCold-TF::*didK*. The lane 2 represents the membrane fraction of cell lysate of *E.coli* BL21(DE)$_3$/pCold-TF::*didK*. The lane 3 represents the whole cell lysate of *E.coli* BL21(DE)$_3$/pCold-TF::*didK*. The lane 4 are protein markers. **b,** DidK hydrolyzes the compound **23** into the compound **24**. **c,** Tube 1 represents the reaction buffer. Tube 2 represents the supernatant of *E.coli* BL21(DE)$_3$/pCold-TF::*didK*. Tubes 3, 4 and 5 represent the reactions using the cell memberane fragement as the catalysts. Compound **24** is yellow in water, while compound **23** is colorless in water. **d,** The prediction of the membrane topology of DidK (https://dtu.biolib.com/DeepTMHMM)[7]. **e,** The HPLC analysis of the process in hydrolyzing compound **23** by DidK in a time course ranging from 15 min to 300 min. **f,** The HPLC analysis of the spontaneous reaction of compound **23** (no DidK) in a time course ranging from 15 min to 300 min.

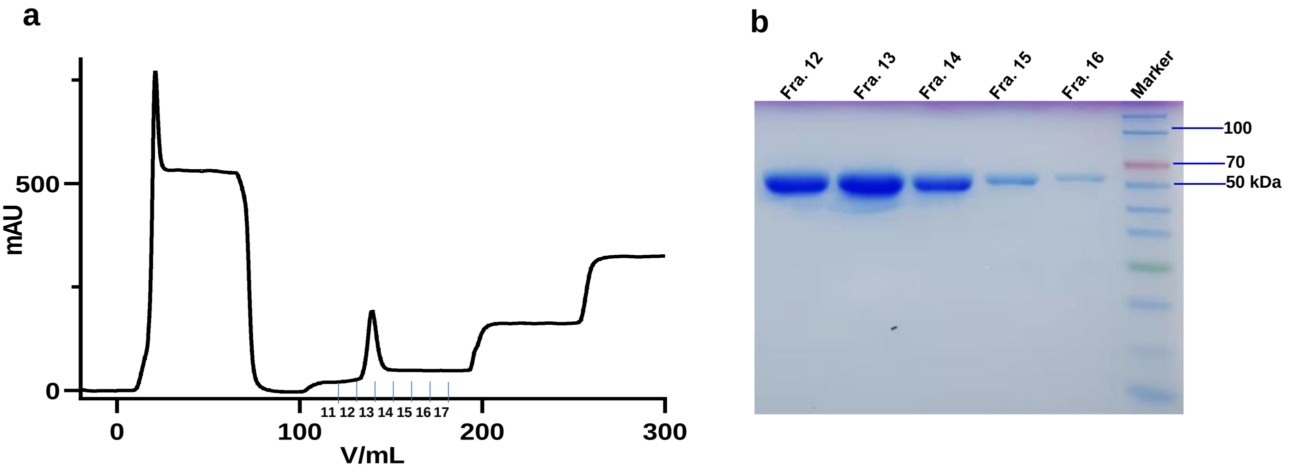

Figure S12. SDS-PAGE analysis of the purification of DidK.

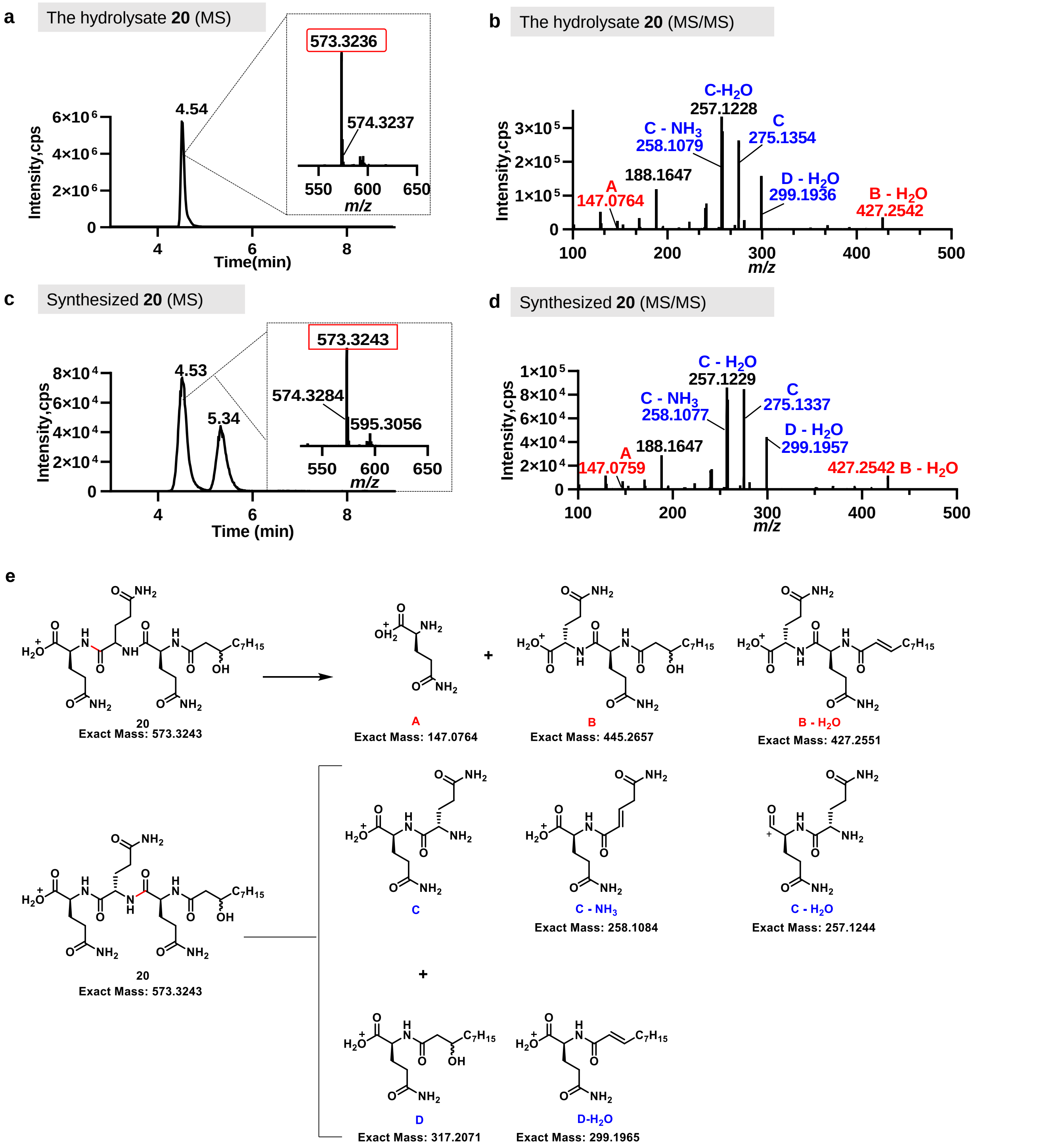

Figure S13. LC-HRMS/MS analysis of the fragments of the hydrolysate (**20**) from the reaction of DidK when using didemnin X as the substrate.

a, b, LC-HRMS/MS analysis of the hydrolysate **20** from the reaction of DidK using didemnin X as the substrate. **c**, **d**, LC-HRMS/MS analysis of the synthetic **20**. **e**, The proposed MS/MS fragmentations of compound **20**.

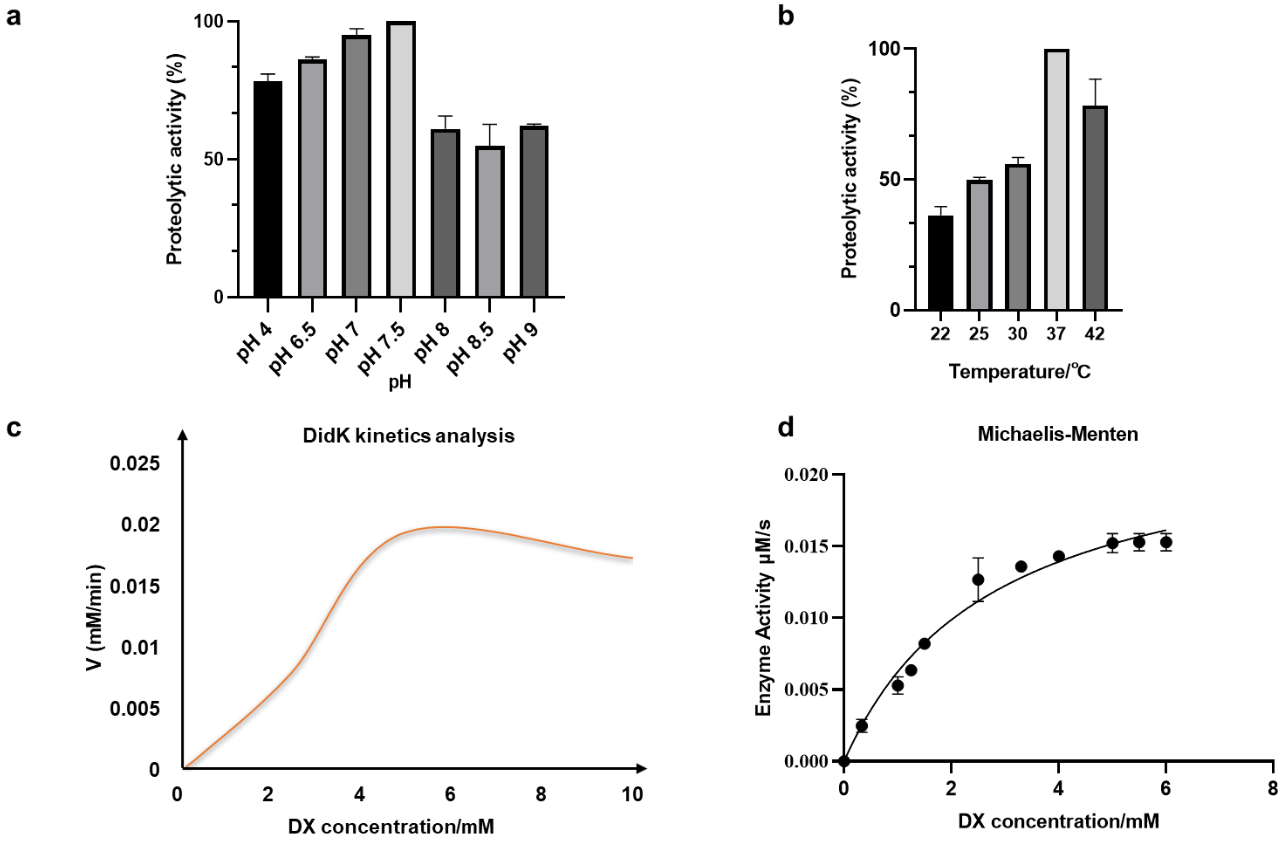

Figure S14. Determination of the optimum reaction temperature, pH value, the maximum substrate concentration, and Michaelis–Menten kinetics for DidK.

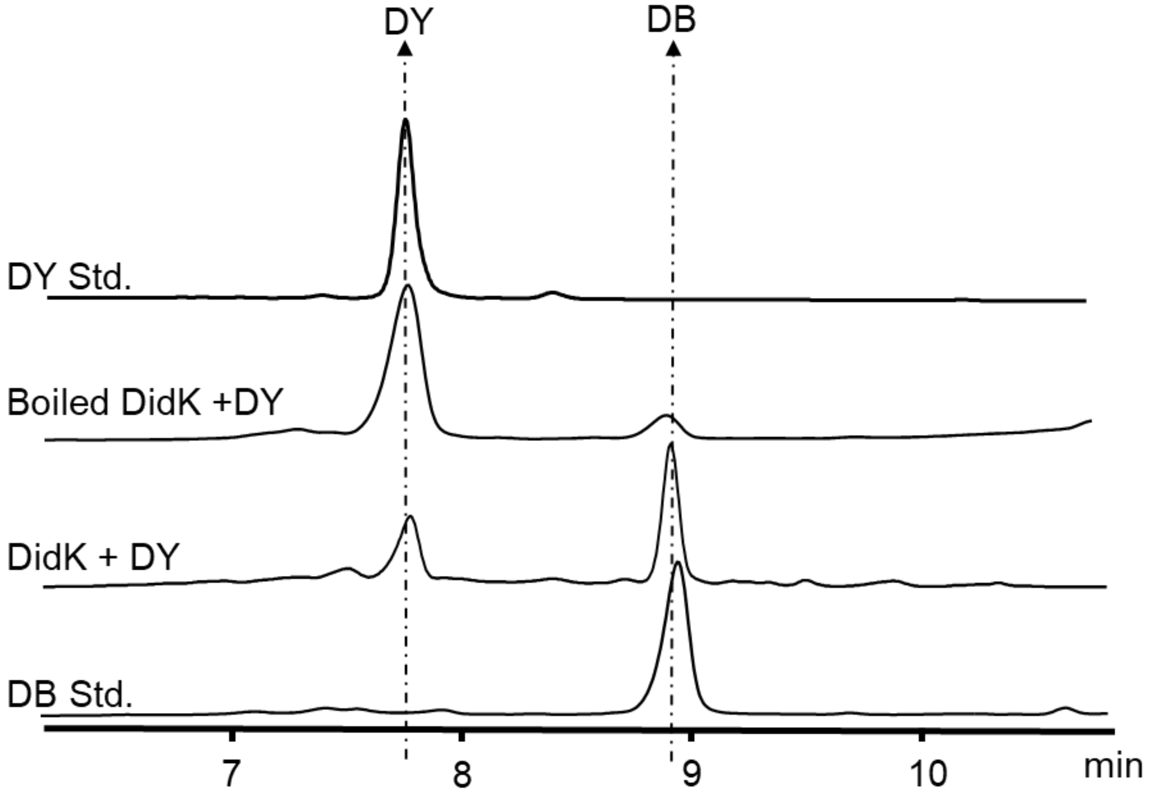

Figure S15. DidK is required for cleaving didemnin Y.

HPLC analysis of didemnin B (**1**, std.) and didemnin Y (**4**, std.) standards and the DidK activity assay with boiled DidK, and the DidK activity assay for 12 h. As determined by HPLC, the retention times for didemnin Y and didemnin B were approximately 7.8 minutes and 9.0 minutes, respectively.

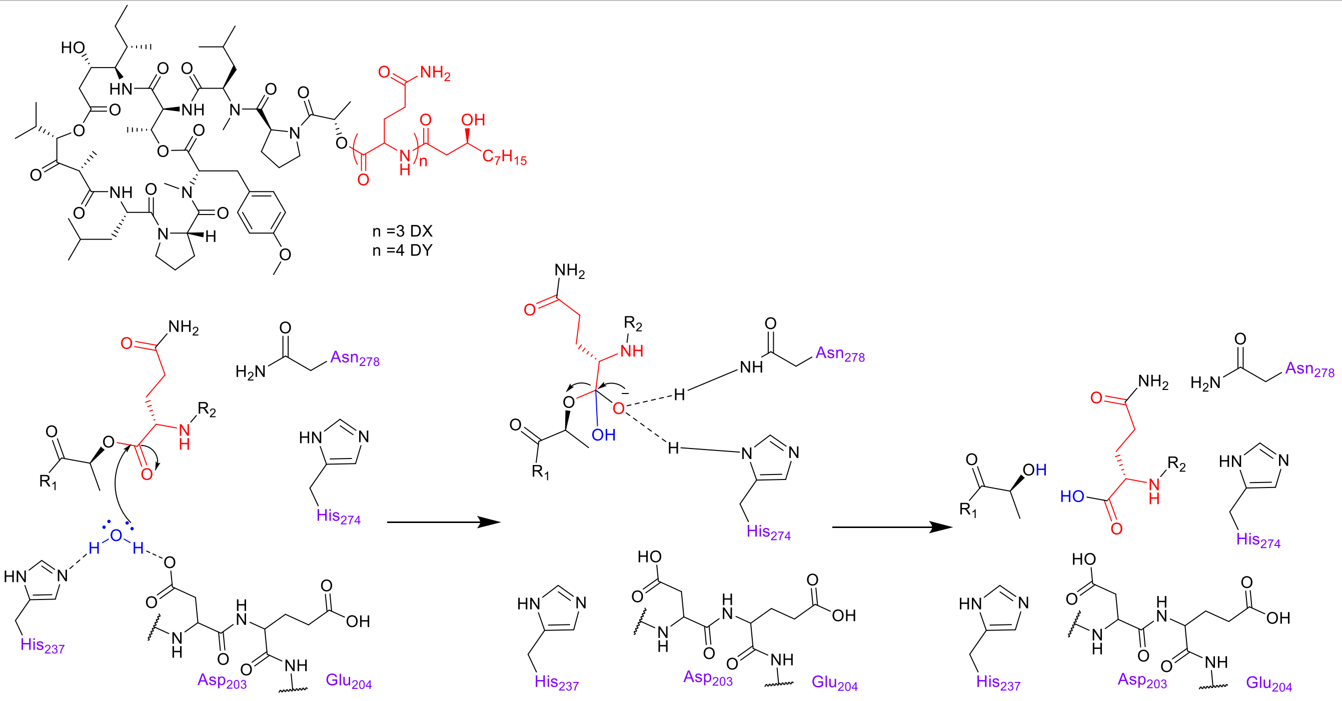

Figure S16. A plausible catalytic mechanism of DidK.

A plausible catalytic mechanism might entail the involvement of D203, E204, and H237 for general base-catalyzed deprotonation of a water molecule, facilitating its nucleophilic attack on the scissile bond of the substrate. The side chains of H274 and N278, situated within the catalytic dyad formed by D203 and H237, are likely to contribute hydrogen bonds to stabilize the oxyanion transition state. Protonation of the departing amino group in the AAX tripeptide could potentially be catalyzed by either D203 or H237.

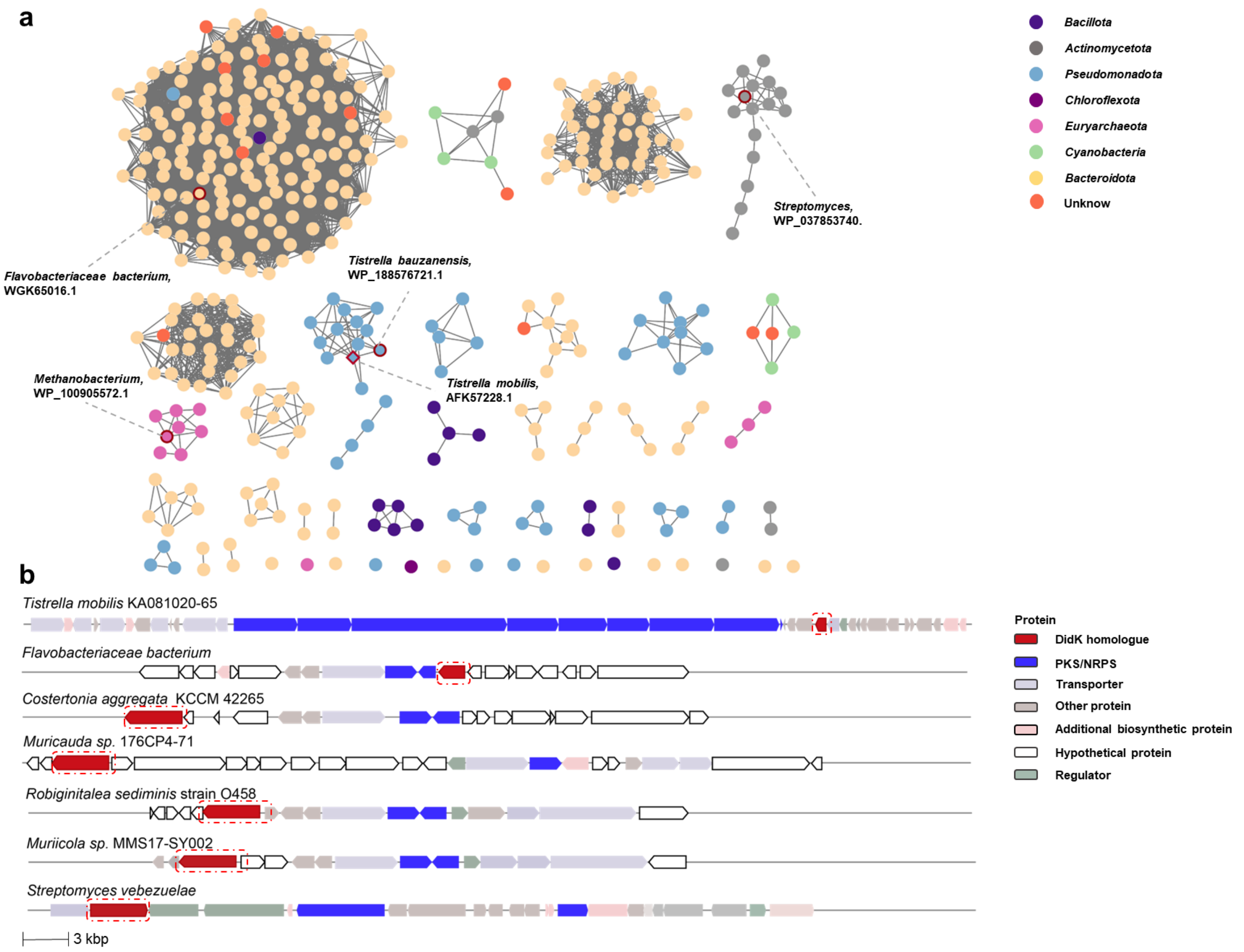

Figure S17. Distribution of DidK homologues in various bacterial genomes

a, Distribution of DidK homologues across verious bacterial genome. The sequence similarity network (SSN) analysis of the DidK homologues in diverse organisms was visualized using Cytoscape[19], with DidK homologs color-coded according to their bacterial phyla. **b,** Genome Neighborhood Diagram featuring examples of DidK homologs adjacent to bacterial NRPS or PKS. Proteins containing DidK homolougues are highlighted by red, while NRPS/PKS domains are marked in blue within the diagram.

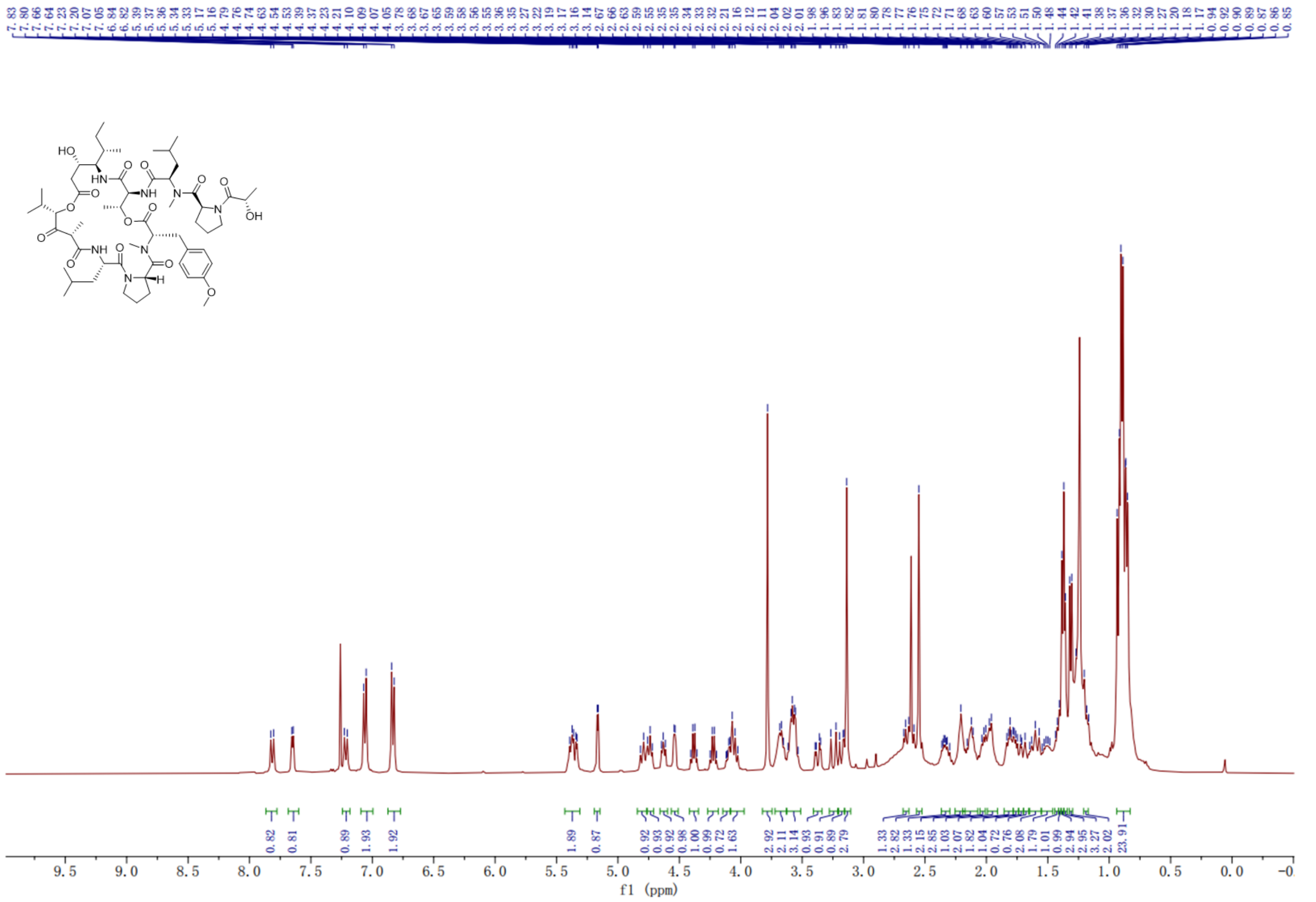

Figure S18. ^{1}H NMR spectrum of purified didemnin B (**1**, 400 MHz). Solvent: $CDCl_3$

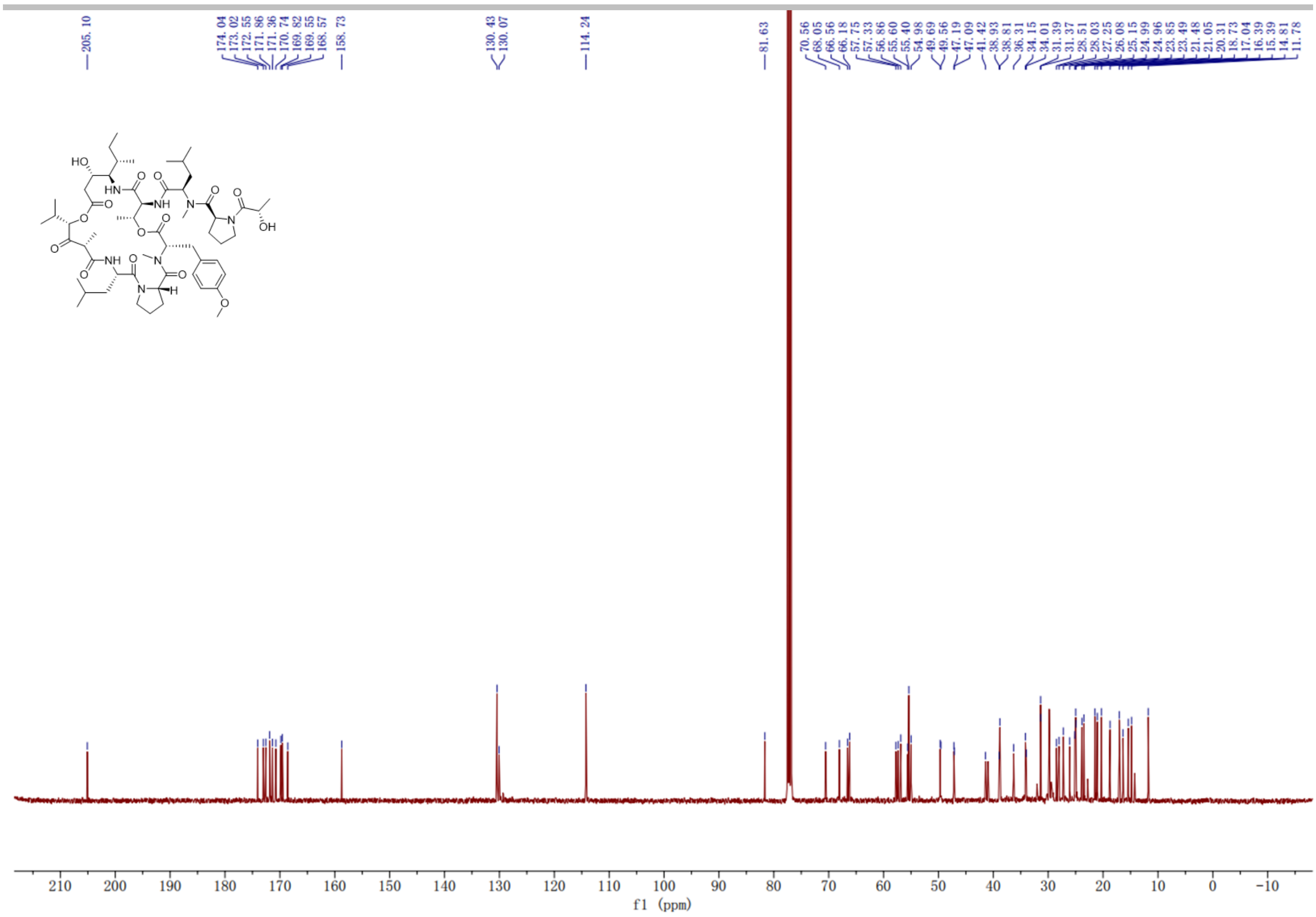

Figure S19. ^{13}C NMR spectrum of purified didemnin B (**1**, 100 MHz). Solvent: $CDCl_3$

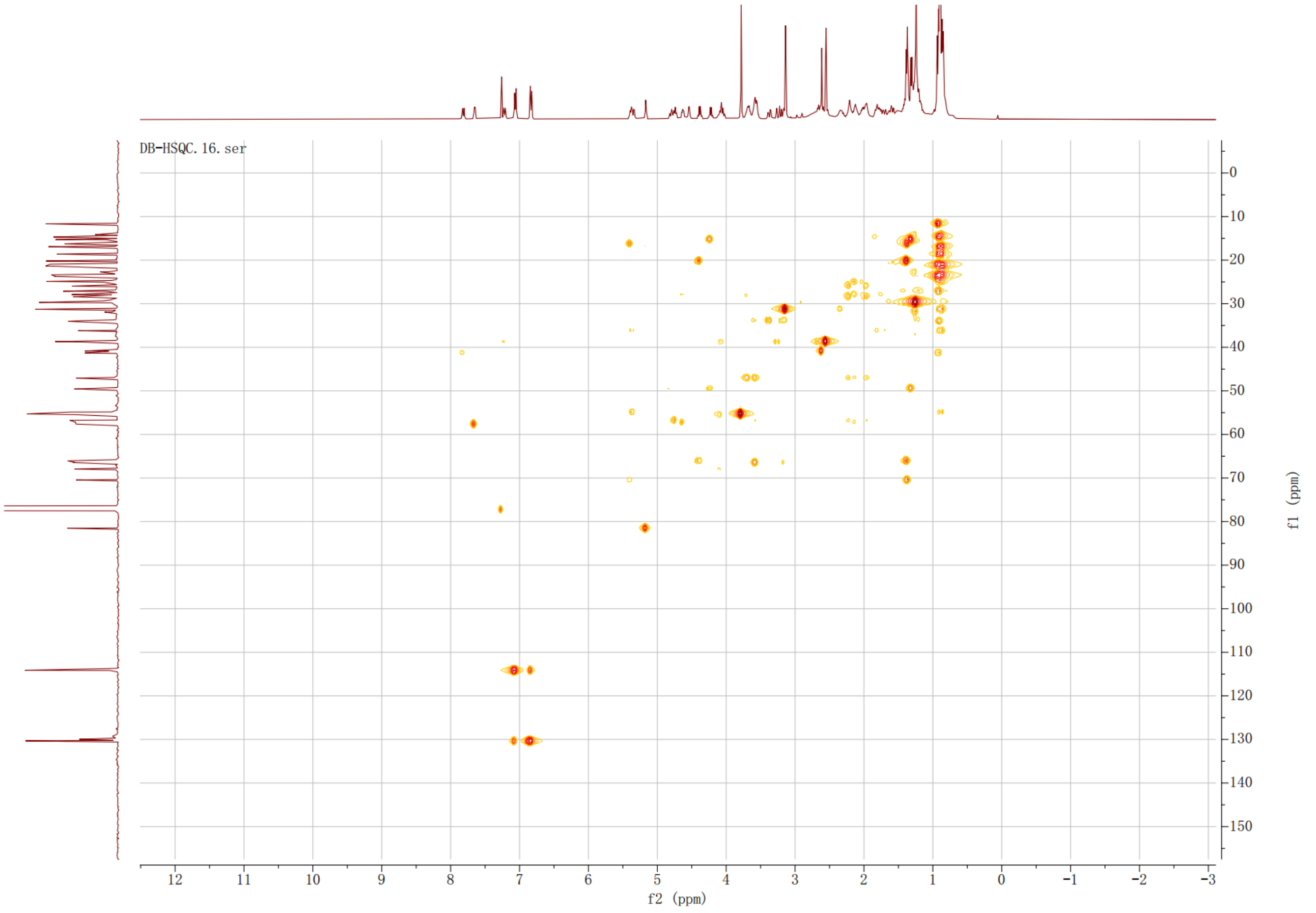

Figure S20. ^{1}H-^{13}C-HSQC spectrum of purified didemnin B (**1**). Solvent: $CDCl_3$.

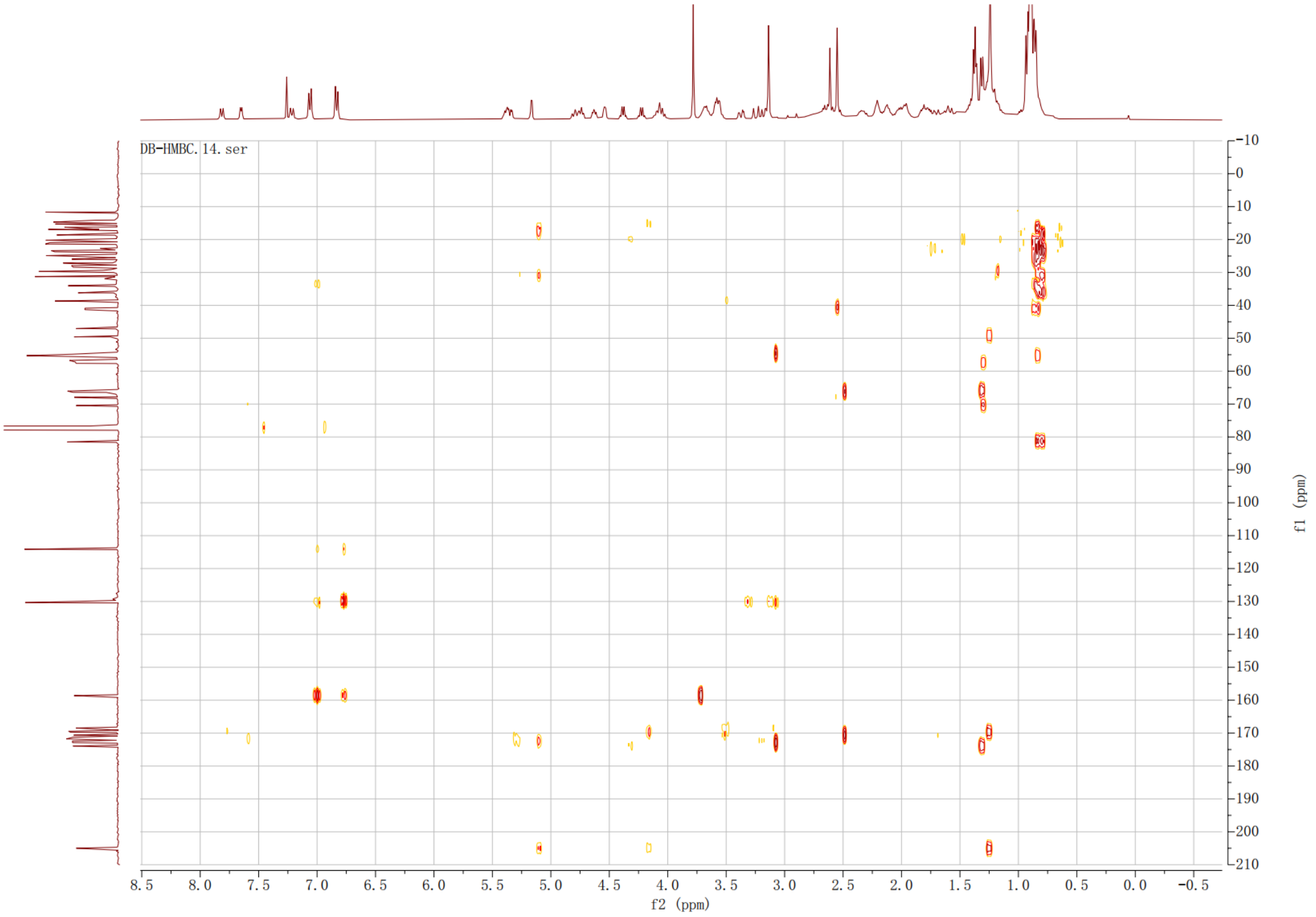

Figure S21. ^{1}H-^{13}C-HMBC spectrum of purified didemnin B (**1**). Solvent: $CDCl_3$.

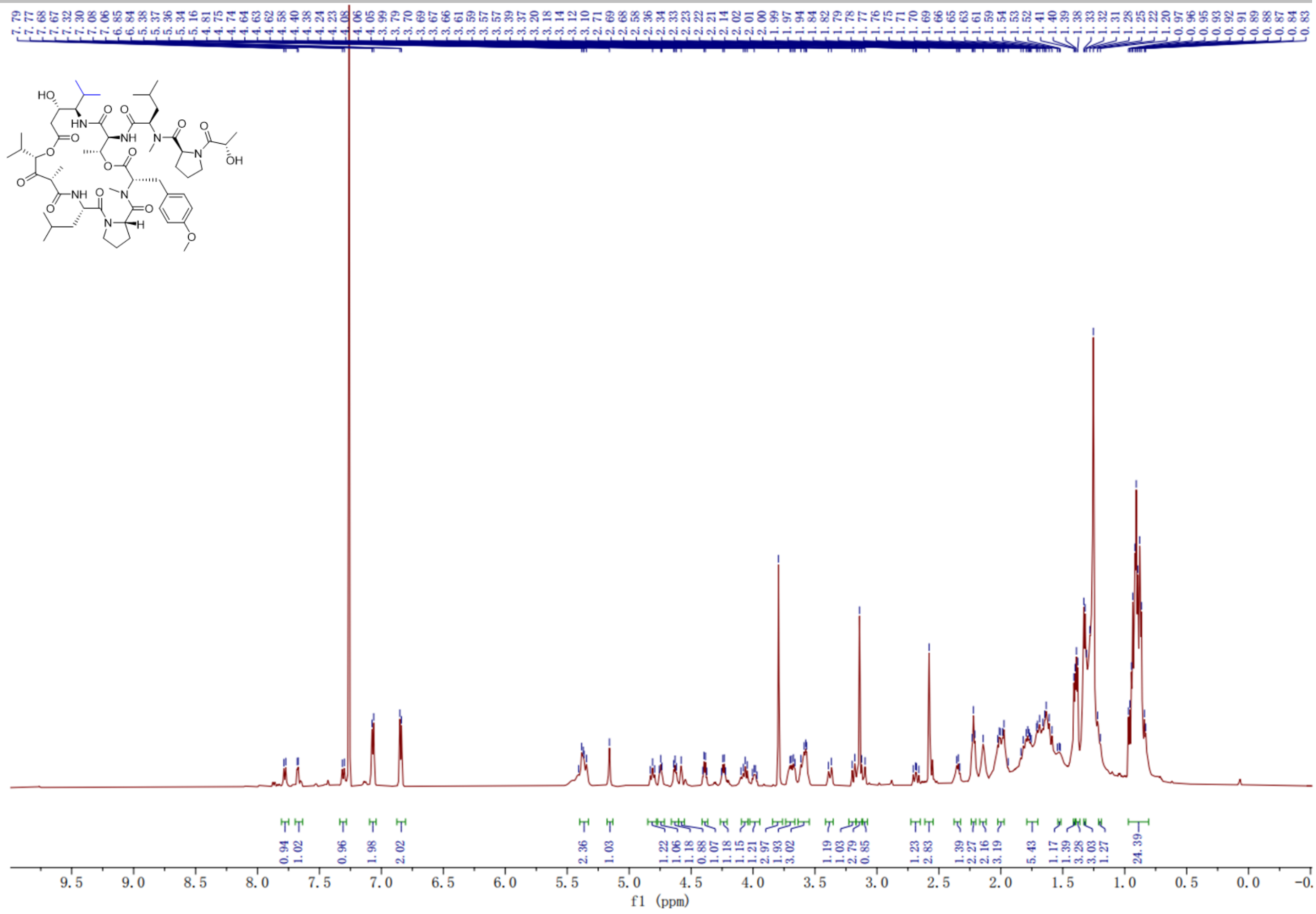

Figure S22. ^{1}H NMR spectrum of purified nordidemnin B (**2**, 400 MHz). Solvent: $CDCl_3$

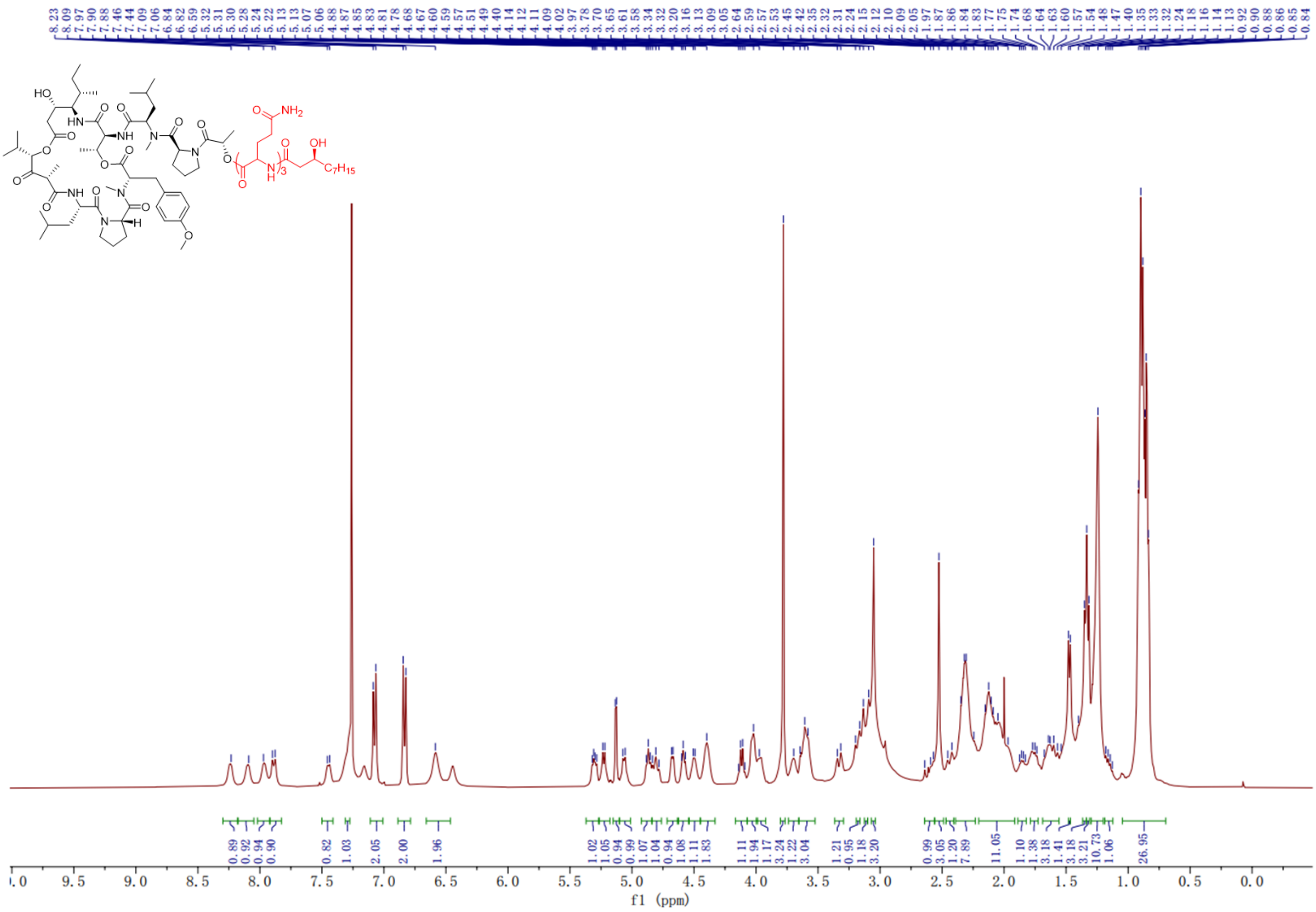

Figure S23. ^{1}H NMR spectrum of purified didemnin X (**3**, 400 MHz). Solvent: $CDCl_3$

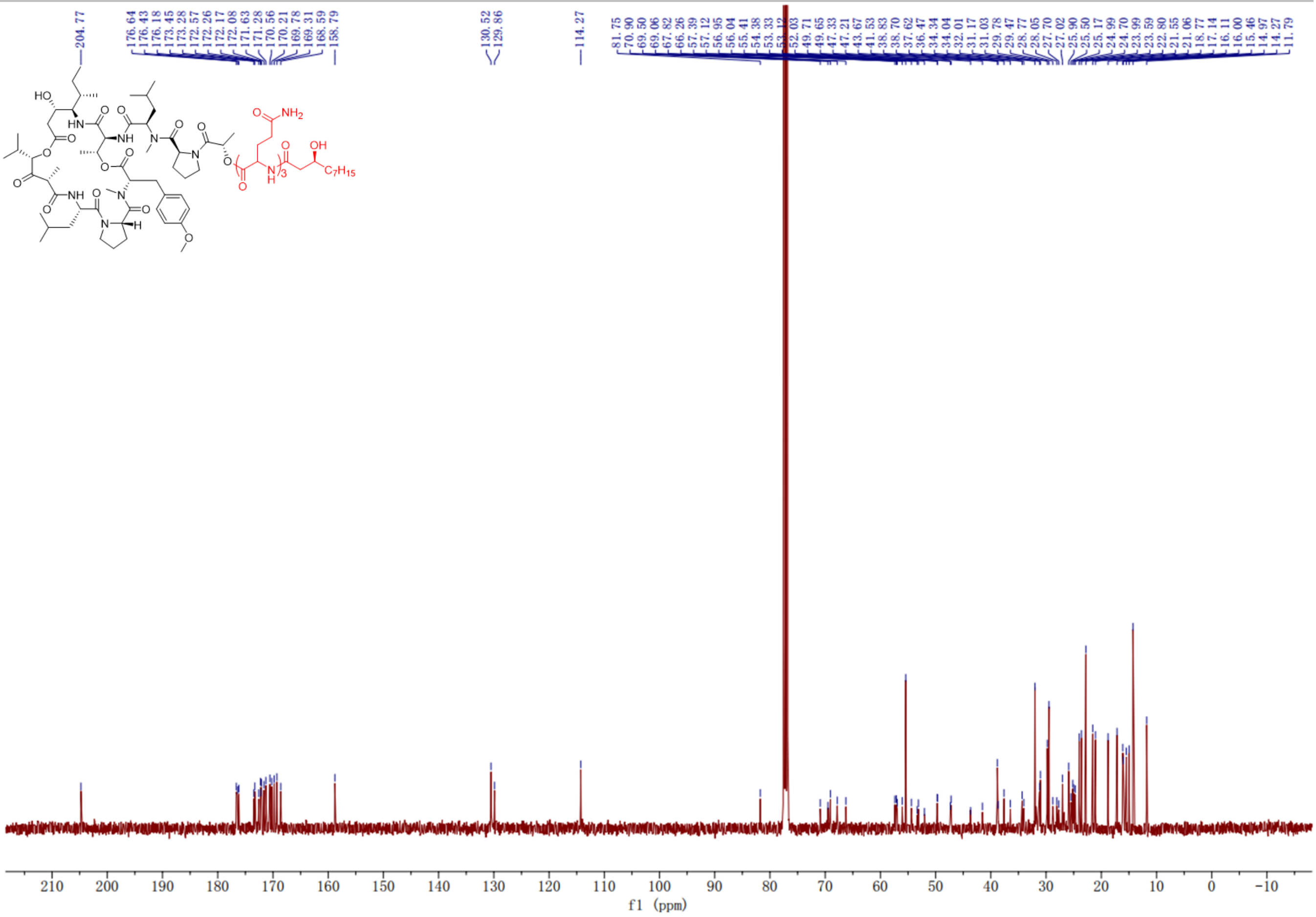

Figure S24. ^{13}C NMR spectrum of purified didemnin X (**3**), 101 MHz. Solvent: $CDCl_3$

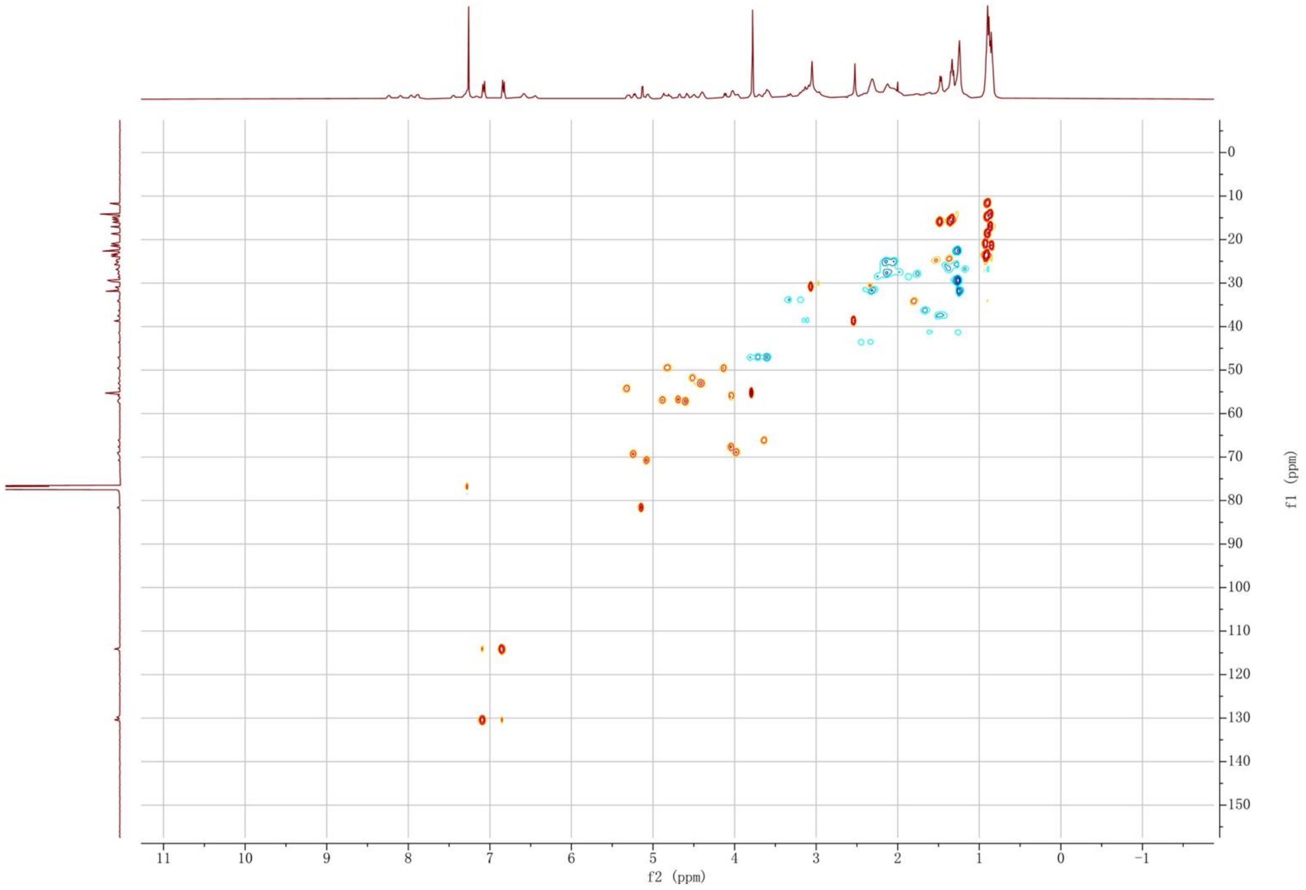

Figure S25. ^{1}H-^{13}C-HSQC spectrum of purified didemnin X (**3**). Solvent: $CDCl_3$.

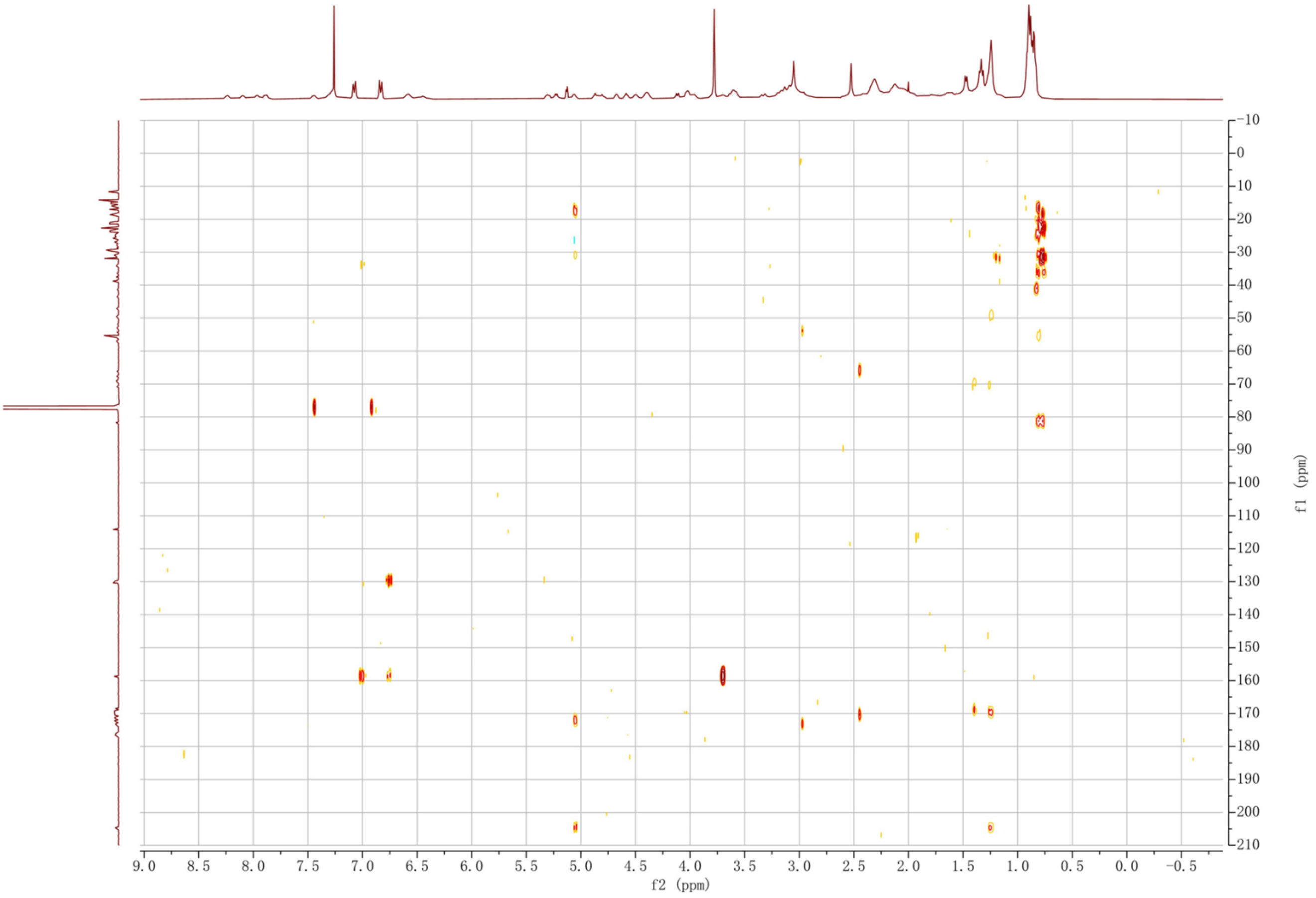

Figure S26. ^{1}H-^{13}C-HMBC spectrum of purified didemnin X (**3**). Solvent: $CDCl_3$.

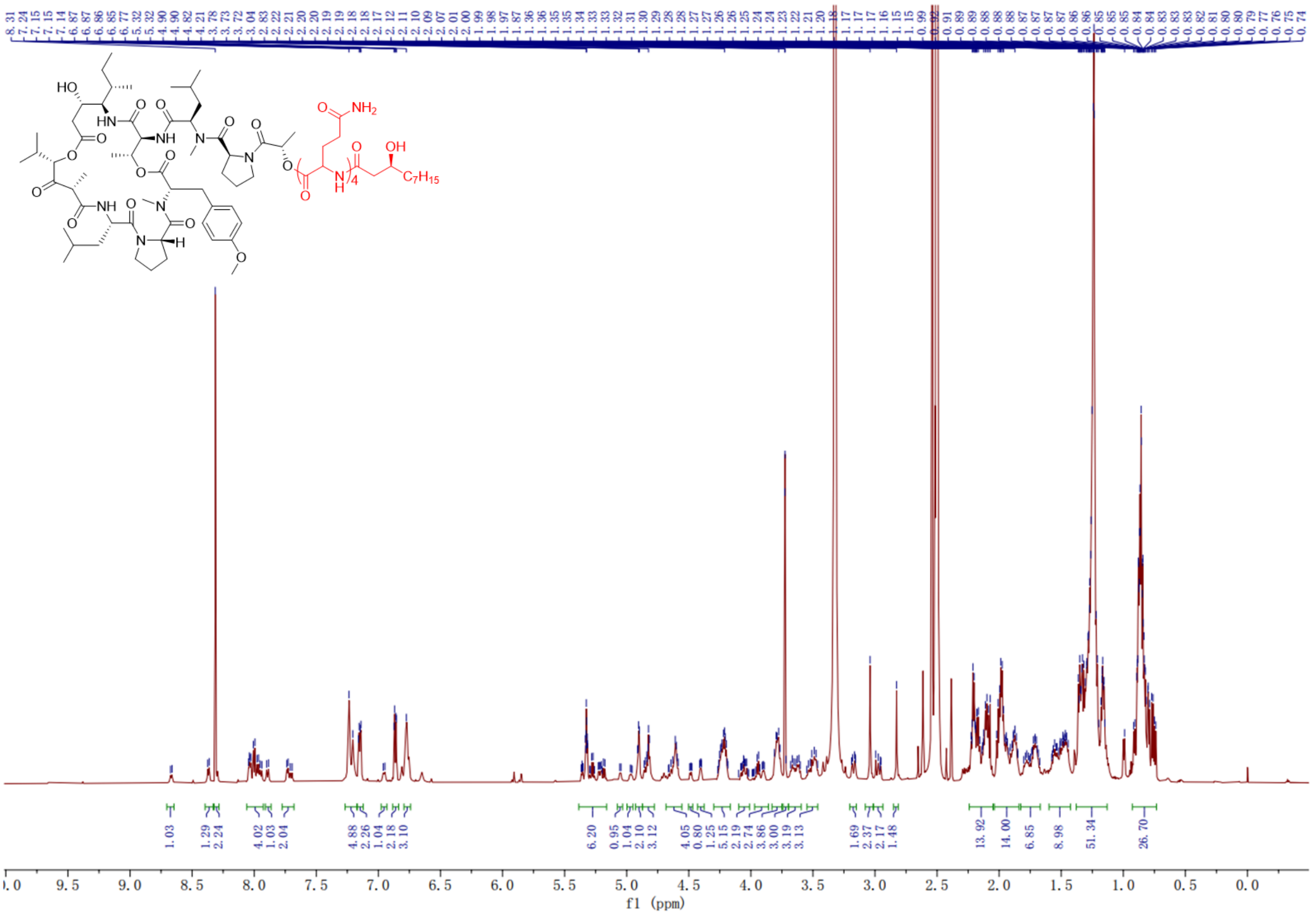

Figure S27. ^{1}H NMR spectrum of purified didemnin Y (**4**, 600 MHz). Solvent: DMSO-*d6*

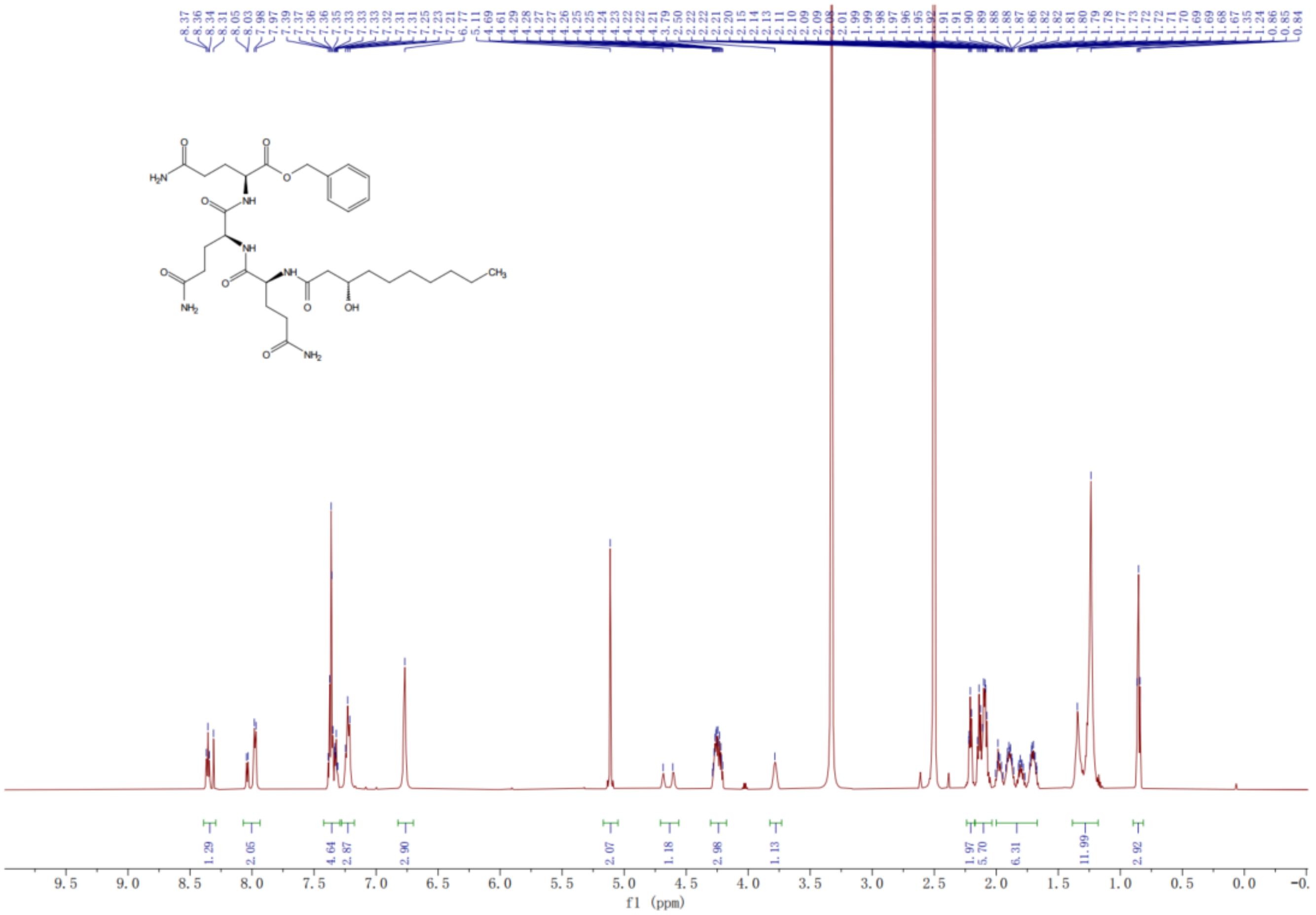

Figure S28. ^{1}H NMR spectrum of compound **19** (600 MHz). Solvent: DMSO-*d6*

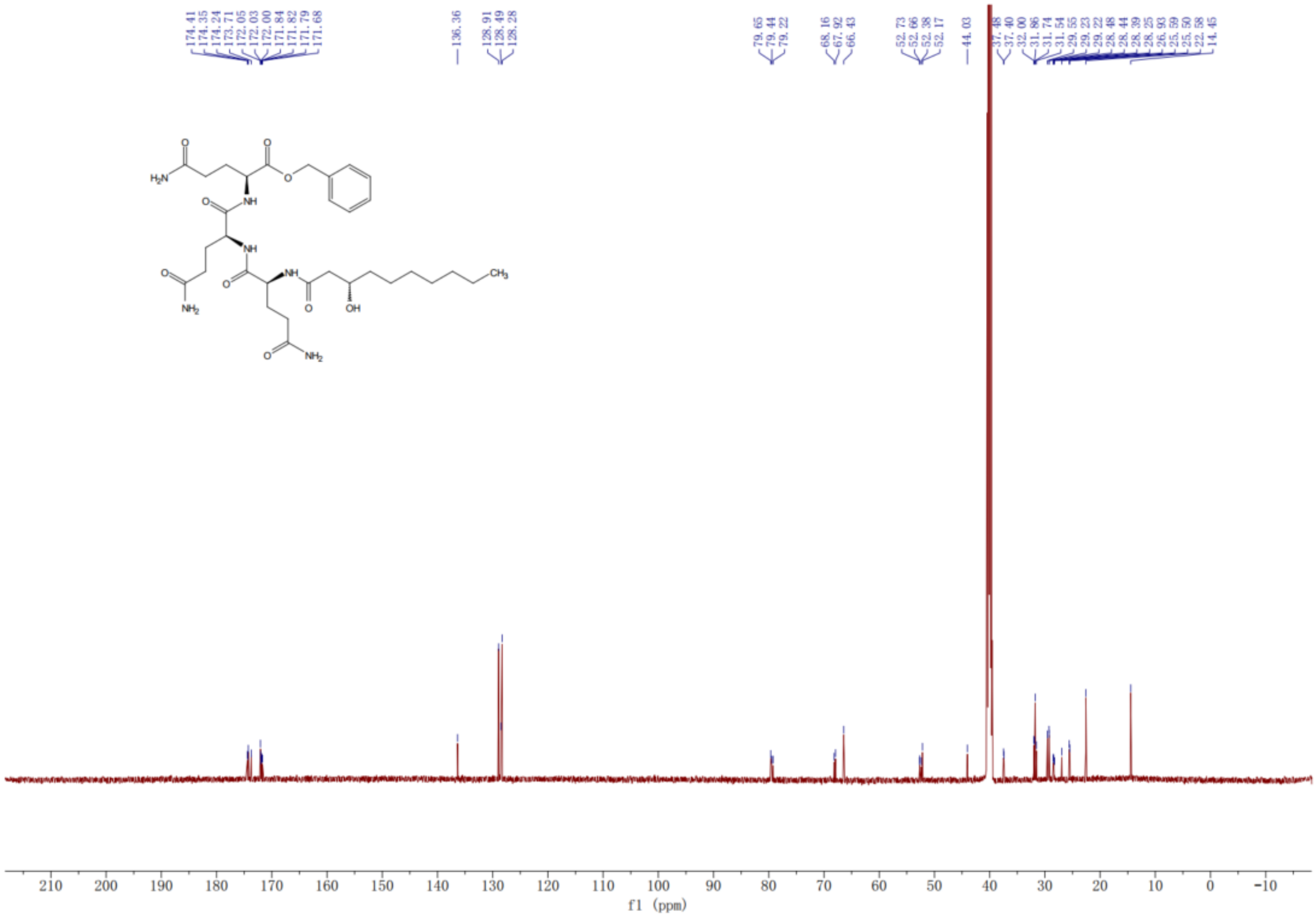

Figure S29. ^{13}C NMR spectrum of compound **19** (600 MHz). Solvent: DMSO-*d6*

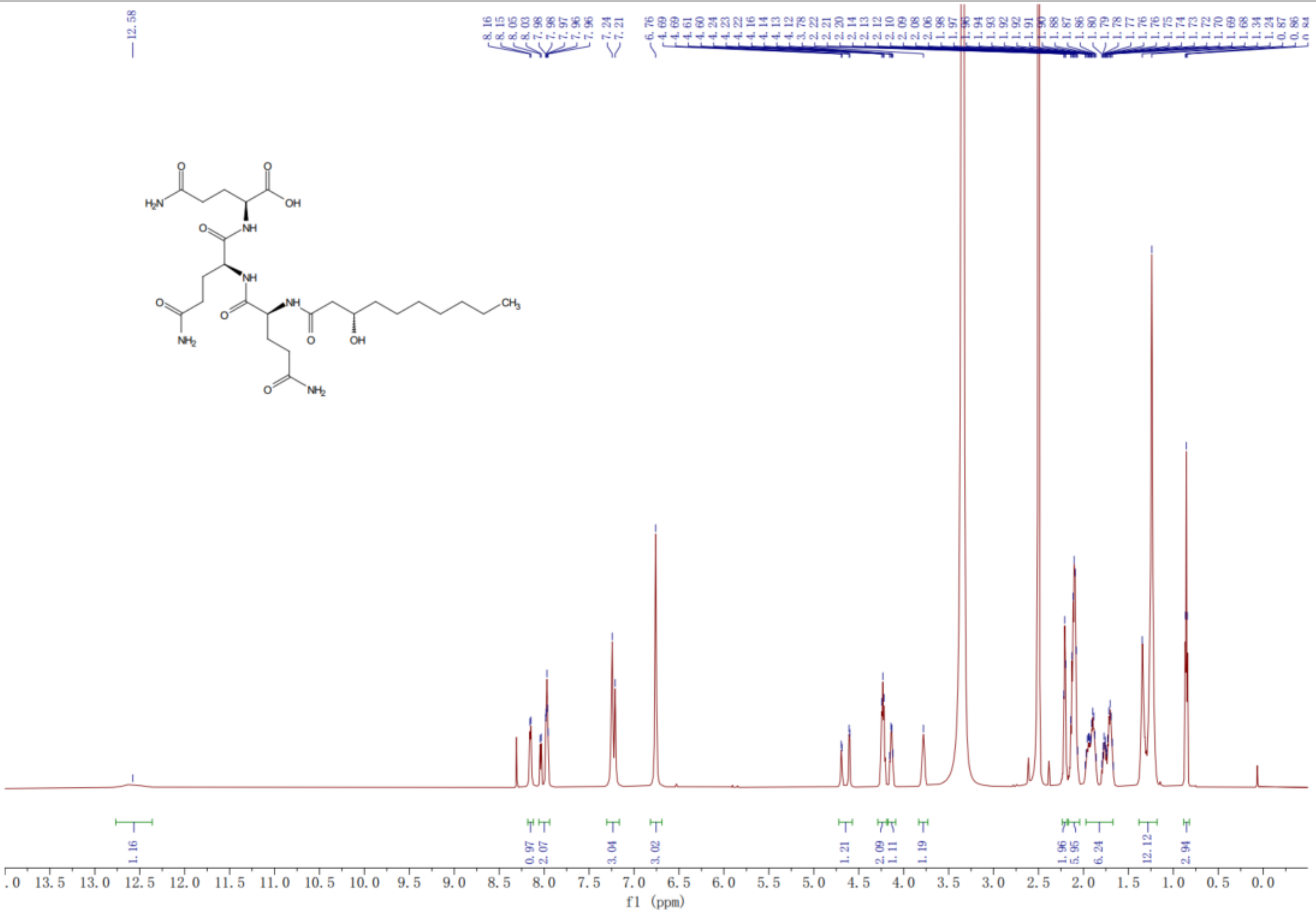

Figure S30. ^{1}H NMR spectrum of compound **20** (600 MHz). Solvent: DMSO-*d6*

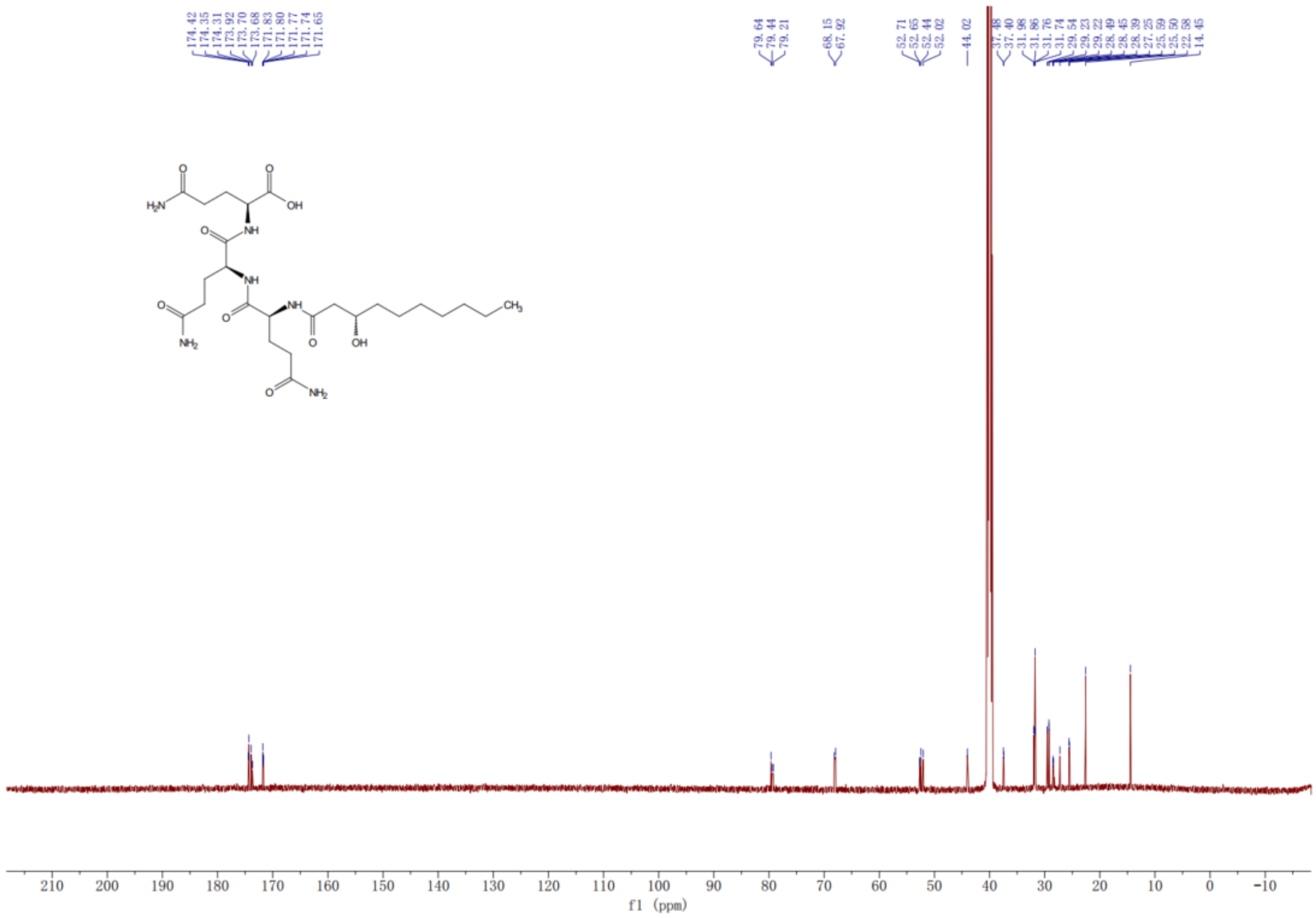

Figure S31. ^{13}C NMR spectrum of compound **20** (600 MHz). Solvent: DMSO-*d6*

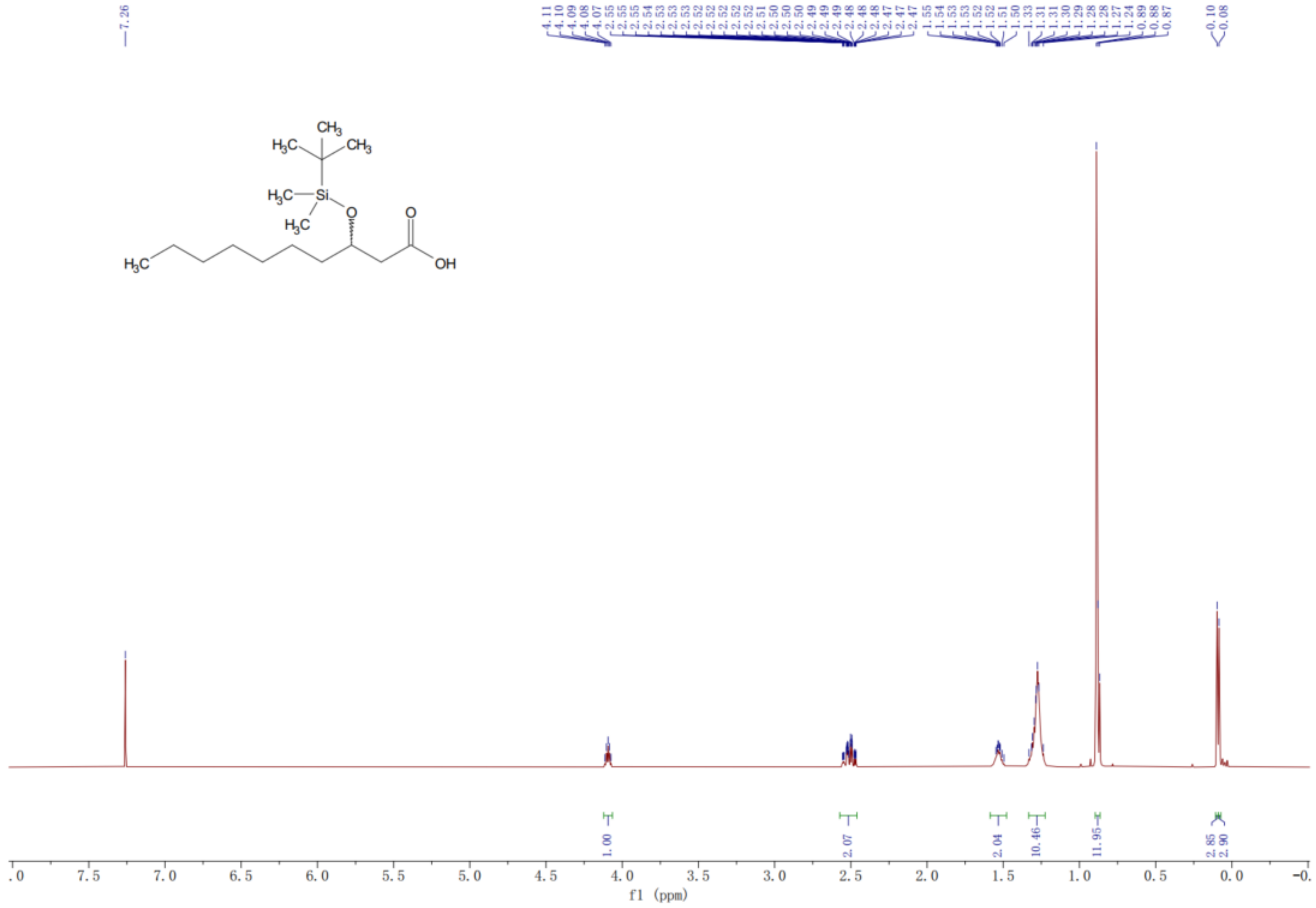

Figure S32. ^{1}H NMR spectrum of compound **22** (600 MHz). Solvent: DMSO-*d6*

References

[1] M. De Vleeschouwer, D. Sinnaeve, J. Van den Begin, T. Coenye, J. C. Martins, A. Madder, *Chemistry* **2014**, *20*, 7766-7775.

[2] N. Oberg, R. Zallot, J. A. Gerlt, *Journal of Molecular Biology* **2023**, *435*, 168018.

[3] K. Blin, S. Shaw, H. E Augustijn, Z. L. Reitz, F. Biermann, M. Alanjary, A. Fetter, B. R. Terlouw, W. W. Metcalf, E. J. N. Helfrich, G. P. van Wezel, M. H. Medema, T. Weber, *Nucleic Acids Research* **2023**, *51*, W46-W50.

[4] M. Mirdita, K. Schütze, Y. Moriwaki, L. Heo, S. Ovchinnikov, M. Steinegger, *Nature Methods* **2022**, *19*, 679-682.

[5] J. J. Zhang, X. Tang, T. Huan, A. C. Ross, B. S. Moore, *Nature Chemical Biology* **2020**, *16*, 42-49.

[6] J.-M. Reyrat, V. Pelicic, B. Gicquel, R. J. I. Rappuoli, *Immunity* **1998**, *66*, 4011-4017.

[7] J. Hallgren, K. D. Tsirigos, M. D. Pedersen, J. J. Almagro Armenteros, P. Marcatili, H. Nielsen, A. Krogh, O. Winther, *bioRxiv* **2023**, doi: https://doi.org/10.1101/2022.04.08.487609.
